# From Synthesis to Mechanism: Biological Evaluation of a *p*-Toluidine-Based Thiazolidinone-Quinoline VEGFR-2 Candidate Supported by CADD

**DOI:** 10.3390/ijms27073018

**Published:** 2026-03-26

**Authors:** Emad Manni, Modather F. Hussein, Sara Elkady, Adel A.-H. Abdel-Rahman, Mohamed A. Hawata, Wael A. El-Sayed, Ahmed F. El-Sayed, Hagar S. El-Hema

**Affiliations:** 1 Department of Clinical Laboratory Sciences, College of Applied Medical Sciences, Jouf University, Sakaka 72388, Saudi Arabia; emena@ju.edu.sa; 2 Chemistry Department, College of Science, Jouf University, P.O. Box 2014, Sakaka 72341, Saudi Arabia; 3 Chemistry Department, Faculty of Science, Menoufia University, Shebin El-Kom 32511, Egypt; sara.adel1700067@science.menofia.edu.eg (S.E.); adelnassar63@yahoo.com (A.A.-H.A.-R.); drmohamedhwata@gmail.com (M.A.H.); 4 Photochemistry Department, National Research Centre, Dokki, Giza 12622, Egypt; waelshendy@gmail.com; 5 Microbial Genetics Department, Biotechnology Research Institute, National Research Centre, Giza 12622, Egypt; ahmedfikry.nrc@gmail.com; 6 Egypt Center for Research and Regenerative Medicine (ECRRM), Cairo 11517, Egypt; 7 Basic Science Department (Chemistry), Thebes Higher Institute for Engineering, Thebes Academy, Maadi 11434, Egypt

**Keywords:** computer-aided drug discovery (CADD), molecular docking, molecular dynamics simulation, density functional theory (DFT), VEGFR-2 inhibition, *p*-toluidine-based thiazolidinone-quinoline hybrids, lead optimization

## Abstract

In response to recent advances in computer-aided drug discovery (CADD) enabled by high-performance computing, computational approaches were employed to support and rationalize the investigation of a VEGFR-2-targeted anticancer candidate, combining molecular-level modeling with experimental validation. Initial in silico ADMET profiling and molecular docking were conducted to support the evaluation of drug-like properties and target engagement within a series of *para*-toluidine-based derivatives (**1**–**14**). The most biologically active compound was further evaluated through 100 ns molecular dynamics simulations and comprehensive DFT calculations to investigate binding stability and electronic characteristics. Based on a rational design strategy and supported by computational analyses, the compounds were synthesized and fully characterized using IR, MS, ^1^H/^13^C NMR, and elemental analysis. Biological evaluation was performed against HepG-2, MCF-7, HCT-116, and normal WI-38 cells. Mechanistic studies included VEGFR-2 inhibition, wound-healing migration assays, cell-cycle distribution analysis, apoptosis assessment, and caspase-3 activation. Several derivatives exhibited micromolar cytotoxic activity, with compound **14** emerging as the most active against HepG-2 cells (IC_50_ = 7.84 ± 0.5 µM), showing cytotoxic activity comparable to that of sorafenib (IC_50_ = 9.18 ± 0.6 µM) and demonstrating favorable selectivity toward normal WI-38 cells (IC_50_ = 67.75 ± 3.6 µM). Compound **14** showed moderate VEGFR-2 inhibitory activity (IC_50_ = 0.55 µM), significant suppression of cell migration, pronounced G_0_/G_1_ cell-cycle arrest, and robust apoptosis induction supported by caspase-3 activation. Molecular docking and MD simulations supported a stable binding mode within the VEGFR-2 active site. This integrated framework highlights compound **14** as a selectively active VEGFR-2-oriented anticancer candidate scaffold with a favorable selectivity profile, supported by experimental and computational analyses, warranting further lead optimization.

## 1. Introduction

Cancer remains one of the leading causes of mortality worldwide and constitutes a major health burden due to its complex biology, rapid progression, and frequent resistance to conventional therapies [1,2,3,4]. Despite continuous advances in chemotherapy and targeted treatment strategies, many malignancies still exhibit limited therapeutic efficacy, the emergence of drug resistance, and significant adverse effects, highlighting the urgent need for more effective and selective anticancer agents [5,6,7,8,9,10,11]. Among these malignancies, hepatocellular carcinoma (HCC) represents the most prevalent primary liver cancer and continues to pose a major global health challenge [12,13,14,15,16,17,18,19]. Recent global assessments indicate that liver cancer accounts for nearly one million new cases and more than 750,000 deaths annually, underscoring the substantial and persistent worldwide burden of HCC [20,21,22,23,24,25]. In endemic regions, including Egypt and parts of Africa, chronic liver insults associated with parasitic infections have historically contributed to hepatic inflammation and fibrosis, thereby increasing susceptibility to HCC [26,27,28,29,30,31]. In particular, chronic infection with *Schistosoma mansoni* has been implicated in hepatocarcinogenesis, potentially promoting tumor development through sustained inflammatory and fibrotic processes [32]. Despite improvements in the management of underlying liver diseases and the introduction of systemic therapies, both the incidence and mortality of HCC continue to rise [33,34,35,36]. Sorafenib, a multikinase inhibitor long considered a first-line therapeutic option, frequently fails to achieve durable clinical responses due to tumor heterogeneity, acquired resistance mechanisms, and systemic toxicities [37,38,39,40,41,42]. Although recent meta-analyses have reported modest improvements in overall survival, with median values increasing from approximately 9.8 to 13.4 months, long-term outcomes remain largely unsatisfactory [38]. Collectively, these challenges, together with the predominance of late-stage diagnosis, emphasize the critical need for the development of novel, more effective, and selective targeted therapies for HCC management [39,40,41,42,43,44,45].

Tumor angiogenesis plays a pivotal role in the progression and dissemination of hepatocellular carcinoma (HCC), with vascular endothelial growth factor receptor-2 (VEGFR-2) recognized as the principal mediator of this process [46,47,48,49,50]. Activation of VEGFR-2 drives endothelial cell proliferation, migration, and neovascularization, thereby facilitating tumor growth and metastatic spread. Accordingly, VEGFR-2 has become a validated molecular target in anticancer drug discovery, and its inhibition represents a cornerstone strategy in anti-angiogenic therapy [51,52,53,54,55,56,57].

Quinoline-based compounds have attracted considerable interest as anticancer agents, particularly for HCC treatment, owing to their structural versatility and broad biological activities [58,59,60,61,62,63,64,65,66,67,68,69,70]. The quinoline scaffold consists of a fused benzene–pyridine bicyclic system that confers favorable electronic and physicochemical properties for molecular recognition. The benzene ring enhances hydrophobic and π–π stacking interactions within receptor binding pockets, while the pyridine nitrogen functions as a hydrogen bond acceptor, enabling specific interactions with key residues in the ATP-binding hinge region of VEGFR-2. This combination of aromatic hydrophobicity and heteroatom functionality establishes quinoline as a privileged scaffold for kinase inhibition [71,72,73,74,75,76]. Numerous studies have demonstrated that quinoline derivatives exert pronounced antiproliferative effects through modulation of oncogenic signaling pathways, particularly via VEGFR-2 inhibition. Within the VEGFR-2 active site, the quinoline core typically acts as an ATP-competitive hinge-binding motif, forming stable interactions that suppress angiogenesis and limit tumor vascularization and progression. Nevertheless, despite extensive investigation of quinoline frameworks, further structural optimization remains essential to enhance VEGFR-2 selectivity and potency in anticancer drug discovery [77].

**Figure 1:**
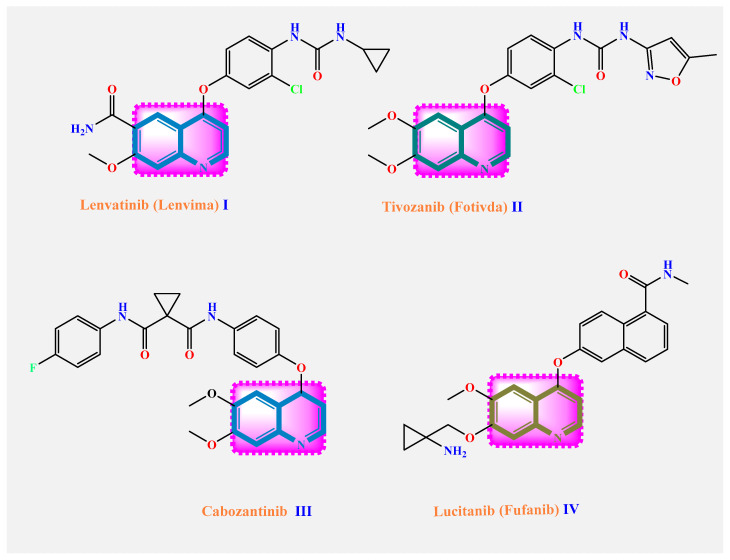
FDA-related VEGFR-2 inhibitors containing quinoline-based frameworks, structures of lenvatinib and tivozanib (FDA-approved) and lucitanib (currently under FDA regulatory evaluation).

Notably, the clinical relevance of quinoline-based scaffolds is supported by several FDA-approved anticancer agents that incorporate a true quinoline core, including lenvatinib (**I**) [78,79,80,81,82,83,84,85,86,87,88,89,90], tivozanib (**II**) [91,92,93,94], and cabozantinib (**III**) [95,96], all of which exert their therapeutic effects primarily through inhibition of VEGFR-mediated signaling pathways. In contrast, although lucitanib (**IV**) has frequently been discussed in this context and reported to possess a quinoline-related structural motif, it is not currently approved by the U.S. Food and Drug Administration (FDA). Instead, lucitanib is recognized as a potent multi-kinase inhibitor targeting VEGFR-1/2/3 and FGFR1/2, with regulatory approval granted in certain markets, such as China under alternative trade names (e.g., Fufanib), while FDA approval remains pending or under regulatory evaluation [97,98,99,100,101,102] (Figure 1).

In parallel, several FDA-approved VEGFR inhibitors, including sorafenib [103], regorafenib [104], ramucirumab [105], and fruquintinib [106,107,108]—a quinazoline-based agent approved in China—further validate VEGFR signaling as a clinically relevant therapeutic target. Notably, these small-molecule inhibitors share heteroaromatic scaffolds that closely resemble the canonical quinoline framework, preserving a largely planar aromatic pharmacophore with only minor peripheral modifications. This pronounced structural similarity emphasizes that quinoline-like chemotypes constitute one of the most privileged and repeatedly exploited cores in VEGFR-targeted drug design, reinforcing the medicinal importance of quinoline-based architectures. In addition, the clinical approval of the monoclonal antibody ramucirumab as a selective VEGFR-2 antagonist further highlights VEGFR-2 as a highly druggable and therapeutically significant target in tumor angiogenesis and cancer progression.

**Figure 2:**
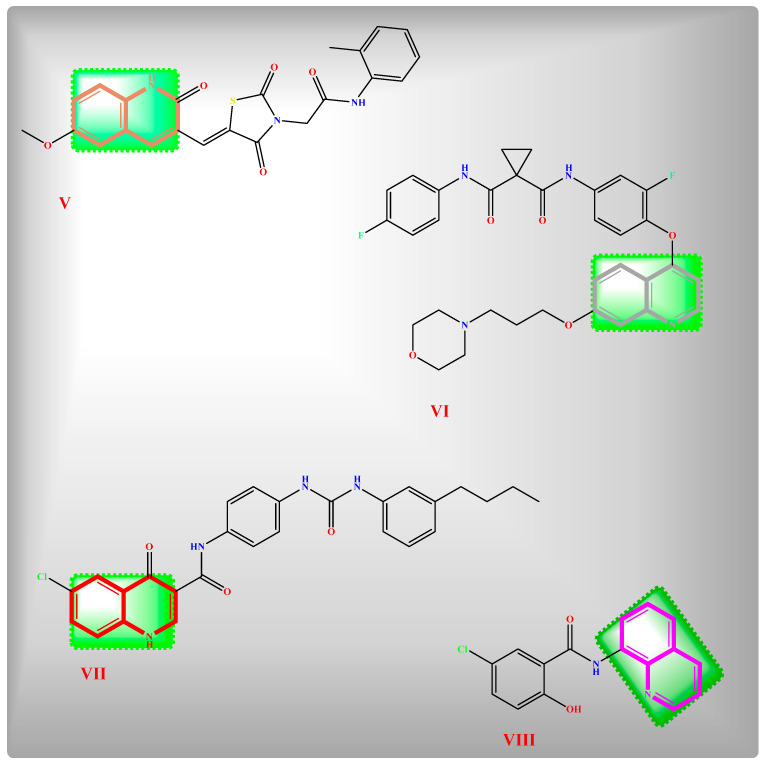
Structures of quinoline-based VEGFR-2 inhibitors **V**, **VI** (foretinib), **VII**, and **VIII** with their corresponding IC_50_ values.

Previous studies have reported a wide range of quinoline-based derivatives exhibiting VEGFR-2 inhibitory activity, functioning as ATP-competitive inhibitors that engage the hinge region and demonstrate antiproliferative effects across various cancer cell lines, including hepatocellular carcinoma models (Figure 2).

### 1.1. Role of Thiazolidin-4-one and Morpholine Moieties in VEGFR-2 Inhibitor Design

**Figure 3:**
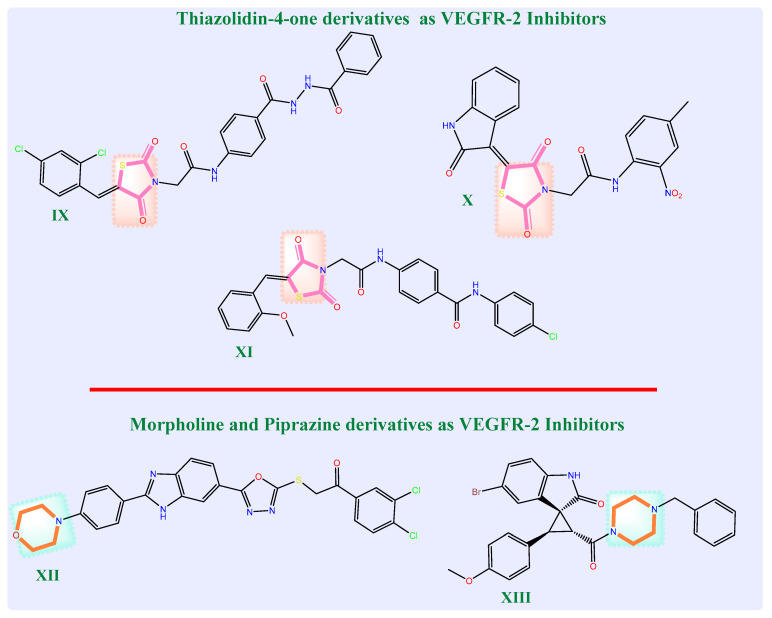
Thiazolidin-4-one (**IX**–**XI**) and morpholine-based (**XII**–**XIII**) derivatives showing strong VEGFR-2 inhibition, with activities comparable to or exceeding standard inhibitors in HepG-2 cells.

In parallel, thiazolidin-4-one-based scaffolds have been recognized as effective VEGFR-2 inhibitors due to their heterocyclic framework, which enables favorable interactions within the VEGFR-2 active site and contributes to the suppression of angiogenic signaling and induction of apoptosis. In addition, morpholine-containing derivatives have been shown to enhance solubility, polarity, and ligand orientation toward solvent-exposed regions of VEGFR-2, thereby supporting their role as privileged motifs in VEGFR-2-targeted inhibitor design. Collectively, these structural classes highlight the potential of quinoline, thiazolidinone, and morpholine pharmacophores as valuable building blocks for the development of VEGFR-2-oriented anticancer agents (Figure 3).

Representative VEGFR-2 inhibitory activities of selected reference compounds and sorafenib reported in the literature are summarized in Table 1.

### 1.2. Rational and Study Outline

The development of compound **14** was guided by a rational hybridization strategy integrating key structural features of the reference inhibitors **XIV** [115] and **XII** [117], which exhibited VEGFR-2 inhibitory activities of 87.37 nM (1.63× sorafenib) and 49.0 nM (1.32× sorafenib), respectively. Although **XIV** possessed a quinolinone–thiazolidinone core capable of partial hinge binding, its overall affinity was limited by weak solvent interaction, a rigid C=N linker, and an inactive *p*-tolyl acetamide tail. To explore potential structure-activity relationships within this scaffold, a methyl group was introduced to reinforce hinge anchoring; the quinolinone carbonyl was replaced with a thiophene ring to enhance hydrophobic pocket occupancy; an ester substituent was incorporated to promote solvent–front interactions; and the C=N linkage was converted into a direct bond to improve catalytic pocket accommodation. In addition, the poorly oriented *p*-tolyl acetamide unit was replaced with a flexible (CH_2_)_3_-morpholine side chain, increasing polarity, solubility, and the potential for water-mediated interactions.

**Figure 4:**
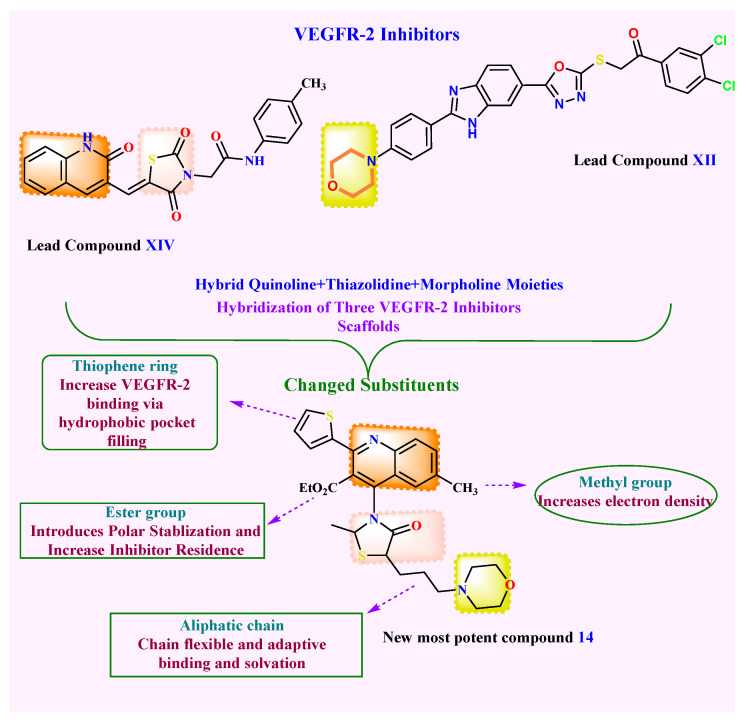
Rational design of compound **14** through hybridization of reference inhibitors **XIV** and **XII**. Combining the hinge-binding core of **XIV** with the solvent-oriented morpholine fragment of **XII** yielded the optimized hybrid scaffold **14**.

In the morpholine-based reference compound **XII**, the morpholine ring is embedded within a rigid phenyl-linked framework, which may restrict its optimal orientation toward the solvent-exposed region of VEGFR-2. In compound **14**, repositioning the morpholine moiety onto a flexible aliphatic linker was intended to reduce steric constraints and allow greater conformational adaptability at the solvent front. The rational hybridization of the hinge-interacting motif of **XIV** with the solvent-exposed morpholine fragment of **XII** was therefore pursued to generate compound **14**. This design strategy was aimed at exploring whether the combination of deep ATP-pocket engagement and enhanced solvent–front interactions could afford a balanced VEGFR-2 interaction profile, providing a structural basis for the observed biological activity relative to the individual parent frameworks (Figure 4).

Although compound **14** exhibited lower VEGFR-2 inhibitory potency than the parent inhibitors **XII** and **XIV** as well as sorafenib, the present hybrid design was primarily intended to explore the structural compatibility between the hinge-binding framework of **XIV** and the solvent-exposed morpholine fragment of **XII**, thereby providing additional insight into the structure-activity relationships of this scaffold.

## 2. Results and Discussion

### 2.1. Chemistry

**Scheme 1:**
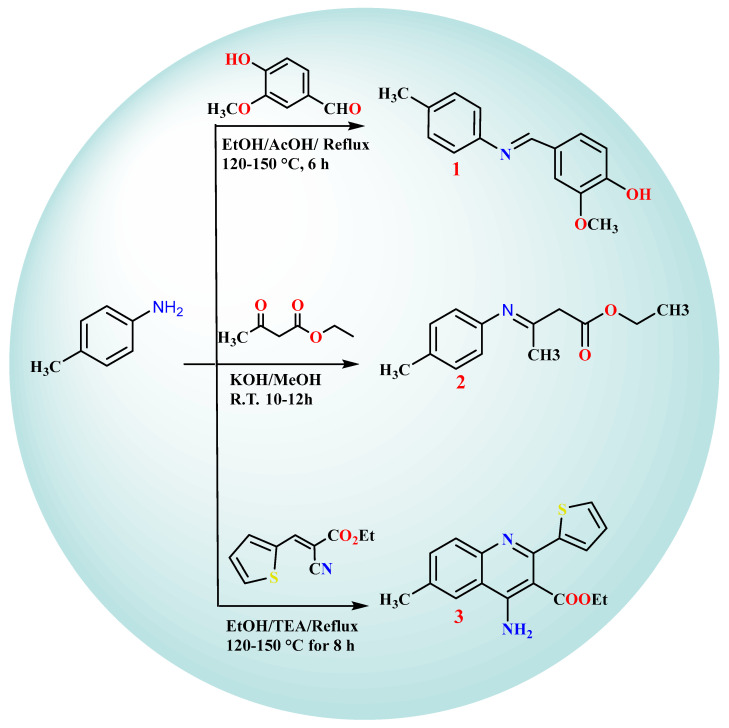
Synthetic routes illustrating the reactions of *p*-toluidine with different electrophilic reagents leading to the formation of compounds **1**–**3** via Schiff base formation, Knoevenagel-type condensation, and Michael-induced cyclization.

Scheme 1 illustrates the independent reactions of *p*-toluidine with three electrophilic partners, demonstrating the nucleophilic reactivity of the aniline nitrogen toward structurally diverse substrates. Condensation with vanillin afforded the Schiff base **1** through nucleophilic attack of the amine on the aldehydic carbon followed by dehydration. Reaction with ethyl acetoacetate proceeded via nucleophilic addition to the activated carbonyl group under basic conditions and subsequent proton rearrangement consistent with a Knoevenagel-type process to yield the imine ester **2**. In contrast, treatment with ethyl cyanoarylidine involved a Michael-type 1,4-addition to the α,β-unsaturated system followed by intramolecular cyclization and aromatization to furnish the quinoline derivative **3**. These reactions represent three distinct pathways including azomethine formation, C–C bond construction, and cyclocondensation.

The structure of **1** was confirmed by spectroscopic analysis. The IR spectrum showed a phenolic OH band at 3421 cm^−1^, aromatic C–H absorptions at 3064 and 3010 cm^−1^, aliphatic C–H bands at 2926 and 2858 cm^−1^, an azomethine C=N band at 1661 cm^−1^, aromatic C=C bands at 1600 and 1511 cm^−1^, and a methoxy C–O band at 1273 cm^−1^. In the ^1^H NMR spectrum, singlets at δ 2.35 and 3.85 ppm corresponded to the *p*-methyl and methoxy protons, respectively, while aromatic protons appeared at δ 7.06–7.47 ppm. The azomethine proton resonated at δ 8.86 ppm, and the phenolic OH proton appeared at δ 9.50 ppm. The ^13^C NMR spectrum showed the azomethine carbon at δ 159.75 ppm, methoxy and methyl carbons at δ 59.33 and 21.08 ppm, aromatic carbons at δ 115.71–139.58 ppm, and substituted aromatic carbons at δ 147.12, 149.13, and 151.78 ppm. Formation of compound **2** was confirmed by the disappearance of the NH_2_ band in the IR spectrum and the appearance of a strong ester C=O band at 1747 cm^−1^ and an azomethine C=N band at 1637 cm^−1^, together with aliphatic C–H stretching at 2982–2864 cm^−1^. The ^1^H NMR spectrum displayed methyl signals at δ 1.36 and 1.78 ppm corresponding to the ethyl ester and imine-associated methyl groups, respectively, while methylene protons appeared at δ 3.06 and 4.51 ppm. The ^13^C NMR spectrum showed the ester carbonyl carbon at δ 166.05 ppm, the imine carbon at δ 163.02 ppm, and the newly introduced methyl and methylene carbons at δ 12.12, 20.31, 55.19, and 63.07 ppm. Similarly, compound **3** was confirmed by spectroscopic data. The IR spectrum displayed NH_2_ bands at 3425 and 3415 cm^−1^, an ester carbonyl band at 1718 cm^−1^, and a quinoline C=N band at 1597 cm^−1^. In the ^1^H NMR spectrum, the ethyl ester group appeared as a triplet at δ 1.03 ppm and a quartet at δ 4.36 ppm, while the aromatic methyl signal appeared at δ 2.30 ppm. The NH_2_ protons appeared as a broad singlet at δ 6.02 ppm, and aromatic protons resonated at δ 7.16–7.52 ppm. The ^13^C NMR spectrum showed the ester carbonyl carbon at δ 166.23 ppm, ethyl ester carbons at δ 14.92 and 59.80 ppm, quinoline C=N at δ 156.53 ppm, the pyridine carbon attached to NH_2_ at δ 158.00 ppm, and the pyridine carbon adjacent to the ester group at δ 103.97 ppm.

**Scheme 2:**
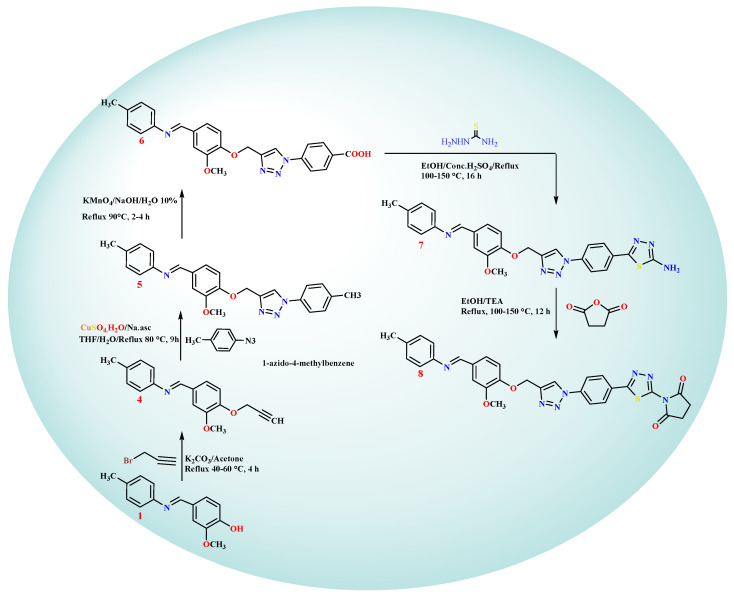
Stepwise synthesis of propargylated derivative **4** and triazole-based derivatives **5**–**8** from compound **1** via O-alkylation, click cycloaddition, oxidation, thiadiazole formation, and succinimide cyclization.

Scheme 2 shows the stepwise modification of the imine precursor **1** to furnish derivatives **4**–**8** through sequential functional transformations.

O-Propargylation of **1** with propargyl bromide afforded the terminal alkyne **4**, evidenced by disappearance of the phenolic OH signal of **1** and appearance of new alkyne absorptions at 3304 cm^−1^ (≡C–H) and 2254 cm^−1^ (C≡C) in the IR spectrum. The ^1^H NMR spectrum displayed new propargyl signals at δ 3.70 ppm (≡C–H) and δ 4.22 ppm (O–CH_2_). Correspondingly, the ^13^C NMR spectrum showed new sp carbons at δ 75.19 and 79.13 ppm together with the methylene carbon at δ 63.00 ppm, confirming formation of **4**. Cycloaddition of **4** with 1-azido-4-methylbenzene under CuAAC conditions produced the 1,2,3-triazole derivative **5**, accompanied by disappearance of the alkyne signals of **4**. The IR spectrum showed bands at 1633 cm^−1^ (C=N) and 1398 cm^−1^ (N=N). In the ^1^H NMR spectrum, singlets appeared at δ 2.45 ppm (Ar-CH_3_) and δ 8.02 ppm (triazole-H), while the ^13^C NMR spectrum displayed triazole carbons at δ 115.24 and 139.83 ppm. The EI-MS spectrum showed *m*/*z* 412.53 (M^+^) with a base peak at *m*/*z* 134. Oxidation of **5** with KMnO_4_ converted the benzylic methyl group into the carboxylic acid derivative 6, as indicated by disappearance of the CH_3_ signals of 5. The IR spectrum exhibited O–H at 3481 cm^−1^ and C=O at 1721 cm^−1^, while the ^1^H NMR spectrum showed a new COOH signal at δ 12.12 ppm. The ^13^C NMR spectrum displayed the carboxyl carbon at δ 160.03 ppm. Cyclocondensation of **6** with thiosemicarbazide produced the 1,3,4-thiadiazole derivative **7**, accompanied by disappearance of the COOH signals of 6. The IR spectrum showed NH_2_ bands at 3487 and 3447 cm^−1^ as well as C=N bands at 1634 and 1627 cm^−1^. The ^1^H NMR spectrum displayed NH_2_ protons at δ 6.02 ppm, while the ^13^C NMR spectrum showed thiadiazole carbons at δ 160.00 and 171.92 ppm. Finally, acylation of **7** with succinic anhydride afforded the succinimide derivative **8**, confirmed by disappearance of NH_2_ bands and appearance of a new 1715 cm^−1^ (2C=O) absorption. The ^1^H NMR spectrum showed new methylene signals at δ 3.14 ppm (2CH_2_), while the ^13^C NMR spectrum displayed carbonyl carbons at δ 175.22 and 176.08 ppm as well as a methylene carbon at δ 30.00 ppm. The EI-MS spectrum showed *m*/*z* 579.81 (M^+^) with a base peak at *m*/*z* 294.44.

**Scheme 3:**
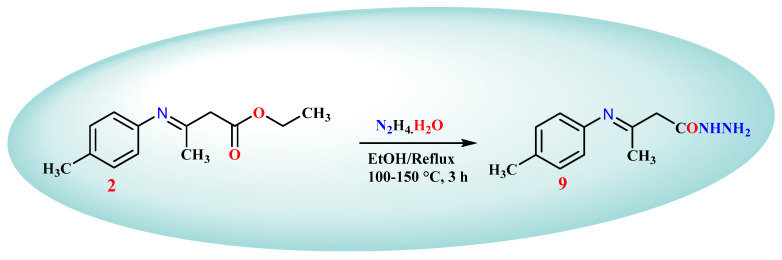
Synthesis of compound **9** from ester derivative **2** via hydrazinolysis through a nucleophilic acyl substitution mechanism.

Scheme 3 shows the conversion of ester **2** into hydrazide **9** via hydrazinolysis with hydrazine hydrate (N_2_H_4_·H_2_O) under reflux in ethanol, proceeding through nucleophilic acyl substitution with elimination of the ethoxy group. Formation of **9** was confirmed by disappearance of the ester signals of **2**. The IR spectrum showed an amide C=O band at 1703 cm^−1^ and new NH/NH_2_ stretching bands at 3417, 3388, and 3228 cm^−1^. The ^1^H NMR spectrum displayed exchangeable signals at δ 4.24 ppm (NH_2_) and δ 9.28 ppm (NH). The ^13^C NMR spectrum showed the amide carbonyl carbon at δ 176.11 ppm, with disappearance of the ester carbon signals.

**Scheme 4:**
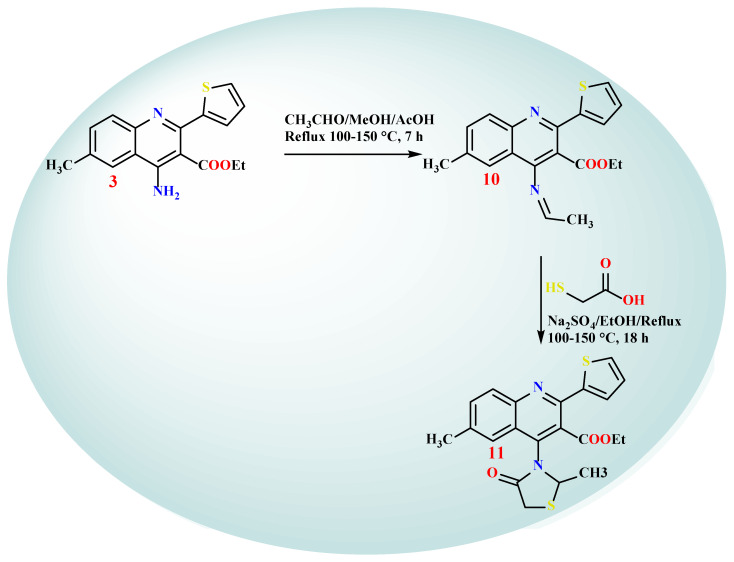
Transformation of compound **3** into compounds **10** and **11** via Schiff base formation followed by thiazolidinone cyclization.

Scheme 4 describes the transformation of compound **3** into derivative **11** through two successive steps. Condensation of **3** with acetaldehyde in the presence of acetic acid afforded the Schiff base **10**, while subsequent reaction with thioglycolic acid in the presence of sodium sulfate resulted in cyclocondensation to give the thiazolidinone derivative **11**. Formation of **10** was evidenced by disappearance of the NH_2_ stretching bands present in compound **3**, confirming its participation in condensation. The IR spectrum showed a new C=N band at 1566 cm^−1^. In the ^1^H NMR spectrum, a new imine proton appeared at δ 8.70 ppm, together with a methyl doublet at δ 0.92 ppm corresponding to –CH_3_–C=N. The ^13^C NMR spectrum displayed a new imine carbon at δ 162.22 ppm and a methyl carbon at δ 10.01 ppm.

In contrast, cyclization of **10** to **11** was confirmed by disappearance of the imine methyl fragment (–CH=N–CH_3_) signals of 10. The IR spectrum showed a new C=O band at 1664 cm^−1^ characteristic of the thiazolidinone ring. The ^1^H NMR spectrum displayed new signals at δ 4.00 ppm (CH_2_) and δ 4.89 ppm (CH) for the thiazolidinone ring, along with a methyl signal at δ 1.85 ppm. Correspondingly, the ^13^C NMR spectrum showed a carbonyl carbon at δ 170.03 ppm, together with signals at δ 35.03 ppm (CH_2_), δ 56.00 ppm (CH), and δ 25.22 ppm (CH_3_), confirming formation of compound **11**.

**Scheme 5:**
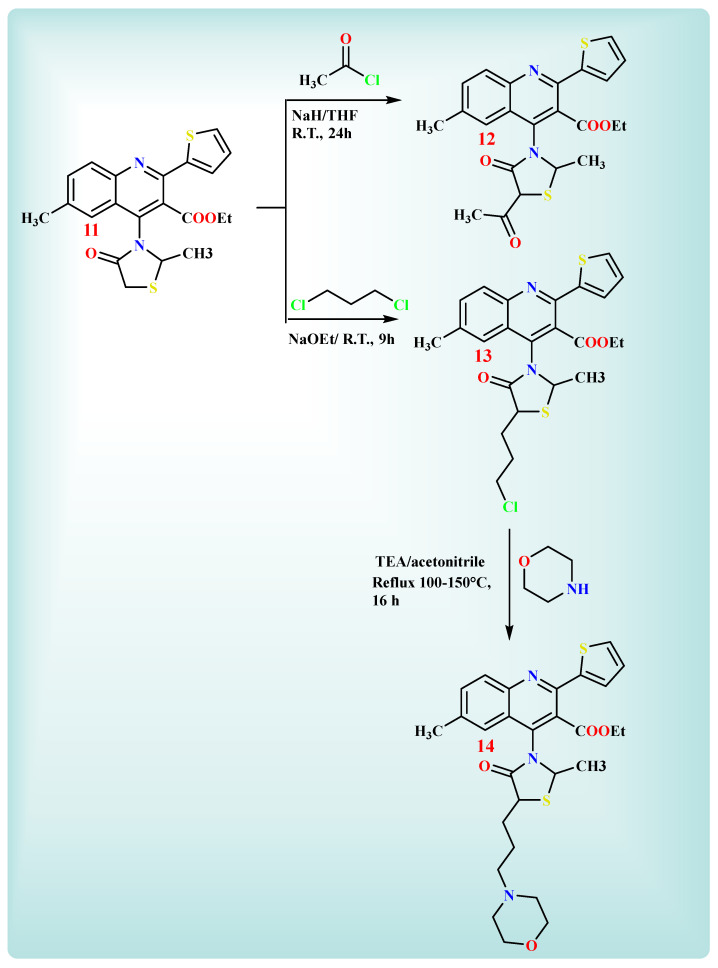
Stepwise functionalization of the thiazolidinone scaffold via acylation, alkylation, and morpholine substitution to afford derivatives **12**–**14**.

Scheme 5 shows the post-functionalization of compound **11** to derivatives **12**–**14**. Acetylation of **11** with acetyl chloride afforded **12**, while alkylation with 1,3-dichloropropane produced the chloroalkyl derivative **13**, which subsequently underwent nucleophilic substitution with morpholine to give **14**.

Formation of **12** was indicated by disappearance of the thiazolidinone CH_2_ signal of **11** and appearance of a methine CH center. The IR spectrum showed a new C=O band at 1708 cm^−1^. The ^1^H NMR spectrum displayed signals at δ 2.48 ppm (CH_3_CO) and δ 4.24 ppm (CH), while the ^13^C NMR spectrum showed δ 196.82 ppm (C=O), 29.97 ppm (CH_3_), and 67.57 ppm (CH). Formation of **13** was confirmed by a C–Cl band at 765 cm^−1^. The ^1^H NMR spectrum showed methylene signals at δ 1.30, 2.01, and 3.89 ppm (–CH_2_–CH_2_–CH_2_–Cl), while the ^13^C NMR spectrum displayed δ 29.94, 31.00 ppm, and 43.33 ppm (CH_2_–Cl). The EI–MS spectrum showed *m*/*z* 492.45 (M^+^). Substitution of the chloro group in **13** afforded **14**, evidenced by disappearance of the C–Cl band (~765 cm^−1^). The ^1^H NMR spectrum showed morpholine signals at δ 2.70 ppm (N–CH_2_) and 3.73 ppm (O–CH_2_), with the CH_2_–Cl signal shifting from δ 3.89 to δ 2.84 ppm. The ^13^C NMR spectrum showed morpholine carbons at δ 58.10 ppm (N–CH_2_) and 67.13, 69.95 ppm (O–CH_2_). The EI–MS spectrum showed *m*/*z* 539.02 (M^+^) with a base peak at *m*/*z* 508.23.

Following the successful synthesis and structural characterization of the prepared heterocyclic systems, their biological performance was evaluated through in vitro anticancer screening. All synthesized compounds were assessed for cytotoxic activity to explore the relationship between their structural features and biological behavior. In this section, the anticancer results are presented and discussed with emphasis on the impact of structural variations, functional group modifications, and heterocyclic framework complexity on the observed cytotoxic profiles, aiming to elucidate structure-activity relationships and identify promising anticancer lead candidates.

### 2.2. Biological Evaluation

#### 2.2.1. Antiproliferative In Vitro Potency

The antiproliferative activity of the synthesized compounds (**1**–**14**) was evaluated against HCT-116, HepG-2, and MCF-7 human cancer cell lines, alongside normal WI-38 lung fibroblast cells to assess selectivity. The tested compounds exhibited a broad range of micromolar IC_50_ values, with several derivatives showing activities comparable to doxorubicin (Table 2) detailed cytotoxicity percentages at the tested concentrations are provided in Table S1. Among the series, compound **14** emerged as the most active derivative against HepG-2 cells, while the corresponding dose–response curves are presented in Figure S47 (IC_50_ = 7.84 ± 0.5 µM), showing cytotoxic activity comparable to that of sorafenib (IC_50_ = 9.18 ± 0.6 µM).

Compound **2** also demonstrated strong antiproliferative activity, particularly against MCF-7 cells (IC_50_ = 6.31 ± 0.4 µM), showing cytotoxic activity comparable to both doxorubicin (IC_50_ = 4.17 ± 0.2 µM) and sorafenib (IC_50_ = 7.26 ± 0.3 µM), while compound **4** showed notable inhibition of MCF-7 proliferation (IC_50_ = 8.72 ± 0.7 µM). In contrast, most compounds exhibited weak activity against HCT-116 cells, with compound **2** displaying the highest, albeit moderate, efficacy.

Compounds **6**, **9**, **11**, and **13** showed moderate antiproliferative activity relative to the reference drugs doxorubicin and sorafenib, with IC_50_ values ranging from 18.17 to 59.44 µM across the tested cancer cell lines, whereas the remaining derivatives (**1**, **3**, **5**, **7**, **8**, **10**, and **12**) were weakly active or inactive (IC_50_ > 60 µM). Importantly, evaluation against normal WI-38 cells revealed that the most active compounds (**2**, **4**, and **14**) exhibited substantially lower cytotoxicity (IC_50_ = 49.53 ± 3.0, 55.48 ± 3.2, and 67.75 ± 3.6 µM, respectively) compared to doxorubicin (IC_50_ = 6.72 ± 0.5 µM), indicating a favorable selectivity profile.

Selectivity index analysis further highlighted compound **14** as the most selective toward HepG-2 cells (SI = 8.6), while compound **2** showed significant selectivity across MCF-7, HCT-116, and HepG-2 cell lines. Collectively, these results identify compounds **2** and **14** as the most promising antiproliferative leads within the investigated series based on their balanced activity and selectivity profiles.

##### Mechanistic Insight and Perspectives

Based on its comparable antiproliferative activity and favorable selectivity profile observed in the MTT assay, compound **14** was identified as a promising lead candidate against hepatocellular carcinoma cells. To elucidate its underlying mechanism of action, further investigations were directed toward evaluating its inhibitory activity against VEGFR-2, as well as its effects on cell-cycle progression and apoptosis induction. Additional mechanistic validation involved wound-healing assays to assess its impact on cancer cell migration and Western blot analysis of key apoptotic markers, particularly cleaved caspase-3.

Moreover, complementary in silico investigations, including molecular docking, molecular dynamics simulations, ADMET profiling, and density functional theory (DFT) calculations, were performed to rationalize and support the experimental observations, by evaluating binding stability, key molecular interactions, and drug-like properties.

##### Structure-Activity Relationship (SAR) of Anticancer Activity

The antiproliferative data (Table 3) revealed a clear structure-activity relationship governed by heterocyclic modification, electronic distribution, and lipophilic balance within the investigated scaffold series. The parent azomethine framework represented by compound **1** exhibited weak antiproliferative activity against HepG-2 cells (IC_50_ = 56.16 µM), indicating that the simple imine scaffold lacks sufficient pharmacophoric features required for efficient biological activity. Structural modification of this scaffold significantly influenced the biological profile. Incorporation of an imino-ester functionality in compound **2** led to improved activity relative to the parent structure, suggesting that the introduction of a carbonyl-containing moiety improves electronic distribution and provides an additional hydrogen-bond acceptor site that may facilitate interactions with residues within the biological target. The presence of carbonyl-containing heterocyclic motifs has frequently been associated with enhanced antiproliferative activity due to their ability to participate in key hydrogen-bonding interactions with biological targets [119].

Further scaffold optimization through propargyl ether substitution afforded compound **4**, which exhibited a pronounced increase in potency (IC_50_ = 19.53 µM, ΔpIC_50_ = 0.46). This improvement may be attributed to the introduction of the propargyl moiety, which increases lipophilicity and π-electron density, thereby enhancing potential hydrophobic and π–π interactions within the binding pocket of the biological target, a behavior that has been reported for related heterocyclic anticancer scaffolds [120]. This improvement can be attributed to the increased lipophilicity and π-electron density introduced by the alkyne fragment, which may promote hydrophobic and π-stacking interactions within the binding site and enhance ligand accommodation within hydrophobic regions of the target pocket. Subsequent diversification via Cu(I)-catalyzed azide–alkyne cycloaddition produced the triazole derivative **5**, which displayed reduced activity (IC_50_ = 42.85 µM, ΔpIC_50_ = −0.34, GE = −0.04), likely due to increased polarity and steric bulk that limit productive interactions with the biological target. Oxidation of the terminal methyl group yielded the carboxylic acid derivative **6** (IC_50_ = 36.15 µM, ΔpIC_50_ = 0.07, GE = 0.04), which provided only marginal improvement relative to compound **5**. Further heterocyclic diversification of compound **6** through cyclization with thiosemicarbazide generated the thiadiazole derivative **7**, a heterocycle widely reported in anticancer scaffolds [121]. However, compound **7** exhibited reduced potency (IC_50_ = 92.27 µM, ΔpIC_50_ = −0.41, GE = −0.08), suggesting that the increased polarity and rigidity of this heterocycle are unfavorable within the present scaffold series. Condensation with succinic anhydride afforded the succinimide derivative **8** (IC_50_ = 78.16 µM, ΔpIC_50_ = 0.08, GE = 0.01), which showed only a modest improvement compared with compound **7**. Conversion of the imino-ester functionality into the hydrazide derivative **9** resulted in decreased activity (IC_50_ = 59.44 µM, ΔpIC_50_ = −0.70, GE = −0.35), indicating that the highly polar hydrazide group negatively affects the biological profile. Subsequent condensation with acetaldehyde afforded the imine derivative **10**, which moderately improved activity (IC_50_ = 46.59 µM, ΔpIC_50_ = 0.27, GE = 0.09). Cyclization with mercaptoacetic acid generated the thiazolidinone derivative **11** [122], which exhibited slightly reduced potency (IC_50_ = 68.75 µM, ΔpIC_50_ = −0.17, GE = −0.03). Further modification of compound **11** through acetylation produced compound **12**, which displayed only a minor change in activity (IC_50_ = 61.78 µM, ΔpIC_50_ = 0.05, GE = 0.02). In contrast, alkylation with a chloropropyl chain afforded compound **13**, which significantly enhanced antiproliferative activity (IC_50_ = 20.02 µM, ΔpIC_50_ = 0.54, GE = 0.14), highlighting the beneficial effect of introducing flexible lipophilic substituents capable of interacting with hydrophobic regions of the biological target. Most importantly, replacement of the chloropropyl substituent with a morpholine moiety produced compound **14**, which emerged as the most potent derivative in the series against HepG-2 cells (IC_50_ = 7.84 µM, ΔpIC_50_ = 0.41, GE = 0.07). The enhanced activity likely arises from the morpholine ring providing an optimal balance between polarity and lipophilicity, improving aqueous solubility while facilitating favorable interactions within solvent-exposed regions of the binding pocket. Similar beneficial effects of morpholine incorporation have been widely reported in anticancer scaffolds due to its ability to enhance drug-like properties and target engagement [117,123,124,125,126].

**Figure 5:**
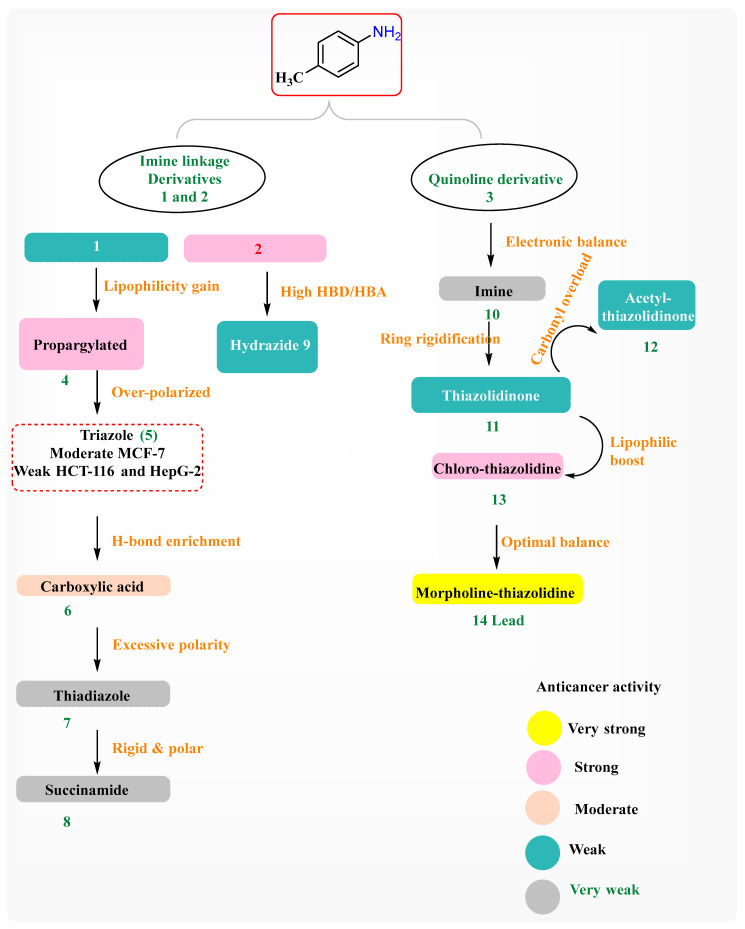
Structure-activity relationship (SAR) map illustrating the structural evolution of compounds **1**–**14** and the influence of key structural modifications on antiproliferative activity against HepG-2 cells. Color coding represents the relative potency of the derivatives based on their IC_50_ values. The scheme highlights the impact of lipophilicity, polarity, and ring rigidification on biological activity, culminating in the optimized morpholine derivative 14 identified as the lead compound.

Overall, these findings indicate that controlled modulation of polarity and lipophilicity, together with incorporation of flexible heterocyclic substituents, plays a key role in optimizing the antiproliferative activity of this scaffold series (Figure 5 and Table 3). Group efficiency (GE) values were calculated to quantify the contribution of individual substituents to biological activity according to previously reported methodologies [127,128,129].

#### 2.2.2. VEGFR-2 Inhibitory Activity of Compound **14**

Compound **14** demonstrated significant VEGFR-2 inhibitory activity with an IC_50_ value of 0.55 ± 0.02 µM. Although this value is lower than that of the reference inhibitor sorafenib (IC_50_ = 0.17 ± 0.01 µM), compound **14** represents a novel hybrid scaffold that achieves sub-micromolar inhibition, indicating effective target engagement (Table 4). The inhibitory profile of compound **14** suggests that it may behave as a type II-like inhibitor, where the quinoline core and the substituted thiazolidinone moiety may occupy the ATP-binding pocket and the adjacent hydrophobic regions of the VEGFR-2 kinase domain, respectively. Notably, compound **14** exhibited a strong correlation between its enzymatic inhibition and its antiproliferative activity against HepG-2 cells (IC_50_ = 7.84 ± 0.5 µM). This observation suggests that the cellular effects are likely mediated, at least partially, through disruption of the VEGF/VEGFR-2 signaling axis, a key regulator of endothelial cell-mediated angiogenesis and tumor cell proliferation.

In alignment with recent studies on multi-heterocyclic VEGFR-2 inhibitors [117], the molecular architecture of compound **14**—particularly the incorporation of a morpholine-terminated side chain—likely enhances its physicochemical properties and facilitates additional polar interactions within the kinase solvent-exposed region. Compared with previously reported scaffolds, compound **14** (IC_50_ = 0.55 µM) outperforms several heterocyclic benchmarks, such as certain thiazolidinone derivatives (IC_50_ = 0.74 µM) [130] and quinoline-based inhibitors (IC_50_ = 1.05 µM) [131]. This improvement in potency highlights the synergistic effect of combining quinoline and thiazolidinone pharmacophores, enabling improved accommodation within the VEGFR-2 binding pocket.

Collectively, these findings identify compound **14** as a promising VEGFR-2-oriented scaffold. The observed activity may warrant further investigation of its potential influence on downstream signaling events such as ERK and Akt phosphorylation to further elucidate its anti-angiogenic mechanism.

#### 2.2.3. Wound-Healing (Cell Migration) Assay of **14**

**Figure 6:**
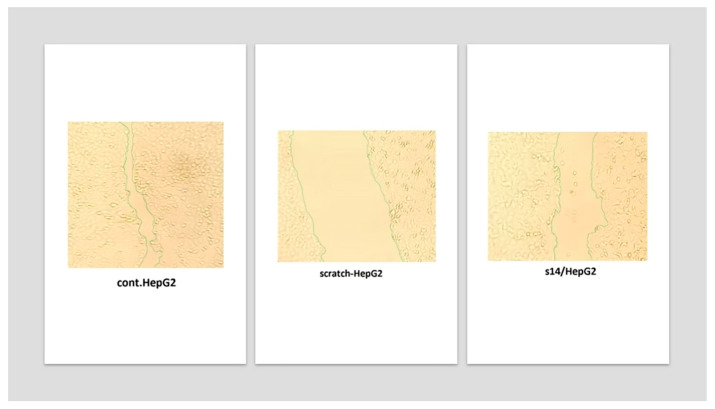
Representative wound-healing images of HepG-2 cells illustrating the effect of compound **14** on cell migration after 72 h compared to untreated control cells. Images were captured using an inverted light microscope under identical conditions.

**Figure 7:**
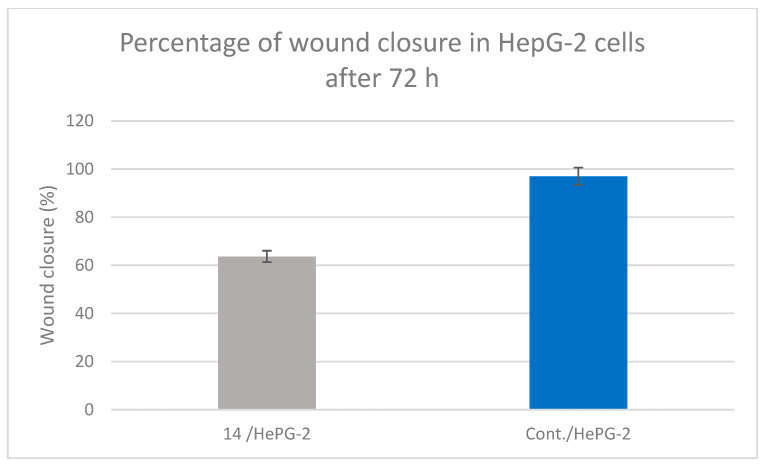
Excel-generated quantitative analysis showing the percentage of wound closure in HepG-2 cells treated with compound **14** relative to untreated control cells after 72 h.

The wound-healing assay revealed a pronounced inhibitory effect of compound **14** on HepG-2 cell migration. After 72 h, untreated control cells exhibited nearly complete wound closure (97.04 ± 3.58%), whereas treatment with compound **14** resulted in a significantly reduced wound closure of 63.70 ± 2.35% (Table 5). This marked suppression of cell migration was clearly evident in the representative wound images (Figure 6) and further confirmed by the quantitative analysis presented in Figure 7. For transparency, the raw output values obtained directly from the instrument are provided in Table S3.

The incomplete wound closure observed following treatment with compound **14** reflects an effective inhibition of the migratory capacity of HepG-2 cells rather than an experimental artifact. Importantly, this anti-migratory activity is consistent with the VEGFR-2 inhibitory activity observed for compound **14**, providing functional cellular evidence that enzymatic inhibition may translate into a measurable biological response.

Interestingly, previously reported quinoline-based anticancer agents exhibited weaker inhibition of cancer cell migration. For instance, quinoline derivatives reported by Ref. [132] showed wound closure values of approximately ~72%, while compounds described by Ref. [133] demonstrated wound closure levels of about ~70% under comparable experimental conditions. In contrast, the lower wound closure observed for compound **14** (63.70%) indicates a stronger suppression of HepG-2 cell migratory capacity.

#### 2.2.4. Cell Cycle Arrest and Apoptosis Induced by Compound **14** in HepG-2 Cells

**Figure 8:**
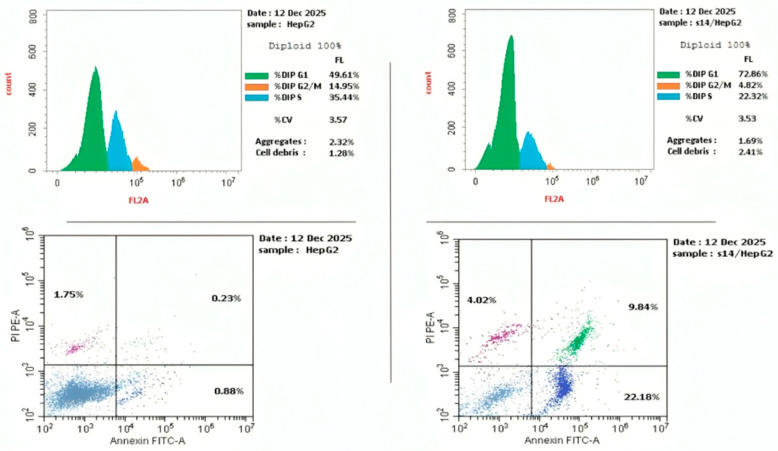
Representative flow cytometry histograms and Annexin V-FITC/PI dot plots showing the effect of compound **14** on cell cycle distribution and apoptosis in HepG-2 cells. The left panels represent untreated control cells, whereas the right panels represent HepG-2 cells treated with compound **14**.

Cell cycle analysis of HepG-2 cells treated with compound **14** revealed a pronounced disruption of cell cycle progression, characterized by significant accumulation of cells in the G_0_/G_1_ phase. The G_0_/G_1_ population increased to 72.86% compared with 49.61% in untreated control cells, indicating marked G_0_/G_1_ arrest. This was accompanied by a reduction in the S phase fraction from 35.44% to 22.32%, reflecting suppression of DNA synthesis, and a decrease in the G_2_/M phase from 14.95% to 4.82%, indicating inhibition of progression toward mitosis. These results demonstrate that compound **14** effectively halts cell cycle progression at the G_0_/G_1_ checkpoint, thereby preventing transition to DNA synthesis and mitotic phases. The detailed quantitative values are provided in Table S4, with representative flow cytometry histograms shown in Figure 8.

Consistent with the observed cell cycle arrest, apoptosis analysis demonstrated substantial induction of programmed cell death following treatment with compound **14**. Total apoptosis increased to 36.04% compared with 2.86% in control cells, corresponding to approximately a 12-fold increase. This apoptotic response was mainly driven by elevations in early apoptosis (22.18%) and late apoptosis (9.84%), while necrosis remained relatively low (4.02%), indicating that cell death occurred predominantly through a regulated apoptotic pathway rather than nonspecific cytotoxicity. The complete apoptosis dataset is summarized in Table S5, with representative dot plots shown in Figure 8.

**Figure 9:**
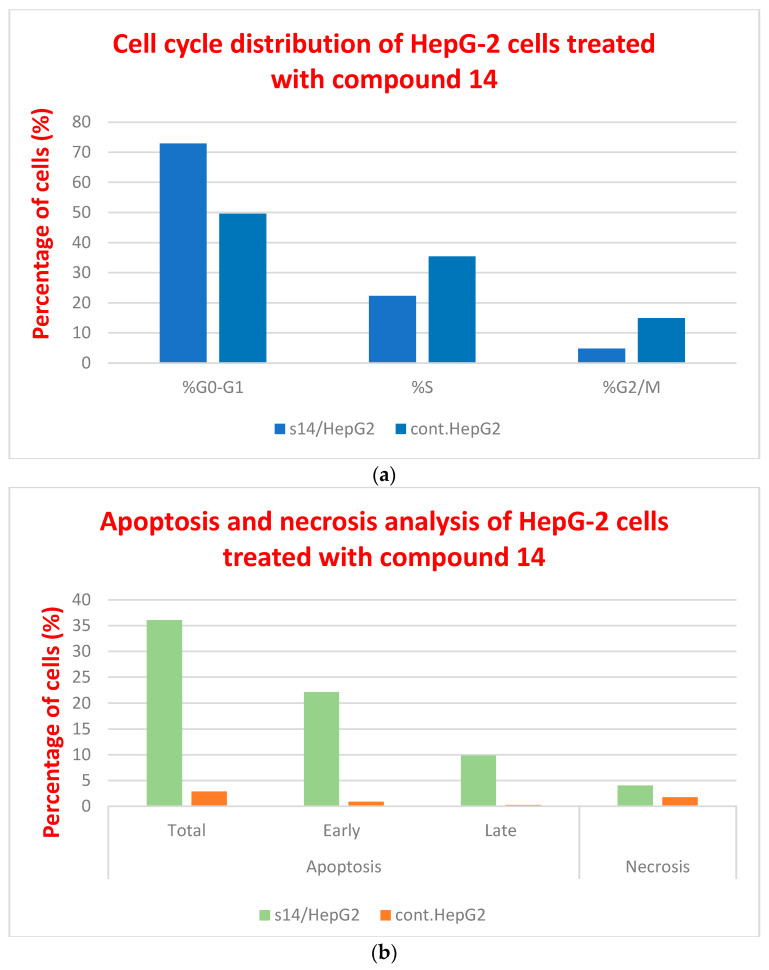
Quantitative analysis illustrating the effect of compound **14** on (**a**) cell cycle phase distribution (G_0_/G_1_, S, and G_2_/M) and (**b**) apoptosis and necrosis percentages in HepG-2 cells compared to untreated control cells.

Such induction of apoptosis together with G_0_/G_1_ cell cycle arrest is a characteristic cellular response to inhibitors of proliferative signaling pathways, including VEGFR-2, suggesting that the observed cellular effects may be associated with the VEGFR-2 inhibitory activity of compound **14**. Similar cellular responses have been reported for quinoline-based anticancer derivatives, which induced approximately ~24% total apoptosis and ~61% G_0_/G_1_ arrest in HepG-2 cells [133]. In comparison, compound **14** produced a markedly stronger biological response, with 36.04% apoptosis and 72.86% G_0_/G_1_ accumulation, highlighting its pronounced ability to suppress tumor cell proliferation and supporting its potential as a promising anticancer lead compound (Figure 9).

#### 2.2.5. Caspase-3-Mediated Apoptotic Pathway Activation Induced by Compound **14**

To further validate the apoptotic mechanism induced by compound **14**, caspase-3 activation was quantitatively assessed in HepG-2 cells using an ELISA-based assay. Treatment with compound **14** markedly increased active caspase-3 levels to 530.21 ± 20.6 pg/mL, compared with 99.69 ± 3.87 pg/mL in untreated control cells, corresponding to an approximately 5.3-fold elevation (Table 6). The reliability of the assay was confirmed by a robust calibration curve (R^2^ = 0.9772), while the raw absorbance values and instrument-generated data are provided in Tables S6 and S7.

The pronounced activation of caspase-3 indicates that cell death induced by compound **14** occurs predominantly through a caspase-dependent apoptotic pathway rather than nonspecific cytotoxicity. These findings are consistent with the Annexin V/PI apoptosis results and the observed G_0_/G_1_ cell cycle arrest, supporting the antiproliferative and pro-apoptotic effects of compound **14** in HepG-2 cells. Comparable apoptotic responses have been reported for heterocyclic anticancer agents such as 1,3,4-oxadiazole derivatives, which induce apoptosis in HepG-2 cells through activation of the intrinsic pathway involving caspase-3 and caspase-9 signaling [134]. However, the magnitude of caspase activation observed for compound **14** appears more pronounced, further supporting its strong ability to trigger programmed cell death.

Overall, the biological evaluation consistently demonstrated that compound **14** exhibits pronounced antiproliferative activity against HepG-2 cells with a favorable selectivity profile toward normal cells. Its anticancer effects were associated with VEGFR-2 inhibition, suppression of cell migration, induction of G_0_/G_1_ cell-cycle arrest, and activation of caspase-3-dependent apoptosis.

To further rationalize and support these experimental findings at the molecular level, the study was subsequently extended to comprehensive computer-aided drug discovery (CADD) investigations, including molecular docking, molecular dynamics simulations, ADMET profiling, and DFT calculations, to provide insight into target engagement, binding stability, and drug-like properties of compound **14**.

### 2.3. Computational Studies

#### 2.3.1. In Silico Elucidation of ADMET

The ADMET and physicochemical properties of compound **14** were evaluated and benchmarked against doxorubicin to assess drug-likeness and predicted safety profiles. The ADMET radar plot (Figure S48) provides an integrated overview of key parameters governing the pharmacokinetic behavior of compound **14** (MW = 539.71), indicating an overall acceptable alignment with commonly applied drug-likeness criteria, as further supported by the predicted descriptors summarized in Table S8.

Such physicochemical characteristics are consistent with the biological findings, where compound **14** exhibited pronounced antiproliferative activity and VEGFR-2 inhibition, suggesting that its balanced physicochemical profile may support adequate cellular permeability and target engagement.

Compound **14** exhibited moderate lipophilicity (LogP = 4.72), primarily influenced by the quinoline–thiophene core, which is conducive to membrane permeability and favorable π–π interactions. Concurrently, the presence of polar functionalities, including the ester group, thiazolidinone carbonyl, and morpholine moiety, contributed to a balanced hydrophilic–lipophilic profile. Consistent with this, the topological polar surface area (TPSA = 71.97 Å^2^) fell within the optimal range for orally active drug candidates, supporting sufficient polar surface exposure without adversely affecting membrane permeability.

This balanced polarity–lipophilicity profile may partially explain the observed cellular activity of compound **14**, particularly its ability to exert antiproliferative effects in HepG-2 cells.

Notably, compound **14** lacks classical hydrogen-bond donor groups (HBD = 0), while maintaining an acceptable hydrogen-bond acceptor count. This feature is frequently observed in kinase-oriented small-molecule inhibitors and does not preclude effective target engagement. In this context, binding is expected to be supported by hydrogen-bond acceptor-mediated interactions, water-bridged contacts, and hydrophobic and π–π interactions, as evidenced by the docking analysis. These predicted interaction features are consistent with the docking results that indicated stable binding of compound **14** within the VEGFR-2 active pocket, which may contribute to the experimentally observed inhibitory activity. In addition, moderate molecular flexibility imparted by the morpholinopropyl side chain is predicted to facilitate conformational adaptability and support cellular uptake and target interaction, rather than rigid high-affinity binding. Such flexibility may also assist the compound in adapting to the VEGFR-2 binding environment while maintaining the biological activity observed in the antiproliferative and enzymatic assays.

##### BOILED-EGG Model Interpretation (Absorption and Brain Penetration)

In comparison with doxorubicin, compound **14** exhibits a more favorable drug-likeness profile with respect to oral exposure-related parameters, as reflected by its substantially lower polar surface area (71.97 vs. 212.39 Å^2^) and moderate lipophilicity, which are associated with predictions of moderate human intestinal absorption. This behavior is supported by the BOILED-EGG model (Figure S49), where compound **14** is positioned within the white region, indicating a higher probability of passive gastrointestinal absorption, while remaining outside the yellow region associated with blood–brain barrier penetration.

Such a pharmacokinetic profile may contribute to the observed cellular activity of compound **14**, as sufficient gastrointestinal absorption and membrane permeability are important factors facilitating intracellular access to the molecular target.

Structurally, the quinoline–thiophene core is expected to promote membrane permeation, whereas the increased polarity contributed by heteroatoms in the morpholine ring, thiazolidinone carbonyl, and ester functionality may limit central nervous system exposure; the morpholinopropyl side chain further enhances polarity and partial ionization, favoring intestinal exposure rather than CNS distribution.

Despite these absorption-related features, compound **14** presents a complex in silico pharmacokinetic and safety profile. The predicted oral bioavailability remains very low (1.0–1.6%), primarily due to strong predicted inhibition of the major drug-metabolizing enzymes CYP2D6 and CYP3A4, in addition to very poor aqueous solubility. Moreover, while compound **14** shows a lower predicted risk of hepatotoxicity and nephrotoxicity compared with doxorubicin, potential neurotoxicity and respiratory toxicity were suggested by the prediction models. Additional toxicity screening (Table S9) predicts compound **14** to be non-tumorigenic, non-irritant, and safe with respect to reproductive toxicity; however, a mutagenicity alert was identified at the predictive level. Furthermore, the relatively low composite drug score (0.16), compared with doxorubicin (0.33), indicates that although compound **14** represents a promising scaffold with favorable absorption distribution tendencies, further medicinal chemistry optimization will be required to improve solubility, mitigate metabolic liabilities, and address the predicted safety concerns.

This balance may support both cellular uptake and effective interaction with the VEGFR-2 binding pocket, which is consistent with the experimentally observed VEGFR-2 inhibitory activity and the antiproliferative effects against HepG-2 cells.

#### 2.3.2. Molecular Docking Study

The molecular docking study was conducted to investigate the binding mode of compound **14** within the active site of VEGFR-2 and to compare its interaction profile with that of the well-established VEGFR-2 inhibitor sorafenib (PDB ID: 4ASD). Considering the well-established role of the VEGF/VEGFR-2 signaling pathway in tumor angiogenesis, the docking analysis aimed to provide a mechanistic interpretation of ligand–receptor interactions beyond simple docking scores. The list of protein structures used in the docking simulations, including their PDB identifiers, crystal structure resolutions, active-site coordinates, and reference ligands, is provided in the Supplementary Materials (Table S10).

**Figure 10:**
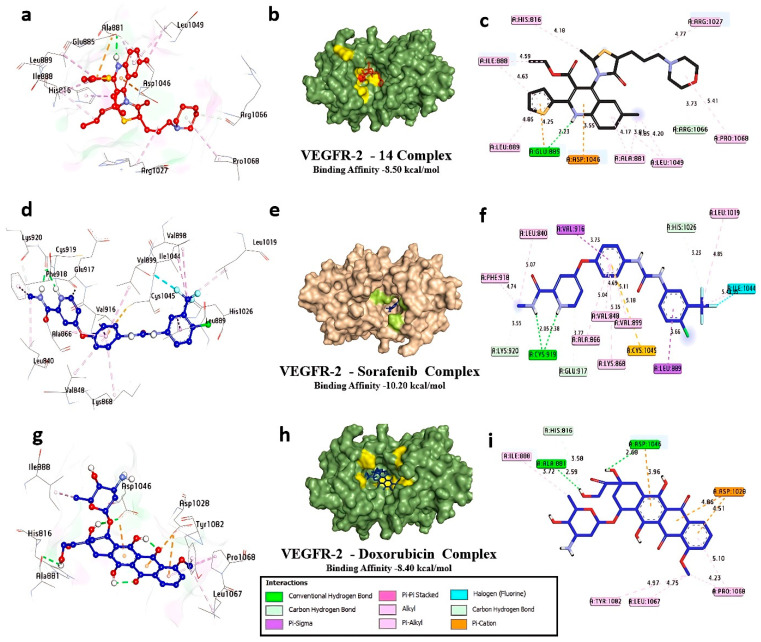
Molecular docking analysis of compound **14**, sorafenib, and doxorubicin within the VEGFR-2 active site. (**a**) Two-dimensional interaction map of compound **14** showing hydrogen bonding, hydrophobic, and π-mediated interactions with key amino acid residues. (**b**) Three-dimensional surface representation of the VEGFR-2–compound **14** complex illustrating the accommodation of the ligand within the ATP-binding pocket and its predicted binding affinity. (**c**) Two-dimensional ligand–protein interaction diagram of compound **14** highlighting the interacting residues and interaction distances. (**d**) Two-dimensional interaction map of sorafenib within the VEGFR-2 active site showing hydrogen-bonding and hydrophobic contacts. (**e**) Three-dimensional surface representation of the VEGFR-2–sorafenib complex within the ATP-binding pocket (PDB ID: 4ASD). (**f**) Two-dimensional ligand–protein interaction diagram of sorafenib illustrating the main binding interactions and distances. (**g**) Two-dimensional interaction map of doxorubicin within the VEGFR-2 active site. (**h**) Three-dimensional surface representation of the VEGFR-2–doxorubicin complex within the binding pocket. (**i**) Two-dimensional ligand–protein interaction diagram of doxorubicin highlighting the key interactions and distances. Different interaction types are represented by color-coded dashed lines as indicated in the legend (e.g., hydrogen bonds, hydrophobic interactions, π–π stacking, and π–cation interactions).

From a medicinal chemistry perspective, the binding orientation of compound **14** is strongly supported by its hybrid structural architecture, which enables efficient accommodation within the VEGFR-2 ATP-binding cavity (Figure 10a–c). The quinoline core, together with the thiophene ring, forms a hydrophobic scaffold projecting toward the interior of the binding pocket and facilitating π–alkyl interactions with key residues **Ile888**, **Ala881**, and **Leu889**. In addition, the morpholine-terminated side chain acts as a flexible modification vector extending toward the outer solvent-exposed region of the binding site, interacting with residues **Arg1066** and **Pro1068** (Figure 10c). This orientation is consistent with previous reports indicating that morpholine-containing scaffolds can promote favorable peripheral polar interactions while maintaining stable engagement within the kinase catalytic pocket [117]. The outward-directed side chain also offers a potential vector for further structural optimization, allowing additional substituents to extend toward the solvent-exposed region without disrupting key catalytic interactions. Notably, compound **14** forms a hydrogen bond with Glu885 (≈2.23 Å), which contributes to stabilization of the ligand within the active site.

Docking analysis revealed that compound **14** adopts a favorable binding pose within the VEGFR-2 catalytic pocket with a docking score of −8.50 kcal/mol. In addition to the hydrogen bond with Glu885, several stabilizing interactions were observed, including π–alkyl contacts with **Pro1068**, **Leu1049**, **Ala881**, **Leu889**, **Ile888**, and **His816**. Importantly, compound **14** also established π–cation interactions with **Asp1046**, a member of the conserved DFG motif, as well as with **Asp1028**, further supporting effective target engagement consistent with its sub-micromolar enzymatic inhibition. A carbon–hydrogen bond with **Arg1066** additionally contributes to stabilization of the ligand within the ATP-binding region (Table S11).

For comparison, sorafenib displayed the expected interaction profile within the VEGFR-2 binding pocket (Figure 10d–f) with a docking score of −10.20 kcal/mol. The ligand formed two hydrogen bonds with the hinge residue **Cys919**, along with multiple hydrophobic interactions including π–alkyl contacts with **Phe918, Leu840**, **Ala866**, **Val848**, **Val899**, and **Lys868**, as well as a sulfur bond with **Cys1045** and π–sigma interactions with **Leu889** and **Val916**. These interactions reproduce the experimentally resolved binding mode of sorafenib and highlight the importance of hinge-region hydrogen bonding for VEGFR-2 inhibition.

Although compound **14** exhibited a slightly lower docking score than sorafenib, it established a robust network of interactions compatible with stable binding within the catalytic pocket. Despite the absence of classical hydrogen-bond donor groups (HBD = 0), compound **14** maintained a favorable binding pose through a combination of hydrogen-bond acceptor interactions and extensive π-mediated contacts, thereby optimizing van der Waals complementarity within the binding cavity. For completeness, doxorubicin was also included as a comparative ligand (Figure 10g–i). However, its interaction pattern differed markedly from those of sorafenib and compound **14**, consistent with its well-known antiproliferative mechanism involving DNA intercalation rather than kinase inhibition. To further contextualize the docking results, several well-known VEGFR-2 inhibitors reported in crystallographic databases and the literature were considered as reference ligands. A benchmarking set of experimentally validated VEGFR-2 inhibitors was therefore used as positive controls, and their reported binding affinities and docking scores are summarized in Table S12. This comparison provides a qualitative reference framework for interpreting the docking score obtained for compound **14**.

Overall, the docking analysis suggests that compound **14** effectively occupies the VEGFR-2 ATP-binding pocket through a combination of hydrogen-bond acceptor interactions and hydrophobic contacts, supporting its experimentally observed antiproliferative activity against HepG-2 cells. It should be emphasized that molecular docking involves several approximations and does not directly provide the absolute binding free energy (ΔG). Theoretically, the binding free energy depends on the difference between the free energies of the complexed and unbound states of the protein and ligand in solution: ΔG_binding_ = G_complex_ − (G_protein_ + G_ligand_)

Because docking scoring functions simplify these thermodynamic contributions, the resulting scores should be interpreted qualitatively. Accordingly, molecular docking was primarily employed to rationalize plausible binding modes and rank ligands according to predicted affinity rather than to determine absolute binding free energies. To further evaluate the reliability of the docking protocol and to address potential false positives in docking-based screening, an additional validation analysis was conducted using both positive and negative control datasets. The benchmarking set of experimentally validated VEGFR-2 inhibitors served as positive controls (Table S12). In addition, two categories of negative controls were included: property-matched decoy molecules obtained from the DUD-E database (Table S13) and structurally unrelated compounds lacking reported kinase activity (Table S14). Comparative statistical analysis demonstrated clear separation between binders and non-binders (Table S15), indicating reasonable discriminatory power of the docking workflow.

Finally, to explore the broader binding potential of compound **14**, an additional theoretical docking study was performed against several viral enzymes, including HIV-1 protease, hepatitis virus polymerase, and SARS-CoV-2 main protease (Mpro). The predicted interaction profiles and calculated binding affinities are summarized in Table S16, while representative docking poses are shown in Figure S50. These results provide a comparative overview of the potential binding modes of compound **14** with different viral enzymes at the molecular level.

#### 2.3.3. Molecular Dynamics Simulations

**Figure 11:**
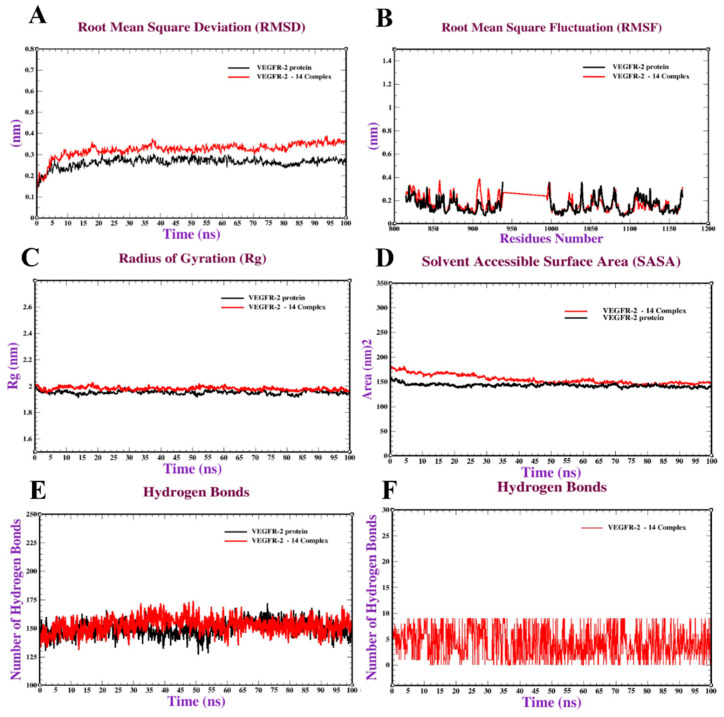
Molecular dynamics simulation analysis of VEGFR-2 in the free form and in complex with compound **14** over 100 ns. (**A**) Root mean square deviation (RMSD) illustrating the overall structural stability of VEGFR-2 and the VEGFR-2-**14** complex. (**B**) Root mean square fluctuation (RMSF) showing residue-level flexibility and localized conformational fluctuations upon ligand binding. (**C**) Radius of gyration (Rg) depicting the compactness and folding stability of the protein systems. (**D**) Solvent-accessible surface area (SASA) highlighting changes in solvent exposure upon complex formation. (**E**) Intramolecular hydrogen bonds reflecting internal protein stability. (**F**) Intermolecular hydrogen bonds between VEGFR-2 and compound **14**, indicating the persistence and stability of protein–ligand interactions.

To gain deeper insight into the dynamic stability and binding behavior of compound **14** within the VEGFR-2 active site, a 100 ns molecular dynamics (MD) simulation was performed for both the free protein and the VEGFR-2-**14** complex. The structural stability of the systems was first evaluated using root mean square deviation (RMSD) analysis (Figure 11A). Both systems reached equilibrium after approximately 40 ns, with RMSD values remaining below 0.35 nm throughout the simulation, indicating overall structural stability within the simulated time frame. Notably, the VEGFR-2-**14** complex exhibited slightly higher RMSD values than the free protein, reflecting minor conformational adjustments upon ligand binding rather than structural destabilization.

Such controlled conformational adaptation is commonly observed for kinase–ligand complexes and supports the ability of compound **14** to remain accommodated within the VEGFR-2 catalytic pocket during the simulation.

Residue-level flexibility was assessed through root mean square fluctuation (RMSF) analysis (Figure 11B). The VEGFR-2-**14** complex showed moderately increased fluctuations in specific regions, particularly within loop segments surrounding the binding site, while the majority of residues maintained RMSF values below 0.5 nm. This localized flexibility suggests adaptive movements that facilitate ligand accommodation without compromising the global stability of the protein structure.

These dynamic adjustments may help stabilize ligand positioning while maintaining key interactions predicted in the docking study.

The compactness of VEGFR-2 upon ligand binding was further examined by monitoring the radius of gyration (Rg) (Figure 11C). The VEGFR-2-**14** complex displayed relatively stable Rg values over the simulation time, indicating preservation of a compact folded state. Compared with the free protein, the complex maintained slightly lower and more compatible with Rg values, suggesting that binding of compound **14** promotes structural compactness and conformational stability of VEGFR-2 on the nanosecond time scale.

Maintenance of this compact structure supports the formation of a stable protein–ligand complex under simulated physiological conditions.

Solvent-accessible surface area (SASA) analysis (Figure 11D) revealed a noticeable reduction in solvent exposure upon ligand binding. The VEGFR-2-**14** complex consistently exhibited lower SASA values than the free protein, implying burial of hydrophobic regions and reduced solvent accessibility. This behavior supports the formation of a tightly packed protein ligand complex and contributes to its enhanced short-term stability.

Such burial of hydrophobic regions is consistent with the hydrophobic and π-mediated contacts identified in the docking analysis.

Hydrogen bond analysis provided additional insights into interaction persistence and structural integrity. The number of intramolecular hydrogen bonds within VEGFR-2 increased upon complex formation (Figure 11E), indicating improved internal stabilization of the protein framework. Furthermore, analysis of intermolecular hydrogen bonds between VEGFR-2 and compound **14** (Figure 11F) showed the formation of up to eight hydrogen bonds with moderate fluctuations over the simulation period. The persistence of these interactions highlights a stable yet dynamic binding mode, allowing the ligand to maintain productive contacts while adapting to local conformational changes during the simulated trajectory.

These persistent interactions further support the experimentally observed VEGFR-2 inhibitory activity of compound **14**.

Collectively, the MD simulation results demonstrate that compound **14** forms a dynamically stable complex with VEGFR-2 within the simulated nanosecond time scale. The balanced combination of structural stability, localized flexibility, reduced solvent exposure, and sustained hydrogen bonding supports a favorable short-term binding mode. Importantly, this dynamic stability is similar with the experimentally observed VEGFR-2 inhibition and the antiproliferative activity of compound **14** against HepG-2 cells, suggesting that the predicted binding interactions may contribute to its biological activity. These findings reinforce the docking results and provide supportive dynamic insight into the binding behavior of compound **14**. However, it is important to acknowledge the inherent limitations of nanosecond-scale MD simulations. While compound **14** remained stable during the 100 ns trajectory, such simulations may not capture long-timescale ligand dissociation events or kinetic parameters such as $k_{off}$, which often occur on millisecond-to-second timescales. Therefore, the observed stability within 100 ns should be interpreted as indicative of short-term complex stability rather than definitive evidence of long-term binding residence.

#### 2.3.4. Quantum Chemical Calculations

Density functional theory (DFT) calculations were performed to investigate the electronic properties and global reactivity descriptors of compounds **1**–**14**. The graphical representations of the HOMO and LUMO orbitals are shown in Table S17, while the calculated orbital energies and derived quantum chemical descriptors are summarized in Table 7.

The HOMO energies of the investigated compounds ranged from −0.164 to −0.262 eV, indicating variable electron-donating abilities across the series. Compounds **14** (−0.164 eV) and **13** (−0.187 eV) exhibited the highest HOMO energies, suggesting stronger electron-donating character, whereas compounds such as **1** (−0.262 eV) showed weaker donation. In parallel, the LUMO energies varied between −0.139 and −0.226 eV, with compound **14** (−0.226 eV) displaying the most stabilized LUMO, indicative of superior electron-accepting ability.

Most compounds exhibited moderate HOMO–LUMO energy gaps (ΔE ≈ 0.04–0.11 eV), reflecting a balance between electronic stability and reactivity. Notably, compound **14** showed the smallest energy gap (ΔE = 0.01023 eV), followed by compound **13** (ΔE = 0.02814 eV) and compound **10** (ΔE = 0.03556 eV), highlighting their enhanced electronic polarizability and increased chemical softness relative to the remaining derivatives.

This trend was further supported by the calculated chemical hardness (η) and softness (σ) values. Compound **14** exhibited the lowest hardness (η = 0.0051 eV) and the highest softness (σ = 6.27 eV^−1^), confirming its highly flexible electronic structure. In comparison, compounds **13** (σ = 2.96 eV^−1^) and 10 (σ = 1.86 eV^−1^) showed moderate softness, while the rest of the series displayed higher hardness values.

Electronegativity (χ) values were relatively similar across the series (χ ≈ 0.19–0.24 eV), suggesting comparable global electronic character. However, pronounced differences were observed in the electrophilicity index (ω). Compound **14** exhibited an exceptionally high electrophilicity index (ω = 195.50 eV), markedly exceeding those of compound **13** (ω = 71.07 eV) and compound **10** (ω = 56.24 eV), indicating a strong electrophilic nature. Similarly, compound **14** showed the highest maximum charge transfer value (ΔN_max_ = 17.5), compared with 7.26 for compound **13** and 5.12 for compound **10**, reflecting superior charge-accepting capability.

Overall, the quantitative DFT results summarized in Table 6 identify compound **14** as the most electronically reactive and flexible member of the series, followed by compounds **13** and **10**, while the remaining derivatives exhibit moderate to weak electronic reactivity.

##### Electrostatic Potential (ESP) Maps

**Figure 12:**
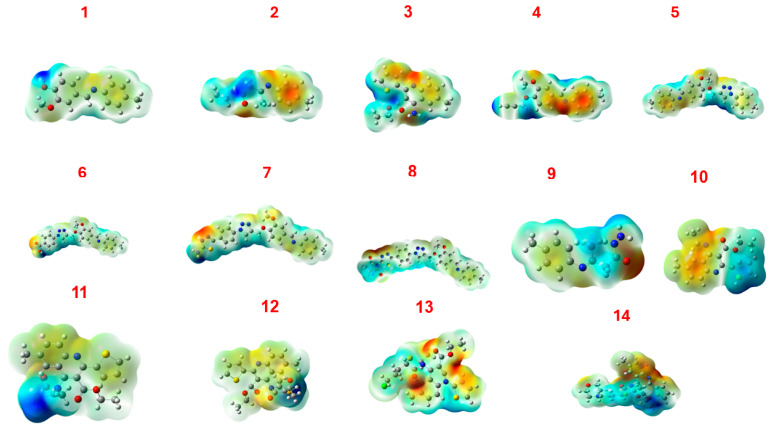
Molecular electrostatic potential (ESP) surfaces of compounds **1**–**14** mapped onto the electron density isosurfaces, illustrating the distribution of electron-rich (blue) and electron-deficient (yellow–red) regions and highlighting the influence of different functional groups on the electrostatic profiles across the series.

The ESP surfaces of compounds **1**–**14** (Figure 12) demonstrate how the progressive introduction of different functional groups governs the electrostatic potential distribution across the series. In the early derivative compound **1**, the ESP profile is characterized by an extended electron-rich region localized on the oxygenated aromatic ring, arising from the combined electron-donating effects of the phenolic –OH and methoxy –OCH_3_ groups, while the imine linkage appears as a positively polarized region separating the two aromatic moieties. In compound **2**, the ESP distribution is dominated by the ester carbonyl group, which generates a localized electron-rich region, whereas the unsubstituted aromatic rings remain relatively electron-deficient. Introduction of an amino group in compound **3** leads to additional electron density around the –NH_2_ functionality, resulting in a broader electron-rich region.

In compound **4**, incorporation of a propargyl ether substituent introduces an electron-rich region localized on the ether oxygen, while the C≡C moiety exhibits a neutral to slightly positive electrostatic potential, in agreement with its sp-hybridized nature. The introduction of a triazole ring in compound **5** produces a pronounced electron-rich domain centered on the nitrogen-rich heterocycle. Replacement of the terminal methyl group by a carboxylic acid in compound **6** markedly enhances the negative electrostatic potential around the –COOH oxygen atoms. Formation of an amino-thiadiazole ring in compound **7** leads to an intense electron-rich region associated with the heterocyclic nitrogen atoms and the exocyclic –NH_2_ group. In compound **8**, the pyrrolidine ring contributes additional electron density through the ring nitrogen and adjacent carbonyl groups, yielding a more heterogeneous ESP surface. Introduction of a hydrazide moiety in compound **9** generates an extended electron-rich region localized on the carbonyl oxygen and terminal nitrogen atoms. In compound **10**, conversion of the amino functionality into an imine group results in a positively polarized region along the C=N bond, reflecting increased electron deficiency. Cyclization to form a 2-methyl-3-thiazolidin-4-one ring in compound **11** introduces a strong electron-rich region centered on the thiazolidinone carbonyl oxygen. Subsequent acylation in compound **12** further intensifies this effect through the introduction of an additional carbonyl group. In compound **13**, attachment of a –CH_2_CH_2_Cl substituent contributes localized electrostatic modulation around the chlorine atom without altering the dominant influence of the carbonyl and heterocyclic functionalities.

Finally, compound **14** exhibits the most diversified ESP distribution, where incorporation of a morpholine ring generates an additional electron-rich region localized on the morpholine oxygen and nitrogen atoms, leading to a well-balanced electrostatic surface.

For completeness, the corresponding electron density surfaces are provided in the Table S18.

##### Integrated DFT of the Most Potent Compound (**14**)

The DOS spectrum of compound **14** reveals that the aromatic-imine framework and carbonyl-containing groups dominate the occupied electronic states, while the morpholine ring introduces low-lying virtual orbitals close to the frontier region through the lone pairs of its oxygen and nitrogen atoms. This complementary contribution of conjugated aromatic systems and heteroatom-rich functionalities results in a dense and continuous DOS profile, indicative of enhanced electronic responsiveness and facile charge redistribution.

Further insight is provided by the ELF map of compound **14**, where regions with high ELF values (0.8–1.0, red–orange) are clearly localized around the carbonyl oxygen atoms of both the ester (–COOEt) and thiazolidin-4-one groups, as well as the morpholine oxygen, identifying these functionalities as the primary electron-rich sites with strongly localized lone-pair electrons. In contrast, areas displaying moderate ELF values (0.5–0.7, yellow–green) extend over the quinoline and thiophene rings, reflecting π-electron delocalization within the aromatic framework and providing an electronically stable backbone. Regions with lower ELF values (0.2–0.4, cyan–blue) are mainly associated with the imine (–C=N–) linkage and heteroatom-containing bonds, indicating polarized interactions that facilitate intramolecular charge transfer, while very low ELF values (≤0.1, dark blue) appear along the alkyl linker, comparable with its primarily structural role.

**Figure 13:**
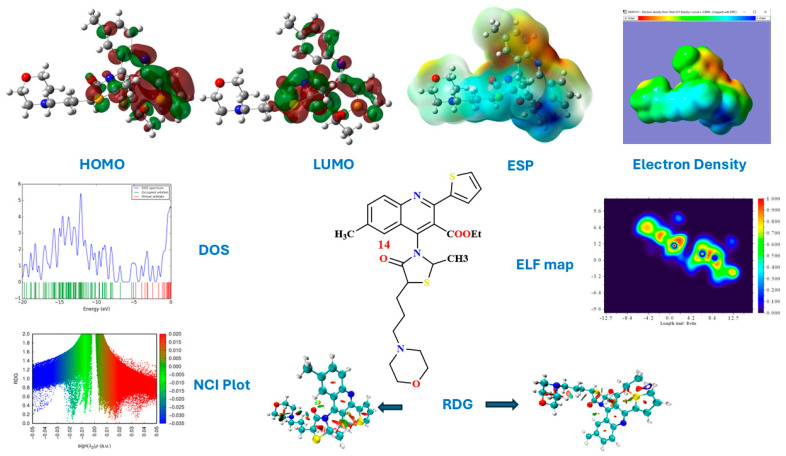
HOMO-LUMO distributions, DOS spectrum, electrostatic potential (ESP) surface, electron density, ELF map, and non-covalent interaction (NCI) analysis (RDG plot and 3D isosurfaces) of compound **14**, illustrating its electronic structure, charge distribution, and non-covalent interaction profile underlying its enhanced biological activity.

The non-covalent interaction (NCI) analysis, based on the RDG vs. sign(λ_2_)ρ plots, further supports this electronic picture. Strong attractive interactions are observed at negative sign(λ_2_)ρ values (−0.035 to −0.01 a.u.), localized around the carbonyl and morpholine oxygen atoms, confirming their involvement in hydrogen-bonding and electrostatic stabilization. Weak dispersive interactions appear near sign(λ_2_)ρ ≈ −0.01 to +0.01 a.u., predominantly over the aromatic and thiophene frameworks, contributing to conformational stability, while localized steric repulsion at positive sign (λ_2_)ρ values arises from crowded regions within the fused heterocyclic core, particularly around the imine linkage. Consistently, the 3D NCI isosurfaces visualize strong attractive regions (blue) near carbonyl and morpholine heteroatoms, weak dispersive regions (green) over the aromatic backbone and linker, and limited steric repulsion (red) at heterocyclic junctions. Collectively, the integrated DOS, ELF, and NCI analyses demonstrate that compound **14** combines highly localized electron-rich centers, delocalized aromatic domains, and balanced non-covalent interactions, resulting in an optimized and adaptable electronic architecture. This synergy provides a clear physicochemical rationale for the superior anticancer activity of compound **14** compared to the other members of the series (Figure 13).

## 3. Materials and Methods

### 3.1. Chemistry

All apparatus data used to elucidate the chemical structure of new compounds are attached in the Supplementary Materials. Representative IR, NMR, and Mass charts are attached in the Supplementary Materials (Figures S1–S46).

**(*E*)-2-Methoxy-4-[(*p*-tolylimino)methyl]phenol (1)**

A solution of *p*-toluidine (10 mmol, 1.07 g) and Vanillin (10 mmol, 1.52 g) in ethanol (10 mL) was prepared in the presence of a catalytic amount of acetic acid. The reaction mixture was heated under reflux at 120–150 °C for 6 h. The progress of the reaction was monitored by TLC using benzene: acetone (5:1) as the eluent. After completion, the reaction mixture was allowed to cool to room temperature and subsequently poured into 50 mL of chilled water, which led to the formation of a precipitate. The resulting solid was collected by vacuum filtration and thoroughly washed with cold ethanol. Recrystallization from ethylacetate afforded compound **1** as a yellowish powder. Yield: 89%; m.p. 156–158 °C; IR (KBr) cm^−1^, *ν*: 3421 (O–H), 3064, 3010 (Ar–CH), 2926, 2858 (Aliphatic CH), 1661 (C=N), 1600, 1511 (Ar C=C), 1273 (C–OCH_3_). ^1^H NMR (DMSO-*d*_6_, 400 MHz) δ: 2.35 (s, 3H, CH_3_), 3.85 (s, 3H, OCH_3_), 7.06–7.47 (m, 7H, Ar–H), 8.86 (s, 1H, CH=N), 9.50 (brs, 1H, OH) ppm. ^13^C NMR (DMSO-*d*_6_, 100 MHz) δ: 21.08 (CH_3_), 59.33 (OCH_3_), 115.71–139.58 (9Ar-C), 147.12 (Ar-C-N), 149.13 (Ar-C-OCH_3_), 151.78 (Ar-C-OH), 159.75 (C=N) ppm. Analysis calcd. for C_15_H_15_NO_2_ (241.11): C, 74.67; H, 6.27; N, 5.81. Found: Elemental Analysis: C, 75.01; H, 6.29; N, 5.80%.

**Ethyl (*E*)-3-(*p*-tolylimino)butanoate (2)**

In situ deprotonation of *p*-toluidine (10 mmol, 1.07 g) was achieved by treatment with KOH (0.56 mL, 10 mmol) in methanol (10 mL) under continuous stirring at ambient temperature for 30 min, generating the corresponding activated nucleophilic species. Ethyl acetoacetate (1.3 mL, 10 mmol) was then introduced dropwise, allowing nucleophilic addition and subsequent condensation to proceed smoothly at room temperature for 10–12 h. Reaction progress was monitored by TLC using benzene:acetone (5:1) as the elution system until complete consumption of the starting material was observed. The reaction mixture was left to stand for 30 min, promoting gradual precipitation of the desired product. The formed solid was isolated by vacuum filtration, washed thoroughly with cold ethanol, and purified by recrystallization from hexane to furnish compound **2** as an orange powder. Yield: 92%; m.p. 200–202 °C. IR (KBr) cm^−1^, *ν*: 3028 (Ar–CH), 2982, 2919, 2864 (aliphatic CH), 1747 (C=O), 1637 (C=N), 1602 (Ar C=C). ^1^H NMR (DMSO-*d*_6_, 400 MHz) δ: 1.36 (t, 3H, *J* = 7.7 Hz, CH_3_ ester), 1.78 (t, 3H, *J* = 7.6 Hz, CH_3_–C=N), 2.33 (s, 3H, CH_3_–Ph), 3.06 (s, 2H, CH_2_), 4.51 (q, 2H, *J* = 5.4 Hz, CH_2_ ester), 7.26–7.47 (m, 4H, Ar–H) ppm. ^13^C NMR (DMSO-*d*_6_, 100 MHz) δ: 12.12 (CH_3_ ester), 20.31 (CH_3_–C=N), 21.06 (CH_3_–Ph), 55.19 (CH_2_–C=O), 63.07 (CH_2_ ester), 114.31–135.05 (5Ar-C), 145.04 (Ar-C–N), 163.02 (C=N), 166.05 (C=O) ppm. Analysis calcd. for C_13_H_17_NO_2_ (219.13): C, 71.21; H, 7.81; N, 6.39. Found: Elemental Analysis: C, 71.20; H, 7.84; N, 6.39%.

**Ethyl 4-amino-6-methyl-2-(thiophen-2-yl)quinoline-3-carboxylate (3)**

The pre-synthesized arylidene intermediate, ethyl (*E*)-2-cyano-3-(thiophene-2-yl)acrylate (2.07 g, 10 mmol), was reacted with *p*-toluidine (10 mmol, 1.07 g) in ethanol (10 mL). Triethylamine (1.0 mL, 10 mmol) was added dropwise, and the reaction mixture was refluxed at 120–150 °C for 8 h. The reaction progress was monitored by TLC using benzene:acetone (5:1) as the eluent. After completion, the mixture was cooled and poured into chilled water (50 mL), affording a precipitate. The solid was filtered under vacuum, washed with cold ethanol, and recrystallized from benzene/chloroform to yield compound **3** as a pale brown powder. Yield: 75%; m.p. 247–249 °C. IR (KBr) cm^−1^, *ν*: 3425, 3415 (NH_2_), 3084, 3024 (Ar–CH), 2975, 2912 (aliphatic CH), 1718 (C=O), 1597 (C=N), 1502 (Ar C=C). ^1^H NMR (DMSO-*d*_6_, 400 MHz) δ: 1.03 (t, 3H, *J* = 5.9 Hz, CH_3_–CH_2_), 2.30 (s, 3H, CH_3_–Ph), 4.36 (q, *J* = 6.4 Hz, 2H, CH_2_), 6.02 (brs, 2H, NH_2_), 7.16–7.52 (m, 6H, Ar–H) ppm. ^13^C NMR (DMSO-*d*_6_, 100 MHz) δ: 14.92 (CH_3_–CH_2_), 23.06 (CH_3_–Ph), 59.80 (CH_2_), 103.97 (C pyridine–CO), 128.74–131.39 (9Ar-C), 140.57 (C pyridine–N), 156.53 (C=N pyridine), 158.00 (C pyridine–NH_2_), 166.23 (C=O) ppm. Analysis calcd. for C_17_H_16_N_2_O_2_S (312.09): C, 65.36; H, 5.16; N, 8.97. Found: C, 65.35; H, 5.16; N, 8.96%.

**(*E*)-1-[3-Methoxy-4-(prop-2-yn-1-yloxy)phenyl]-*N*-(p-tolyl)methanimine (4)**

Compound **1** (10 mmol, 2.41 g), was dissolved in dry acetone (10 mL) and stirred at ambient temperature for 10 min. Anhydrous potassium carbonate (10 mmol, 1.38 g) was then added, and stirring was continued for an additional 30 min. Propargyl bromide was subsequently introduced dropwise under cooling conditions, and the reaction mixture was stirred at 40–60 °C for 4 h. Reaction progress was monitored by TLC using benzene:acetone (5:1) as the eluent. After completion, the mixture was allowed to cool to room temperature and poured onto ice, resulting in the formation of a precipitate. The solid was collected by vacuum filtration, thoroughly washed with cold ethanol, and recrystallized from ethanol to afford compound **4** as a yellow powder. Yield: 75%; m.p. 175–177 °C. IR (KBr) cm^−1^, *ν*: 3304 (≡C–H), 3065, 3010 (Ar–CH), 2935, 2869, 2735 (aliphatic CH), 2254 (C≡C), 1675 (C=N), 1593, 1432 (Ar C=C). ^1^H NMR (DMSO-*d*_6_, 400 MHz) δ: 2.37 (s, 3H, CH_3_), 3.70 (s, 1H, C≡CH), 3.87 (s, 3H, OCH_3_), 4.22 (s, 2H, CH_2_), 7.26–7.47 (m, 7H, Ar–H), 8.50 (s, 1H, CH=N) ppm. ^13^C NMR (DMSO-*d*_6_, 100 MHz) δ: 21.12 (CH_3_), 59.07 (OCH_3_), 63.00 (CH_2_), 75.19 (C≡CH), 79.13 (C≡C), 114.31–135.05 (9Ar-C), 145.04 (Ar-C–N), 151.00 (Ar-C–OCH_3_), 154.05 (Ar-C–OH), 159.02 (C=N) ppm. Analysis calcd. for C_18_H_17_NO_2_ (279.13): C, 77.40; H, 6.13; N, 5.01. Found: C, 77.38; H, 6.15; N, 5.00%.

**(*E*)-1-{3-Methoxy-4-[(1-(*p*-tolyl)-1*H*-1,2,3-triazol-4-yl)methoxy]phenyl}-*N*-(*p*-tolyl)methanimine (5)**

Compound **4** (10 mmol, 2.79 g) and 1-azido-4-methylbenzene (10 mmol, 133.15) were dissolved in THF/H_2_O (10 mL), followed by the addition of CuSO_4_·5H_2_O (10 mmol, 2.50 g) and sodium ascorbate (10 mmol, 1.98 g). The reaction mixture was refluxed at 80 °C for 9 h, and its progress was monitored by TLC using benzene:acetone (5:1) as the eluent until completion. After cooling to room temperature, the mixture was treated with activated charcoal and stirred for 30 min, leading to the formation of a precipitate. The solid was isolated by vacuum filtration, thoroughly washed with cold water, and recrystallized from ethanol to yield compound **5** as a dark brown powder. Yield: 65%; m.p. 198–200 °C. IR (KBr) cm^−1^, *ν*: 3090, 3030 (Ar–CH), 2966, 2926 (aliphatic CH), 1633 (C=N), 1568, 1503 (Ar C=C), 1398 (N=N). ^1^H NMR (DMSO-*d*_6_, 400 MHz) δ: 2.37, 2.45 (s, 6H, 2CH_3_), 3.85 (s, 3H, OCH_3_), 5.25 (s, 2H, CH_2_), 7.06–7.97 (m, 11H, Ar–H), 8.02 (s, 1H, CH triazole), 8.50 (s, 1H, CH=N) ppm. ^13^C NMR (DMSO-*d*_6_, 100 MHz) δ: 20.01, 21.09 (2CH_3_), 59.11 (OCH_3_), 73.00 (CH_2_), 115.24 (CH triazole), 115.71–135.21 (17Ar-C), 139.83 (C triazole), 150.20 (Ar-C–OCH_3_), 160.03 (C=N) ppm. EI-MS: *m*/*z* = 412.53 (M^+^), with a base peak at *m*/*z* 134. Anal. calcd. for C_25_H_24_N_4_O_2_ (412.19): C, 72.80; H, 5.86; N, 13.58%. Found: C, 72.79; H, 5.88; N, 13.55%.

**(*E*)-4-{{{4-{{{2-Methoxy-4-[(*p*-tolylimino)methyl]phenoxy}methyl}}-1*H*-1,2,3-triazol-1-yl}}}benzoic acid (6)**

Compound **5** (10 mmol, 4.12 g) was dissolved in an aqueous NaOH solution (3 g in 30 mL H_2_O), followed by the addition of KMnO_4_ (5 mmol, 0.79 g). The reaction mixture was refluxed at 90 °C for 2–4 h, and its progress was monitored by TLC using benzene:acetone (5:1) as the eluent until completion. After cooling to room temperature, the reaction mixture was poured into chilled water (50 mL), resulting in the formation of a precipitate. The solid was isolated by vacuum filtration, thoroughly washed with cold ethanol, and recrystallized from ethanol to afford compound **6** as an orange powder. Yield: 60%; m.p. 222–224 °C. IR (KBr) cm^−1^, *ν*: 3481 (O–H), 3096, 3030 (Ar–CH), 2966, 2926 (aliphatic CH), 1721 (C=O), 1625 (C=N), 1516, 1431 (Ar C=C), 1398 (N=N). ^1^H NMR (DMSO-*d*_6_, 400 MHz) δ: 2.38 (s, 3H, CH_3_), 3.87 (s, 3H, OCH_3_), 5.20 (s, 2H, CH_2_), 7.06–7.97 (m, 9H, Ar–H), 8.02 (s, 1H, CH triazole), 8.25 (d, 2H, *J* = 8.7 Hz Ar–H), 8.50 (s, 1H, CH=N), 12.12 (brs, 1H, COOH) ppm. ^13^C NMR (DMSO-*d*_6_, 100 MHz) δ: 20.57 (CH_3_), 59.07 (OCH_3_), 73.27 (CH_2_), 115.84 (CH triazole), 116.23–139.37 (16Ar-C), 139.72 (C triazole), 152.11 (Ar-C–OCH_3_), 152.53 (Ar-C–OCH_2_), 159.64 (C=N), 160.03 (COOH) ppm. Analysis calcd. for C_25_H_22_N_4_O_4_ (442.16): C, 67.86; H, 5.01; N, 12.66. Found: C, 67.85; H, 5.04; N, 12.66%.

**(*E*)-5-{{{{4-{{{4-{{{2-Methoxy-4-[(*p*-tolylimino)methyl]phenoxy}methyl}}-1*H*-1,2,3-triazol-1-yl}}}phenyl}}}}-1,3,4-thiadiazol-2-amine (7)**

Compound **6** (10 mmol, 4.42 g) was dissolved in ethanol (10 mL), followed by the addition of hydrazine carbothioamide (10 mmol, 0.91 g) and concentrated H_2_SO_4_ 98% (10 mmol, 0.54 mL). The reaction mixture was refluxed at 100–150 °C for 16 h, and its progress was monitored by TLC using benzene:acetone (5:1) as the eluent until completion. After cooling to room temperature, the obtained solid appeared and was collected by vacuum filtration, thoroughly washed with cold DCM, and recrystallized from methanol to afford compound **7** as a dark yellow powder. Yield: 70%; m.p. 255–257 °C. IR (KBr) cm^−1^, *ν*: 3487, 3447 (NH_2_), 3072 (Ar–CH), 2927 (aliphatic CH), 1634, 1627 (C=N), 1510, 1443 (Ar C=C), 1403 (N=N). ^1^H NMR (DMSO-*d*_6_, 400 MHz) δ: 2.39 (s, 3H, CH_3_), 3.88 (s, 3H, OCH_3_), 5.22 (s, 2H, CH_2_), 6.02 (brs, 2H, NH_2_, exchangeable), 6.61–7.89 (m, 11H, Ar–H), 8.10 (s, 1H, CH triazole), 8.55 (s, 1H, CH=N) ppm. ^13^C NMR (DMSO-*d*_6_, 100 MHz) δ: 20.34 (CH_3_), 56.69 (OCH_3_), 72.55 (CH_2_), 113.08 (CH triazole), 113.70–130.09 (17Ar-C), 139.15 (C triazole), 151.00 (Ar-C–OCH_3_), 159.99 (C=N), 160.00, 171.92 (C=N thiadiazol) ppm. Analysis calcd. for C_26_H_23_N_7_O_2_S (497.16): C, 62.76; H, 4.66; N, 19.71. Found: C, 62.75; H, 4.66; N, 19.70%.

**(*E*)-1-{{{{{5-{{{{4-{{{4-{{{2-Methoxy-4-[(p-tolylimino)methyl]phenoxy}methyl}}-1*H*-1,2,3-triazol-1-yl}}phenyl}}}}-1,3,4-thiadiazol-2-yl}}}}}pyrrolidine-2,5-dione (8)**

The mixture of compound **7** (10 mmol, 4.97 g) was dissolved in EtOH (10 mL), followed by the addition of succinic anhydride (10 mmol, 1.0 g) and triethylamine (10 mmol, 1.01 mL). The reaction mixture was refluxed at 100–150 °C for 12 h. The progress of the reaction was monitored by TLC using benzene/acetone (5:1) as the eluent. After completion, the reaction mixture was cooled to room temperature and poured into chilled water (50 mL). The resulting solid was collected by vacuum filtration, thoroughly washed with cold ethanol, and recrystallized from ethanol to afford compound **8** as a dark brown powder. Yield: 60%; m.p. > 300 °C. IR (KBr) cm^−1^, *ν*: 3062 (Ar–CH), 2956, 2922, 2856 (aliphatic CH), 1715 (2C=O), 1636, 1591 (3C=N), 1512, 1429 (Ar C=C), 1392 (N=N). ^1^H NMR (400 MHz, DMSO-*d*_6_, δ, ppm): 2.38 (s, 3H, CH_3_), 3.14 (t, 4H, *J* = 8.04 Hz, 2CH_2_), 3.88 (s, 3H, OCH_3_), 5.26 (s, 2H, CH_2_), 6.61–7.89 (m, 11H, Ar–H), 8.10 (s, 1H, CH triazole), 8.52 (s, 1H, CH=N). ^13^C NMR (100 MHz, DMSO-*d*_6_, δ, ppm): 20.35 (CH_3_), 30.00 (2CH_2_), 56.44 (OCH_3_), 72.15 (CH_2_), 113.95 (CH triazole), 119.46–131.00 (16Ar–C), 139.69 (C triazole), 151.35 (Ar–C–OCH_3_), 159.16 (C=N), 173.06, 174.69 (2C=N Thiadiazol), 175.22, 176.08 (2C=O). EI-MS: *m*/*z* = 579.81 (M^+^), with a base peak at *m*/*z* 294.44. Anal. calcd. for C_30_H_25_N_7_O_4_S (579.17): C, 62.16; H, 4.35; N, 16.92. Found: C, 62.16; H, 4.35; N, 16.90%.

**(*E*)-3-(*p*-Tolylimino)butanehydrazide (9)**

Hydrazinolysis of compound **2** was achieved by suspending it (10 mmol, 2.19 g) in ethanol (10 mL), followed by the slow addition of hydrazine hydrate (10 mmol, 1.00 mL). The reaction system was heated under reflux at 100–150 °C for 3 h until TLC analysis, using benzene:acetone (5:1) as the solvent system, confirmed complete transformation of the starting material. After the reaction was allowed to cool naturally to room temperature, the mixture was poured into chilled water (50 mL), inducing precipitation of the target product. The precipitate was separated by vacuum filtration, washed repeatedly with cold ethanol, and recrystallized from DMF to furnish compound **9** as a bright yellow powder. Yield: 65%; m.p. > 300 °C. IR (KBr) cm^−1^, *ν*: 3417, 3388, 3228 (NH_2_, NH), 3033 (Ar–CH), 2976, 2924 (aliphatic CH), 1703 (C=O), 1636 (C=N), 1605 (Ar C=C). ^1^H NMR (DMSO-*d*_6_, 400 MHz) δ: 1.76 (t, 3H, *J* = 6.7 Hz, CH_3_–C=N), 2.32 (s, 3H, CH_3_–Ph), 2.74 (s, 2H, CH_2_), 4.24 (s, 2H, NH_2_, exchangeable), 7.26–7.47 (m, 4H, Ar–H), 9.28 (brs, 1H, NH, exchangeable) ppm. ^13^C NMR (DMSO-*d*_6_, 100 MHz) δ: 21.07 (CH_3_–Ph), 26.27 (CH_3_–C=N), 34.27 (CH_2_), 115.84–139.72 (5Ar-C), 145.01 (Ar-C–N), 163.03 (C=N), 176.11 (C=O) ppm. Analysis calcd. for C_11_H_15_N_3_O (205.12): C, 64.37; H, 7.37; N, 20.47. Found: C, 64.39; H, 7.40; N, 20.47%.

**Ethyl (*Z*)-4-(ethylideneamino)-6-methyl-2-(thiophen-2-yl)quinoline-3-carboxylate (10)**

Condensation of compound **3** was achieved by dissolving it (10 mmol, 3.12 g) in methanol (10 mL), followed by the addition of acetaldehyde (10 mmol, 0.56 mL) in the presence of a catalytic amount of acetic acid (AcOH). The reaction mixture was heated under reflux at 100–150 °C for 7 h, and the reaction progress was monitored by TLC using benzene:acetone (5:1) as the developing system until completion. After cooling to room temperature, the reaction mixture was poured into chilled water (50 mL), resulting in the formation of a precipitate. The solid was isolated by vacuum filtration, thoroughly washed with chloroform, and recrystallized from ethanol to afford compound **10** as a dark brownish red powder. Yield: 72%; m.p. 287–289 °C. IR (KBr) cm^−1^, *ν*: 3085, 3028 (Ar–CH), 2973, 2927, 2863 (aliphatic CH), 1713 (C=O), 1594, 1566 (C=N), 1499 (Ar C=C). ^1^H NMR (DMSO-*d*_6_, 400 MHz) δ: 0.92 (d, 3H, *J* = 6.7 Hz, CH_3_–C=N), 1.02 (t, *J* = 6.9 Hz, 3H, CH_3_–CH_2_), 2.22 (s, 3H, CH_3_–Ph), 4.30 (q, 2H, *J*= 9.8 Hz, CH_2_), 7.07–7.74 (m, 6H, Ar–H), 8.70 (q, 1H, *J* = 9.2 Hz, CH=N) ppm. ^13^C NMR (DMSO-*d*_6_, 100 MHz) δ: 10.01 (CH_3_–CHN), 14.04 (CH_3_–CH_2_), 23.00 (CH_3_–Ph), 60.02 (CH_2_), 111.17 (C pyridine–CO), 127.60–131.37 (9Ar-C), 143.06 (C pyridine–N), 155.62 (C=N pyridine), 158.26 (C pyridine–N=C), 162.22 (CH=N), 166.66 (C=O) ppm. Analysis calcd. for C_19_H_18_N_2_O_2_S (338.11): C, 67.43; H, 5.36; N, 8.28. Found: C, 67.44; H, 5.36; N, 8.25%.

**Ethyl 6-methyl-4-(2-methyl-4-oxothiazolidin-3-yl)-2-(thiophen-2-yl)quinoline-3-carboxylate (11)**

Cyclization of compound **10** was performed by dissolving it (10 mmol, 3.38 g) in ethanol (10 mL), followed by the addition of thioglycolic acid (10 mmol, 0.69 mL) and anhydrous Na_2_SO_4_ (10 mmol, 1.42 g). The reaction mixture was heated under reflux at 100–150 °C for 18 h, while the progress was monitored by TLC using benzene:acetone (5:1) as the mobile phase until completion. After cooling to room temperature, the reaction mixture was poured into chilled water (50 mL), leading to the formation of a precipitate. The resulting solid was isolated by vacuum filtration, thoroughly washed with cold ethanol, and recrystallized from ethanol to afford compound **11** as a dark orange powder. Yield: 78%; m.p. 277–279 °C. IR (KBr) cm^−1^, *ν*: 3072, 3033 (Ar–CH), 2972, 2927, 2900, 2880 (aliphatic CH), 1738, 1664 (2C=O), 1608 (C=N), 1530 (Ar C=C). ^1^H NMR (DMSO-*d*_6_, 400 MHz) δ: 1.11 (t, *J* = 7.5 Hz, 3H, CH_3_ ester), 1.85 (d, *J* = 6.7 Hz, 3H, CH_3_), 2.37 (s, 3H, CH_3_–Ph), 4.00 (s, 2H, CH_2_ thiazolidinone), 4.53 (q, 2H, *J* = 8.0 Hz, CH_2_ ester), 4.89 (q, 1H, *J* = 5.4 Hz, CH thiazolidinone), 6.94–7.47 (m, 6H, Ar–H) ppm. ^13^C NMR (DMSO-*d*_6_, 100 MHz) δ: 15.03 (CH_3_ ester), 23.00 (CH_3_–Ph), 25.22 (CH_3_ thiazolidinone), 35.03 (CH_2_ thiazolidinone), 56.00 (CH thiazolidinone), 62.22 (CH_2_ ester), 104.20 (C pyridine–CO), 118.25–139.75 (9Ar-C), 143.33 (C pyridine–N), 152.61 (C pyridine–N thiazolidinone), 155.65 (C=N pyridine), 165.35 (C=O ester), 170.03 (C=O thiazolidinone) ppm. Analysis calcd. for C_21_H_20_N_2_O_3_S_2_ (412.09): C, 61.14; H, 4.89; N, 6.79. Found: C, 61.15; H, 4.94; N, 6.79%.

**Ethyl 4-(5-acetyl-2-methyl-4-oxothiazolidin-3-yl)-6-methyl-2-(thiophen-2-yl)quinoline-3-carboxylate (12)**

Acetylative modification of compound **11** was carried out by dissolving it (10 mmol, 4.12 g) in THF (10 mL), followed by the successive addition of acetyl chloride (10 mmol, 0.55 mL) and NaH (10 mmol, 0.24 g). The reaction mixture was stirred at room temperature for 24 h, while the progress was monitored by TLC using benzene:acetone (5:1) as the mobile phase until completion. Upon completion, precipitate appeared. The solid was isolated by vacuum filtration, washed thoroughly with cold ethanol, and recrystallized from diethylether to furnish compound **12** as an off-white brown powder. Yield: 90%; m.p. 212–214 °C. IR (KBr) cm^−1^, *ν*: 3069, 3031 (Ar–CH), 2986, 2926, 2855 (aliphatic CH), 1720, 1708, 1656 (3C=O), 1608 (C=N), 1529 (Ar C=C). ^1^H NMR (DMSO-*d*_6_, 400 MHz) δ: 1.11 (t, 3H, *J* = 5.5 Hz, CH_3_ ester), 1.82 (d, 3H, *J* = 7.4 Hz, CH_3_), 2.31 (s, 3H, CH_3_–Ph), 2.48 (s, 3H, CH_3_–CO), 4.24 (s, 1H, CH thiazolidinone–CO), 4.52 (q, 2H, *J* = 6.5 Hz, CH_2_ ester), 4.92 (q, 1H, *J*=7.7 Hz, CH thiazolidinone), 7.07–8.58 (m, 6H, Ar–H) ppm. ^13^C NMR (DMSO-*d*_6_, 100 MHz) δ: 15.14 (CH_3_ ester), 23.23 (CH_3_–Ph), 25.77 (CH_3_ thiazolidinone), 29.97 (CH_3_ acetyl), 55.14 (CH thiazolidinone), 62.55 (CH_2_ ester), 67.57 (CH thiazolidinone–CO), 101.52 (C pyridine–CO), 113.41–139.35 (Ar-C), 141.09 (C pyridine–N), 151.24 (C pyridine–N thiazolidinone), 155.97 (C=N pyridine), 165.31 (C=O ester), 166.16 (C=O thiazolidinone), 196.82 (C=O acetyl) ppm. Analysis calcd. for C_23_H_22_N_2_O_4_S_2_ (454.10): C, 60.77; H, 4.88; N, 6.16. Found: C, 60.72; H, 4.88; N, 6.18%.

**Ethyl 4-[5-(3-chloropropyl)-2-methyl-4-oxothiazolidin-3-yl]-6-methyl-2-(thiophen-2-yl)quinoline-3-carboxylate (13)**

Alkylation of compound **11** was carried out by suspending it (10 mmol, 4.54 g) in sodium ethoxide solution (10 mL), followed by the addition of dichloropropane (10 mmol, 0.94 mL). The reaction mixture was stirred for 9 h, and the progress of the reaction was monitored by TLC using benzene:acetone (5:1) as the eluent until completion. After completion, precipitate was obtained and isolated by vacuum filtration, thoroughly washed with methanol, and recrystallized from ethanol to afford compound **13** as a dark brown powder. Yield: 88%; m.p. 267–269 °C. IR (KBr) cm^−1^, *ν*: 3079, 3034 (Ar–CH), 2979, 2928, 2886, 2816 (aliphatic CH), 1738, 1663 (2C=O), 1596 (C=N), 1514 (Ar C=C), 765 (C–Cl). ^1^H NMR (DMSO-*d*_6_, 400 MHz) δ: 1.11 (t, 3H, *J* = 7.6 Hz, CH_3_ ester), 1.30 (dd, 2H, *J* = 6.8 Hz, CH_2_), 1.81 (d, 3H, *J* = 6.6 Hz, CH_3_), 2.01 (q, 2H, *J* = 8.2 Hz, CH_2_), 2.38 (s, 3H, CH_3_–Ph), 3.59 (t, 1H, *J* = 8.6 Hz, CH thiazolidinone), 3.89 (t, 2H, *J* = 5.2 Hz, CH_2_–Cl), 4.53 (q, 2H, *J* = 6.1 Hz, CH_2_ ester), 4.82 (q, 1H, *J* = 7.6 Hz, CH thiazolidinone), 7.16–7.52 (m, 6H, Ar–H) ppm. ^13^C NMR (DMSO-*d*_6_, 100 MHz) δ: 15.00 (CH_3_ ester), 23.00 (CH_3_–Ph), 26.06 (CH_3_ thiazolidinone), 29.94, 31.00 (2CH_2_), 43.33 (CH_2_–Cl), 47.14, 56.13 (2CH thiazolidinone), 62.13 (CH_2_ ester), 101.82 (C pyridine–CO), 113.41–137.01 (9Ar-C), 145.44 (C pyridine–N), 153.94 (C pyridine–N thiazolidinone), 155.17 (C=N pyridine), 165.22 (C=O ester), 173.79 (C=O thiazolidinone) ppm. EI-MS: *m*/*z* = 492.45 (M^+^, isotopic peak), 361.05 (base peak). Analysis calcd. for C_24_H_25_ClN_2_O_3_S_2_ (488.10): C, 58.94; H, 5.15; N, 5.73. Found: C, 58.92; H, 5.15; N, 5.73%.

**Ethyl 6-methyl-4-[2-methyl-5-(3-morpholinopropyl)-4-oxothiazolidin-3-yl]-2-(thiophen-2-yl)quinoline-3-carboxylate (14)**

Nucleophilic substitution of compound **13** was carried out by dissolving it (10 mmol, 4.88 g) in acetonitrile (10 mL), followed by the addition of morpholine (15 mmol, 0.9 mL) and triethylamine (20 mmol, 1.5 mL). The reaction mixture was heated under reflux at 100–150 °C for 16 h, and the progress of the reaction was monitored by TLC using benzene:acetone (5:1) as the developing solvent system until completion. After cooling to room temperature, the reaction mixture was poured into chilled water (50 mL), resulting in the precipitation of the product. The formed solid was isolated by vacuum filtration, thoroughly washed with cold water, and recrystallized from chloroform to afford compound **14** as a dark brown powder. Yield: 79%; m.p. 281–283 °C. IR (KBr) cm^−1^, *ν*: 3080, 3036 (Ar–CH), 2999, 2949, 2920, 2897, 2849 (aliphatic CH), 1738, 1683 (2C=O), 1596 (C=N), 1526 (Ar C=C). ^1^H NMR (DMSO-*d*_6_, 400 MHz) δ: 1.11 (t, 3H, *J* = 7.6 Hz, CH_3_ ester), 1.39 (dd, 2H, *J* = 7.5 Hz, CH_2_), 1.82 (d, 3H, *J* = 5.6 Hz, CH_3_), 2.09 (q, 2H, *J* = 4.6 Hz, CH_2_), 2.39 (s, 3H, CH_3_–Ph), 2.70 (t, 4H, *J* = 7.8 Hz, NCH_2_ morpholine), 2.84 (t, 2H, *J* = 7.6 Hz, CH_2_ morpholine), 3.29 (t, 1H, CH thiazolidinone), 3.73 (t, 4H, *J* = 7.6 Hz, OCH_2_ morpholine), 4.50 (q, 2H, *J* = 5.6 Hz, CH_2_ ester), 4.84 (q, 1H, *J* = 7.9 Hz, CH thiazolidinone), 6.50–8.47 (m, 6H, Ar–H) ppm. ^13^C NMR (DMSO-*d*_6_, 100 MHz) δ: 15.12 (CH_3_ ester), 23.13 (CH_3_–Ph), 25.91 (CH_3_ thiazolidinone), 26.94, 31.13 (2CH_2_), 48.12, 55.44 (2CH thiazolidinone), 58.10 (CH_2_ morpholine), 63.10 (CH_2_ ester), 67.13, 69.95 (OCH_2_ morpholine), 100.05 (C pyridine–CO), 113.95–140.01 (9Ar-C), 145.17 (C pyridine–N), 153.35 (C pyridine–N thiazolidinone), 156.35 (C=N pyridine), 165.22 (C=O ester), 173.06 (C=O thiazolidinone) ppm. EI-MS: *m*/*z* = 539.02 (M^+^), 508.23 (base peak). Analysis calcd. for C_28_H_33_N_3_O_4_S_2_ (539.19): C, C, 62.31; H, 6.16; N, 7.79. Found: C, 62.32; H, 6.16; N, 7.81%.

### 3.2. Biological Activity

All in vitro studies were described in depth in the Supplementary Materials.

#### 3.2.1. Assessment of Antiproliferative Activity

The cytotoxic activity of the synthesized compounds (**1**–**14**) was assessed using the MTT colorimetric assay against three human cancer cell lines: HCT-116 (colorectal carcinoma) [135], HepG-2 (hepatocellular carcinoma) [136], and MCF-7 (breast adenocarcinoma) [137,138], in addition to the non-cancerous WI-38 human lung fibroblast cell line [139] to evaluate their safety profile. The assay was performed following previously reported procedures, and the tested compounds exhibited variable micromolar IC_50_ values that were comparable to those of the reference drug doxorubicin [140,141]. The full experimental protocol for the MTT assay is described in the Supplementary Materials.

#### 3.2.2. Evaluation of VEGFR-2 Enzyme Inhibitory Activity In Vitro

The inhibitory activity of compound **14** against VEGFR-2 was evaluated using a VEGFR-2 (KDR) kinase assay kit (BPS Bioscience, San Diego, CA, USA) according to the manufacturer’s instructions. The assay was performed in a 96-well plate format, and kinase activity was quantified using a luminescent detection method [142,143]. Sorafenib was used as a reference inhibitor, and IC_50_ values were calculated from dose response curves generated using Excel software. Detailed experimental procedures are provided in the Supplementary Materials.

#### 3.2.3. Wound-Healing Assay

Cell migration was assessed using a wound-healing (scratch) assay. Briefly, a confluent cell monolayer was mechanically scratched using a sterile 200 μL pipette tip, followed by washing with PBS and incubation with fresh medium containing the tested compound. Images were captured at 0 h and after incubation, and wound closure was quantified to evaluate cell migration [144,145,146]. A detailed experimental protocol is provided in the Supplementary Materials.

#### 3.2.4. Cell Cycle Arrest and Apoptosis

Cell cycle distribution and apoptosis were analyzed by flow cytometry in HepG-2 cells following treatment with compound **14**. Cell cycle phases (G_0_/G_1_, S, and G_2_/M) were determined after propidium iodide (PI) staining [147], while apoptosis was assessed using Annexin V-FITC/PI dual staining, according to the manufacturer’s protocols [148]. Data acquisition and analysis were performed using a Gallios flow cytometer (Beckman Coulter, Brea, CA, USA) and Kaluza software (version 2.1), and results were expressed as percentages of cells in each population. A detailed experimental protocol is provided in the Supplementary Materials.

#### 3.2.5. Caspase-3 Mediated Apoptotic Pathway Activation

Active caspase-3 levels in HepG-2 cells were quantified using a commercial human caspase-3 (active) ELISA kit (Invitrogen, Thermo Fisher Scientific, Waltham, MA, USA) according to the manufacturer’s instructions. Absorbance was measured at 450 nm, and caspase-3 activity was expressed as fold change relative to untreated control cells. The involvement of caspase-3 as a key executioner protease in apoptosis and the reliability of colorimetric/fluorometric caspase detection assays have been well documented [149,150]. Detailed experimental procedures, including cell lysis, ELISA conditions, and data analysis, are provided in the Supplementary Materials.

### 3.3. In Silico Analyses

#### 3.3.1. ADMET Prediction

The physicochemical and ADMET-related properties of compound **14** were predicted using the ADMETlab 3.0 online platform (https://admetlab3.scbdd.com/, accessed on 31 January 2026). The analysis included molecular weight (MW), lipophilicity (logP and logD), aqueous solubility (logS), topological polar surface area (TPSA), numbers of hydrogen bond acceptors and donors (nHA and nHD), rotatable bonds (nRot), ring count and maximum ring size, heteroatom content, fraction of charged atoms, and molecular flexibility. These parameters were visualized using a radar plot, in which the shaded region represents the optimal drug-like physicochemical space. In addition, gastrointestinal absorption and blood–brain barrier permeation were predicted using the GI/BBB absorption (BOILED-EGG) model implemented in ADMETlab 3.0, which estimates passive intestinal absorption and brain penetration based on the relationship between lipophilicity and polar surface area [151].

#### 3.3.2. Molecular Docking Study

Molecular docking was performed to investigate the binding behavior of compound **14** toward VEGFR-2, HIV-1 protease, hepatitis virus polymerase, and SARS-CoV-2 main protease (Mpro, Mumbai, Maharashtra). Protein structures were retrieved from the Protein Data Bank (RCSB Protein Data Bank, Rutgers University, Piscataway, NJ, USA) and prepared using PyMOL (version 2.5, Schrödinger LLC, New York, NY, USA) [152] and AutoDock Tools (version 1.5.7, The Scripps Research Institute, La Jolla, CA, USA) [153], while docking simulations were carried out using AutoDock Vina (version 1.2.3, The Scripps Research Institute, La Jolla, CA, USA) [154]. Protein–ligand interactions were visualized and analyzed using BIOVIA Discovery Studio Visualizer (version 4.5, Dassault Systèmes, San Diego, CA, USA) [155]. Detailed experimental procedures are provided in the Supplementary Materials.

#### 3.3.3. Molecular Dynamics Simulation

Molecular dynamics simulations were performed using GROMACS 2018 [156,157] to evaluate the dynamic stability of the VEGFR-2–compound **14** complex. The system was modeled using the CHARMM36 force field [158] and analyzed over a 100 ns simulation using RMSD, RMSF, radius of gyration, solvent-accessible surface area, and hydrogen-bond metrics to assess structural stability and interaction persistence.

#### 3.3.4. Quantum Chemical Calculations

Density Functional Theory (DFT) calculations were performed using Gaussian 09 [159] at the B3LYP/6-311G++(d,p) [160,161] level to optimize all molecular geometries. All optimized structures were verified as true minima by the absence of imaginary vibrational frequencies. Frontier molecular orbital (FMO) analysis was employed to calculate key global reactivity descriptors, including the HOMO–LUMO energy gap (ΔE), electronegativity (χ), chemical hardness (η), softness (σ), electrophilicity index (ω), and ionization potential. Density of states (DOS) analyses were conducted to evaluate the distribution of electronic states and to further assess frontier orbital characteristics. Molecular electrostatic potential (ESP) maps, projected onto the electron density surface, were generated to identify electrophilic and nucleophilic regions and to rationalize charge distribution patterns. In addition, noncovalent interaction (NCI) analyses, based on the reduced density gradient (RDG) approach, were performed to visualize and characterize weak intermolecular interactions, including attractive and repulsive regions.

## 4. Conclusions

In the present study, a series of *p*-toluidine-based derivatives was rationally designed, synthesized, and structurally characterized, followed by a comprehensive evaluation of their anticancer potential. Antiproliferative screening identified compound **14** as one of the most active members of the series, exhibiting notable activity against hepatocellular carcinoma cells with a favorable selectivity profile toward normal cells. Mechanistic investigations indicated that its anticancer effects may involve multiple complementary pathways, including VEGFR-2 inhibition, suppression of cancer cell migration, induction of G_0_/G_1_ cell-cycle arrest, and activation of caspase-3-dependent apoptosis, supporting a regulated mechanism of action rather than nonspecific cytotoxicity. Density functional theory (DFT) calculations provided supportive molecular-level insights into the behavior of compound **14**, revealing favorable electronic features, including an optimized HOMO–LUMO distribution and well-defined electron-rich regions that may contribute to its interaction with biological targets. In agreement with these observations, molecular docking and molecular dynamics simulations suggested a stable binding mode of compound **14** within the VEGFR-2 active site. Furthermore, ADMET and BOILED-EGG analyses indicated acceptable drug-like characteristics, although certain predicted pharmacokinetic limitations suggest that additional structural optimization may be required. Overall, the integrated biological and computational findings suggest that compound **14** represents a structurally promising scaffold for the development of VEGFR-2-targeted anticancer agents and highlight the potential of rational modification of the *p*-toluidine framework for future lead optimization efforts.

## Figures and Tables

**Table 1:** Reported VEGFR-2 inhibitory activities of selected reference compounds and sorafenib from the literature.

Structural Class	Compound	IC_50_ (µM)	Reference
Quinoline-based	**V**	0.0985	[109]
	Foretinib (**VI**)	0.0009	[110,111]
	**VII**	0.036	[112]
	**VIII**	0.0038	[113]
Thiazolidin-4-one	**IX**	0.079	[114]
	**X**	0.0841	[115]
	**XI**	0.081	[116]
Morpholine-containing	**XII**	0.049	[117]
	**XIII**	1.52	[118]
Reference drugs	Sorafenib	0.0370–1.4600	[109,112,113,114,115,116,117]
	Sunitinib	1.2	[118]

All IC_50_ values are presented in µM units for consistency and were converted from the original reports where necessary. The data are shown as reported in the cited studies.

**Table 2:** In vitro antiproliferative activity (IC_50_, µM) of compounds **1**–**14** against HCT-116, HepG-2, MCF-7, and WI-38 cell lines. Data are presented as mean ± SD (*n* = 3). DOX and SOR were used as reference drugs.

Comp.			In Vitro Cytotoxicity IC_50_ (µM)				
	WI-38	HCT-116	SI (HCT-116)	HePG-2	SI (HePG-2)	MCF-7	SI (MCF-7)
**DOX**	6.72 ± 0.5	5.23 ± 0.3	1.28	4.50 ± 0.2	1.49	4.17 ± 0.2	1.61
**SOR**	10.65 ± 0.8	5.47 ± 0.3	1.95	9.18 ± 0.6	1.16	7.26 ± 0.3	1.47
**1**	34.76 ± 2.3	60.45 ± 3.4	0.57	56.16 ± 3.3	0.62	52.68 ± 3.1	0.66
**2**	49.53 ± 3.0	9.61 ± 0.8	5.15	11.63 ± 0.9	4.26	6.31 ± 0.4	7.85
**3**	>100	94.20 ± 5.1	-	87.05 ± 4.3	-	81.63 ± 4.0	-
**4**	55.48 ± 3.2	13.42 ± 1.1	4.13	19.53 ± 1.4	2.84	8.72 ± 0.7	6.36
**5**	64.26 ± 3.6	45.70 ± 2.6	1.41	42.85 ± 2.6	1.50	33.45 ± 2.2	1.92
**6**	47.52 ± 2.8	39.82 ± 2.4	1.19	36.15 ± 2.3	1.31	29.71 ± 1.9	1.60
**7**	73.38 ± 3.8	>100	-	92.27 ± 4.8	0.80	>100	-
**8**	32.74 ± 2.1	83.54 ± 4.2	0.39	78.16 ± 3.9	0.42	75.42 ± 3.9	0.43
**9**	18.17 ± 1.3	53.24 ± 3.1	0.34	59.44 ± 3.4	0.31	48.97 ± 2.8	0.37
**10**	>100	51.63 ± 2.9	-	46.59 ± 2.8	-	40.84 ± 2.5	-
**11**	27.63 ± 1.8	76.19 ± 3.9	0.36	68.75 ± 3.7	0.40	57.43 ± 3.4	0.48
**12**	85.26 ± 4.4	72.54 ± 3.8	1.18	61.78 ± 3.5	1.38	63.57 ± 3.5	1.34
**13**	58.84 ± 3.4	31.04 ± 1.9	1.89	20.02 ± 1.5	2.94	26.13 ± 1.7	2.25
**14**	67.75 ± 3.6	22.56 ± 1.4	3.00	7.84 ± 0.5	8.64	17.80 ± 1.4	3.81

(-) is not detected; SD stands for standard deviation; each number is the mean of three values; IC_50_ is the compound concentration needed to inhibit growth by 50%.

**Table 3:** Group efficiency analysis for the structural evolution of compounds **1**–**14** (HepG-2 cells).

Transformation	IC_50_ Start (µM)	IC_50_ Final (µM)	pIC_50_ Start	pIC_50_ Final	ΔpIC_50_	Heavy Atoms Added	GE
1→4	56.16	19.53	4.25	4.71	0.46	10	0.05
4→5	19.53	42.85	4.71	4.37	−0.34	9	−0.04
5→6	42.85	36.15	4.37	4.44	0.07	2	0.04
6→7	36.15	92.27	4.44	4.03	−0.41	5	−0.08
7→8	92.27	78.16	4.03	4.11	0.08	6	0.01
2→9	11.63	59.44	4.93	4.23	−0.70	2	−0.35
3→10	87.05	46.59	4.06	4.33	0.27	3	0.09
10→11	46.59	68.75	4.33	4.16	−0.17	6	−0.03
11→12	68.75	61.78	4.16	4.21	0.05	3	0.02
11→13	68.75	20.02	4.16	4.7	0.54	4	0.14
13→14	20.02	7.84	4.7	5.11	0.41	6	0.07

Group efficiency equation: GE = ΔpIC_50_/number of heavy atoms added.

**Table 4:** Inhibitory activity of compound **14** against VEGFR-2 expressed as IC_50_ values (µM) in comparison with the reference drug sorafenib.

Compd. No.	IC_50_ (Mean ± SD) (µM)
**14**	0.55 ± 0.02
**Sorafenib**	0.17 ± 0.01

Data are presented as mean ± SD of three independent experiments. Statistical significance between compound **14** and sorafenib was evaluated using an unpaired Student’s *t*-test (*p* < 0.05).

**Table 5:** Quantitative analysis of wound closure (%) in HepG-2 cells after 72 h of treatment with compound **14** compared to untreated control cells. Data are expressed as mean ± SD.

Compound No.	% Closure 72 h
**14/HePG-2**	63.704 ± 2.35
**Cont./HePG-2**	97.037 ± 3.58

**Table 6:** Quantitative determination of active caspase-3 levels (pg/mL) in HepG-2 cells treated with compound **14** compared to untreated control cells. Data are expressed as mean ± SD.

Compound No.	Caspase 3-Pg/mL	Fold
**14/HePG-2**	530.21 ± 20.6	5.319
**Cont./HePG2**	99.69 ± 3.87	1.000

**Table 7:** DFT-calculated frontier molecular orbital energies and global reactivity descriptors, including HOMO, LUMO, HOMO–LUMO energy gap (ΔE), chemical hardness (η), softness (σ), electronegativity (χ), electrophilicity index (ω), and maximum charge transfer (ΔN) for compounds **1**–**14**.

File Name	LUMO	HOMO	ΔE	A	I	X	η	S or σ	ω	ΔN_max_	ΔN
**1**	−0.16384	−0.27031	0.10647	0.16384	0.27031	0.217075	0.053235	18.78463417	0.885161184	2.038837231	63.70738236
**2**	−0.15594	−0.30892	0.15298	0.15594	0.30892	0.23243	0.07649	13.07360439	0.706284546	1.519348935	44.23826644
**3**	−0.18792	−0.26223	0.07431	0.18792	0.26223	0.225075	0.037155	26.91427802	1.363443833	3.028865563	91.17110752
**4**	−0.17998	−0.28017	0.10019	0.17998	0.28017	0.230075	0.050095	19.96207206	1.056682416	2.296386865	67.57086536
**5**	−0.17693	−0.28746	0.11053	0.17693	0.28746	0.232195	0.055265	18.09463494	0.975563522	2.10074188	61.23048041
**6**	−0.17698	−0.2861	0.10912	0.17698	0.2861	0.23154	0.05456	18.32844575	0.982602119	2.121884164	62.02767595
**7**	−0.18356	−0.2271	0.04354	0.18356	0.2271	0.20533	0.02177	45.93477262	1.936628797	4.715893431	156.0558107
**8**	−0.17906	−0.22860	0.04954	0.17906	0.2286	0.20383	0.02477	40.37141704	1.677297897	4.114452967	137.1855067
**9**	−0.15731	−0.27163	0.11432	0.15731	0.27163	0.21447	0.05716	17.49475157	0.804712752	1.876049685	59.35558083
**10**	−0.16424	−0.1998	0.03556	0.16424	0.1998	0.18202	0.01778	56.24296963	1.863401597	5.118672666	191.731721
**11**	−0.18684	−0.23087	0.04403	0.18684	0.23087	0.208855	0.022015	45.42357484	1.981395005	4.743470361	154.2390416
**12**	−0.2262	−0.26118	0.03498	0.2262	0.26118	0.24369	0.01749	57.17552887	3.395358268	6.966552316	193.1477987
**13**	−0.19017	−0.21831	0.02814	0.19017	0.21831	0.20424	0.01407	71.0732054	2.964746098	7.257995736	241.4982232
**14**	−0.17391	−0.18414	0.01023	0.17391	0.18414	0.179025	0.005115	195.5034213	6.265875	17.5	666.7619746

## Data Availability

The original contributions presented in this study are included in the article/Supplementary Materials. Further inquiries can be directed to the corresponding authors.

## Supplementary Materials

The following supporting information can be downloaded at https://www.mdpi.com/article/10.3390/ijms27073018/s1.

## Abbreviations

The following abbreviations are used in this manuscript:

ADMET	Absorption, Distribution, Metabolism, Excretion, and Toxicity
Akt	Protein Kinase B
ATP	Adenosine Triphosphate
BBB	Blood–Brain Barrier
CADD	Computer-Aided Drug Design
CNS	Central Nervous System
CYP	Cytochrome P450
DFG	Asp–Phe–Gly Motif
DFT	Density Functional Theory
DOS	Density of States
DUD-E	Database of Useful Decoys: Enhanced
ELISA	Enzyme-Linked Immunosorbent Assay
ELF	Electron Localization Function
ERK	Extracellular Signal-Regulated Kinase
ESP	Electrostatic Potential
FDA	Food and Drug Administration
FITC	Fluorescein Isothiocyanate
FMO	Frontier Molecular Orbitals
FGFR	Fibroblast Growth Factor Receptor
GE	Group Efficiency
GI	Gastrointestinal
HBD	Hydrogen Bond Donor
HCC	Hepatocellular Carcinoma
HIV	Human Immunodeficiency Virus
HOMO	Highest Occupied Molecular Orbital
IC_50_	Half Maximal Inhibitory Concentration
IR	Infrared Spectroscopy
k_off	Dissociation Rate Constant
LogP	Partition Coefficient
logD	Distribution Coefficient
logS	Logarithm of Aqueous Solubility
LUMO	Lowest Unoccupied Molecular Orbital
MD	Molecular Dynamics
Mpro	Main Protease
MS	Mass Spectrometry
MTT	3-(4,5-Dimethylthiazol-2-yl)-2,5-diphenyltetrazolium bromide
MW	Molecular Weight
NCI	Non-Covalent Interaction
nHA	Number of Hydrogen Bond Acceptors
nHD	Number of Hydrogen Bond Donors
nRot	Number of Rotatable Bonds
PBS	Phosphate-Buffered Saline
PDB	Protein Data Bank
PI	Propidium Iodide
RDG	Reduced Density Gradient
RMSD	Root Mean Square Deviation
RMSF	Root Mean Square Fluctuation
Rg	Radius of Gyration
R^2^	Coefficient of Determination
SAR	Structure-Activity Relationship
SARS-CoV-2	Severe Acute Respiratory Syndrome Coronavirus 2
SASA	Solvent-Accessible Surface Area
SD	Standard Deviation
SI	Selectivity Index
TPSA	Topological Polar Surface Area
VEGF	Vascular Endothelial Growth Factor
VEGFR	Vascular Endothelial Growth Factor Receptor
VEGFR-2	Vascular Endothelial Growth Factor Receptor-2
ΔE	HOMO–LUMO Energy Gap
ΔG	Gibbs Free Energy
ΔN_max_	Maximum Charge Transfer
ΔpIC_50_	Change in the negative logarithm of IC_50_
χ	Electronegativity
η	Chemical Hardness
σ	Chemical Softness
ω	Electrophilicity Index
λ_2_	Second Eigenvalue of the Electron Density Hessian Matrix
^1^H NMR	Proton Nuclear Magnetic Resonance
^13^C NMR	Carbon-13 Nuclear Magnetic Resonance
