# Supplementary material for: From Synthesis to Mechanism: Biological Evaluation of a p-Toluidine-Based Thiazolidinone-Quinoline VEGFR-2 Candidate Supported by CADD
Source: Int J Mol Sci. 2026 Mar 26;27(7):3018. doi: 10.3390/ijms27073018 (PMC13073930; doi:10.3390/ijms27073018)
Supplement: Supplementary file 1 [file ijms-27-03018-s001.zip › ijms-4184049-supplementary.pdf]

From Synthesis to Mechanism: Biological Evaluation of a p-Toluidine-Based Thiazolidinone-Quinoline VEGFR-2 Candidate Supported by CADD

Emad Manni [1], Modather F. Hussein [2,*], Sara Elkady [3], Adel A.-H. Abdel-Rahman [3], Mohamed A. Hawata [3], Wael A. El-Sayed [4] and Ahmed F. El-Sayed [5,6] and Hagar S. El-Hema [7,*]

[1] Department of Clinical Laboratory Sciences, College of Applied Medical Sciences, Jouf University, Sakaka 72388, Saudi Arabia
[2] Chemistry Department, College of Science, Jouf University, P.O. Box 2014, Sakaka 72341, Saudi Arabia
[3] Chemistry Department, Faculty of Science, Menoufia University, Shebin El-Kom 32511, Egypt
[4] Photochemistry Department, National Research Centre, Dokki, Giza 12622, Egypt
[5] Microbial Genetics Department, Biotechnology Research Institute, National Research Centre, Giza 12622, Egypt; ahmedfikry.nrc@gmail.com
[6] Egypt Center for Research and Regenerative Medicine (ECRRM), Cairo 11517, Egypt
[7] Basic Science Department (Chemistry), Thebes Higher Institute for Engineering, Thebes Academy, Maadi 11434, Egypt
* Correspondence: mfhussin@ju.edu.sa (M.F.H.); hagarsabry.23@yahoo.com (H.S.E.-H.)

Content

- Exploratory Molecular Docking of Compound **14** against Selected Viral Enzymes
- Experimental of chemistry, biological evaluation, Molecular Docking, Molecular Dynamic Simulations, and quantum chemical calculations.
- **Table S1**. The percentage cytotoxicity of the active compounds on HepG-2, HCT-116, and MCF-7, and normal WI38 cell lines at different concentrations.
- **Table S2**. Detailed results of the VEGFR-2 enzyme inhibitory assay of -based derivative **14**.
- **Table S3**. Detailed results of wound healing assay of **14** within HePG-2
- **Table S4**. Cell cycle analysis after 48 h incubation with compound **14** compared with untreated HepG-2 cells.
- **Table S5**. Apoptosis induction analysis within HepG-2 cells treated with compound **14** compared with untreated HepG-2 cells.
- **Table S6**. Raw absorbance values obtained directly from the ELISA plate reader for caspase-3 assay

- **Table S7**. Calculated caspase-3 concentrations derived from the standard calibration curve for HepG-2 cell samples.
- **Table S8**. Predicted pharmacokinetic, physicochemical, metabolism, excretion, and absorption–distribution related properties of compound **14** in comparison with doxorubicin. The table summarizes comprehensive ADMET descriptors, including molecular weight, lipophilicity, aqueous solubility, topological polar surface area, hydrogen-bond donors and acceptors, metabolic enzyme interactions, clearance, solubility and permeability parameters, and medicinal chemistry indices.
- **Table S9**. *In silico* toxicity risk and drug-likeness prediction of compound **14** in comparison with sorafenib. The table reports predicted mutagenicity, tumorigenicity, irritation, reproductive toxicity, and composite drug-likeness and drug score parameters.
- **Table S10**. Target proteins used for molecular docking, including PDB identifiers, crystal structure resolution, active-site coordinates, co-crystallized ligands, and literature references.
- **Table S11.** Key molecular interactions of compound **14**, sorafenib, and doxorubicin within the VEGFR-2 active site.
- **Table S12.** Experimentally validated VEGFR-2 inhibitors used as positive controls with reported binding affinities and docking scores.
- **Table S13.** Category A negative controls: property-matched DUD-E decoy compounds with docking scores.
- **Table S14.** Category B negative controls: structurally unrelated molecules used to evaluate docking specificity.
- **Table S15.** Integrated statistical analysis comparing docking scores of positive and negative control compounds.
- **Table S16.** Molecular docking interactions of compound **14** with selected viral enzymes.
- **Table S17**. Graphical representations of the frontier molecular orbitals (HOMO and LUMO) of the investigated compounds obtained from DFT calculations, illustrating the spatial distribution of electron density in the highest occupied and lowest unoccupied molecular orbitals.

- **Table S18.** Electron density surfaces of compounds **1–14** generated from total SCF density, showing the spatial distribution of electron density and supporting the HOMO-LUMO and ESP analyses.
- **Figure S1–S46**. Copies of IR, mass, ^{1}H NMR, and ^{13}C NMR spectra of Compounds.
- **Figure S47**. Dose-response IC_{50} curves of compounds **1–14**, Doxorubicin (Dox), and Sorafenib (Sor) against HepG-2, HCT-116, and MCF-7cell lines against normal WI-38 cell lines. % cell viability was plotted versus log concentration (μM), and IC_{50} values were derived using GraphPad Prism (non-linear regression, n = 3, mean ± SD).
- **Figure S48**. ADMET radar plot of compound **14** illustrating its physicochemical profile in relation to the optimal drug-like space. The blue shaded region represents the recommended range for orally active compounds, while the plotted profile of compound **14** reflects the balance between lipophilicity, polarity, hydrogen-bonding capacity, molecular flexibility, and structural complexity.
- **Figure S49.** BOILED-EGG model illustrating the predicted gastrointestinal absorption and brain penetration behavior of compound **14**. The white region represents compounds with a high probability of passive gastrointestinal absorption, while the yellow region indicates the physicochemical space associated with blood–brain barrier penetration. The position of compound **14** (red marker) within the white region and outside the yellow region suggests favorable intestinal absorption with limited central nervous system exposure.
- **Figure S50**. Molecular docking analysis of compound **14** with selected viral enzymes. (**a-c**) 3D binding pose, surface representation, and 2D interaction map of compound **14** within the active site of HIV-1 protease (PDB ID: 3NU3). (**d-f**) 3D binding pose, surface representation, and 2D interaction map of compound **14** within the active site of hepatitis virus polymerase (PDB ID: 7LUF). (**g-i**) 3D binding pose, surface representation, and 2D interaction map of compound **14** within the active site of SARS-CoV-2 main protease (Mpro) (PDB ID: 7ZB7). The figures illustrate the binding orientations and key hydrogen bonding and hydrophobic interactions stabilizing the ligand enzyme complexes.

2.3.2. Molecular Docking

A. Positive Control Set: Known VEGFR-2 Inhibitors

To evaluate the performance of the docking protocol, a set of eight experimentally validated VEGFR-2 inhibitors with reported binding affinities was compiled from crystallographic databases and literature sources. These included sorafenib (PDB: 4ASD, IC_{50} = 0.17 μM), axitinib (PDB: 4AG8, IC_{50} = 0.0002 μM), AAL993 (PDB: 3WZE, IC_{50} = 0.023 μM), cabozantinib (PDB: 5L2Q, IC_{50} = 0.035 μM), pazopanib (PDB: 2XIR, IC_{50} = 0.03 μM), sunitinib (PDB: 4AGD, IC_{50} = 0.04 μM), compound **XIV** reported in the literature (IC_{50} = 0.087 μM), and compound **14** from the present study (IC_{50} = 0.55 μM). All compounds were docked to VEGFR-2 (PDB: 3WZE) using the same docking protocol applied in the main study.

The resulting docking scores ranged from −10.20 to −8.32 kcal/mol, with an average value of −9.16 ± 0.66 kcal/mol. This range corresponds well with the docking scores typically observed for known VEGFR-2 inhibitors. Importantly, the docking scores showed qualitative agreement with the experimentally reported potencies. For example, the highly potent inhibitor axitinib (IC_{50} = 0.0002 μM) produced one of the most favorable docking scores (−10.12 kcal/mol), supporting the ability of the docking protocol to reasonably rank ligands according to their relative binding affinities.

Sorafenib, the clinically approved VEGFR-2 inhibitor used as a reference compound in this study, achieved a docking score of −10.20 kcal/mol, consistent with its well-established binding mode within the VEGFR-2 active site. Notably, compound **14** yielded a docking score of −8.50 kcal/mol, placing it within the docking score range observed for known VEGFR-2 inhibitors and comparable to compound **XIV** (−8.32 kcal/mol), a previously reported thiazolidinone-quinoline hybrid with experimentally confirmed VEGFR-2 inhibitory activity. These observations suggest that the docking protocol is capable of reproducing binding poses and relative scoring trends consistent with experimentally characterized VEGFR-2 inhibitors (**Table S12**).

B. Negative Control Set: Property-Matched DUD-E Decoys

Following the established methodology of the DUD-E database, twenty-five decoy compounds were selected with physicochemical properties similar to compound **14** (molecular weight: 520–560 Da; LogP: 4.5–5.0) but possessing distinct 2D topologies to minimize the

likelihood of true VEGFR-2 binding. These property-matched decoys yielded docking scores ranging from −6.12 to −7.50 kcal/mol, with an average value of −6.81 ± 0.36 kcal/mol. Importantly, all decoy compounds produced docking scores substantially weaker than those observed for the validated VEGFR-2 inhibitors presented in the positive control set. The lowest-scoring decoy (−7.50 kcal/mol) remained notably higher than the docking scores obtained for the known inhibitors (**Table S13**). This observation indicates that molecules with similar physicochemical properties but unrelated topologies do not reproduce docking scores comparable to those of experimentally validated VEGFR-2 inhibitors.

To further evaluate the specificity of the docking protocol, an additional negative control set composed of structurally unrelated molecules was examined. Fifteen compounds with no reported VEGFR-2 inhibitory activity were selected from diverse chemical classes, including NSAIDs (ibuprofen, aspirin), sugars (glucose), nucleotides (ATP), steroids (cholesterol), antibiotics (amoxicillin, penicillin G), vitamins (vitamin C, folic acid), and other therapeutics (metformin, omeprazole, caffeine, paracetamol, dopamine, and estradiol). These molecules were chosen to evaluate whether the docking workflow might generate false positive predictions for compounds lacking structural similarity to kinase inhibitors.

The resulting docking scores ranged from −5.41 to −8.51 kcal/mol, with an average value of −6.19 ± 0.64 kcal/mol. Fourteen out of fifteen compounds (93.3%) produced docking scores weaker than the score range observed for validated VEGFR-2 inhibitors, indicating a strong separation between known binders and unrelated molecules. Only one compound exhibited a borderline docking score slightly below this range (**Table S14**), which may reflect nonspecific hydrophobic contacts occasionally observed in docking simulations.

C- Integrated statistical analysis comparing docking scores of positive and negative control compounds

Combining all negative control categories (n = 40 compounds) and comparing them with the positive control set (n = 8 compounds) revealed a strong separation between the two score distributions. Positive controls exhibited docking scores ranging from −10.20 to −8.32 kcal/mol (mean: −9.16 ± 0.66 kcal/mol; median: −9.01 kcal/mol), whereas negative controls ranged from −8.51 to −5.41 kcal/mol (mean: −6.63 ± 0.56 kcal/mol; median: −6.75 kcal/mol). All eight positive controls (100%) achieved docking scores ≤ −8.3 kcal/mol, while 39 out of 40 negative control

compounds (97.5%) produced docking scores above this threshold. Statistical analysis comparing the two distributions yielded $p < 0.001$, confirming a highly significant difference between the docking scores of experimentally validated VEGFR-2 inhibitors and negative control compounds with only minimal overlap between the two datasets (**Table S15**).

D. Molecular Docking Analysis of Compound 14 against Viral Enzymes

The binding behavior of compound **14** was further investigated against a panel of viral enzymes, including HIV-1 protease, hepatitis virus polymerase, and SARS-CoV-2 main protease (Mpro), to assess its potential antiviral activity. The docking results demonstrated that compound **14** exhibits favorable binding affinities toward all three targets, with docking scores of −8.50 kcal/mol for HIV-1 protease, −8.60 kcal/mol for hepatitis virus polymerase, and −8.40 kcal/mol for SARS-CoV-2 Mpro (**Table S16**, **Figure S50).**

Within the HIV-1 protease active site, compound **14** did not form hydrogen bond interactions; instead, binding was predominantly stabilized through extensive hydrophobic and π–alkyl interactions involving residues **Ile50**, **Ile84**, **Ile54**, **Pro81**, **Ile47**, and **Ala28**. A total of nine hydrophobic contacts were observed, indicating that nonpolar interactions play a dominant role in stabilizing the ligand protease complex (**Figure S50 a-c**; **Table S16**).

Docking into the hepatitis virus polymerase revealed a more diverse interaction profile. Compound **14** formed a hydrogen bond with Glu885 (≈2.23 Å), serving as a key anchoring interaction within the polymerase binding site. This interaction was further supported by multiple hydrophobic π–alkyl contacts with residues such as **Lys534**, **Ile468**, **Tyr526**, and **Lys1069**, in addition to carbon hydrogen bond interactions involving **Thr530** and **Ile533**, collectively stabilizing the ligand within the catalytic cavity (**Figure S50 d-f**; **Table S16**).

In the case of SARS-CoV-2 Mpro, compound **14** adopted a favorable binding orientation within the substrate-binding pocket. A key hydrogen bond with **Glu166** (≈2.23 Å) was identified, accompanied by stabilizing hydrophobic and π-mediated interactions with residues including **Lys137**, **Leu141**, and **Val171**, supporting the formation of a stable ligand–enzyme complex (**Figure S50 g-i**; **Table S16**).

Taken together, the docking results indicate that compound **14** is capable of forming stable interactions with multiple viral enzymes through a balanced combination of hydrogen bonding and

hydrophobic contacts. The comparable binding affinities and conserved interaction features observed across HIV-1 protease, hepatitis virus polymerase, and SARS-CoV-2 Mpro highlight the structural adaptability of compound **14** toward diverse viral enzymatic pockets and support its potential as a multi-target antiviral scaffold.

3. Experimental Section

3.1. Chemistry

All reagents and solvents used in the synthesis were purchased from Sigma Aldrich Chemical Co. (St. Louis, MO, USA) and other standard commercial suppliers and were used without further purification unless otherwise stated. All solvents were dried prior to use following standard laboratory procedures. Melting points were determined using a Gallenkamp melting point apparatus (Gallenkamp, London, UK) and are reported as uncorrected values. Infrared (IR) spectra (KBr disks) were recorded using a Thermo Scientific Nicolet iS10 FTIR spectrometer (Thermo Fisher Scientific, Waltham, MA, USA) at the Faculty of Science, Menoufia University, with additional IR measurements performed at the Faculty of Science, Al-Azhar University, Cairo, Egypt. Proton and carbon nuclear magnetic resonance (^{1}H and ^{13}C NMR) spectra were recorded in DMSO-d_6 using a Bruker NEO NMR spectrometer (400 MHz for ^{1}H and 100 MHz for ^{13}C; Bruker BioSpin GmbH, Rheinstetten, Germany). Chemical shifts (δ) are reported in parts per million (ppm) relative to tetramethylsilane (TMS) as an internal standard, and coupling constants (J) are expressed in Hertz (Hz). Mass spectrometric analyses were carried out at the Faculty of Science, Al-Azhar University, Egypt, using a Thermo Scientific GC/MS ISQ LT system (Thermo Fisher Scientific, Waltham, MA, USA), with data processed using Xcalibur software (version 2.2, Thermo Fisher Scientific, Waltham, MA, USA). Elemental analyses (C, H, and N) were performed using a PerkinElmer 2400 elemental analyzer (PerkinElmer Inc., Waltham, MA, USA) at the Microanalytical Unit, Faculty of Science, Cairo University, Egypt. Reaction progress and compound purity were monitored by thin-layer chromatography (TLC) on aluminum-backed plates precoated with silica gel 60 F_{254} (Macherey–Nagel GmbH & Co. KG, Düren, Germany), and spots were visualized under ultraviolet light.

3.2. Biological activity

3.2.1. Antiproliferative Activity

The antiproliferative activity of the synthesized derivatives (**1**–**14**) was assessed against three human cancer cell lines, including liver carcinoma (HepG-2), colorectal carcinoma (HCT-116), and breast carcinoma (MCF-7), along with the normal human diploid fibroblast cell line (WI-38), using the MTT colorimetric assay. Doxorubicin and sorafenib were employed as standard reference drugs for comparison. Exponentially growing cells were seeded in 96-well plates at a density of 1×10^4 cells per well and incubated for 24 h at 37 °C in a humidified atmosphere containing 5% CO_2 to ensure proper cell adhesion and stabilization. The culture medium was subsequently replaced with fresh medium containing serial two-fold dilutions of the tested compounds, with concentrations ranging from 100 to 1.56 μM (100, 50, 25, 12.5, 6.25, 3.125, and 1.56 μM). Control wells were treated with compound-free medium under identical conditions. All experiments were conducted in triplicate. Following treatment, the cells were further incubated for 48 h. The medium was then carefully aspirated, and 20 μL of MTT solution (5 mg/mL in phosphate-buffered saline (PBS); Sigma–Aldrich, St. Louis, MO, USA) was added to each well, followed by incubation for an additional 4 h to allow the formation of formazan crystals. The formed crystals were dissolved in 150 μL of dimethyl sulfoxide (DMSO; Sigma–Aldrich, St. Louis, MO, USA), and the absorbance was measured at 570 nm using a microplate reader (Tecan Infinite® 200 PRO, Tecan Group Ltd., Männedorf, Switzerland). Cell viability was expressed as a percentage relative to untreated control cells. The half-maximal inhibitory concentration (IC_{50}) values were calculated from dose–response curves using nonlinear regression analysis with GraphPad Prism software (version 9.0, GraphPad Software, San Diego, CA, USA). The antiproliferative activity of the tested derivatives was compared with that of doxorubicin and sorafenib. The human cancer cell lines MCF-7, HCT-116, and HepG-2, along with the normal human fibroblast WI-38 cell line, were obtained from certified local suppliers in Egypt. All cell lines were authenticated prior to use and confirmed to be free from mycoplasma contamination. All biological experiments were conducted under standardized sterile conditions in the Research Laboratory, Faculty of Pharmacy, Mansoura University, Mansoura, Egypt.

3.2.2. In vitro enzyme inhibitory assay against VEGFR-2

The inhibitory activity of compound **14** against VEGFR-2 was evaluated using a VEGFR-2 (KDR) Kinase Assay Kit (BPS Bioscience, USA; Catalog No. 40325), following the manufacturer's protocol. The assay was performed in a 96-well plate format using purified

recombinant VEGFR-2 enzyme and a poly(Glu:Tyr) (4:1) substrate, with kinase activity quantified via the Kinase-Glo® MAX luminescent detection system. Briefly, reaction mixtures containing kinase buffer, ATP (500 μM), substrate, and test compound at various concentrations were prepared in duplicate. The enzymatic reaction was initiated by the addition of VEGFR-2 enzyme (approximately 1 ng/μL) and incubated at 30 °C for 45 min. Following incubation, Kinase-Glo® MAX reagent was added to each well, and luminescence was measured after 15 min using a microplate reader. The luminescence signal, inversely proportional to kinase activity, was used to calculate percentage inhibition. Sorafenib was employed as a reference VEGFR-2 inhibitor under identical experimental conditions. IC_{50} values were determined from dose–response curves generated using Excel software.

3.2.3. Wound healing assay

The anti-migratory activity of the tested compound **14** was evaluated using a wound healing (scratch) assay. Briefly, cancer cells were seeded in six-well plates at a density of 8×10^5 cells per well and allowed to grow until a confluent monolayer was formed. A linear scratch was generated across the cell monolayer using a sterile 200 μL pipette tip, followed by gentle washing with sterile PBS to remove detached cells. Fresh culture medium was then added with or without the tested compound.

Images of the wounded area were captured immediately after scratch formation (0 h) using an inverted microscope. After incubation for 24 h, the same wound areas were re-imaged to assess cell migration. The extent of wound closure was quantified by measuring the remaining gap area relative to the initial wound width, and results were expressed as percentage wound closure.

3.2.4. Cell Cycle Arrest and Apoptosis of Compound 14

Cell cycle distribution and apoptosis induction were analyzed by flow cytometry in HepG-2 cells following treatment with compound 14. Cells were seeded at a density of 8×10^4 cells per well and incubated overnight at 37 °C in a humidified atmosphere containing 5% CO_2. After 24 h treatment, cells were harvested and prepared for analysis.

For cell cycle analysis, cells were fixed in ice-cold 70% ethanol, stained with propidium iodide (PI) solution containing RNase, and incubated in the dark. DNA content was analyzed using

a Gallios flow cytometer (Beckman Coulter), and cell cycle phase distribution (G_0/G_1, S, and G_2/M) was quantified using Kaluza software v1.2.

Apoptosis was assessed using an Annexin V-FITC/PI apoptosis detection kit (BioVision, USA) according to the manufacturer's instructions. Cells were stained in binding buffer and analyzed by flow cytometry within 1 h. Early apoptotic, late apoptotic, and necrotic populations were identified based on Annexin V and PI fluorescence. Results were expressed as percentages of cells in each category.

3.2.5. Estimation the levels of Caspase-3

Active caspase-3 levels were quantified using a human caspase-3 (active) ELISA kit (Invitrogen, Thermo Fisher Scientific, USA; Catalog No. KHO1091) according to the manufacturer's protocol. HepG-2 cells were treated with compound 14 for 24 h, harvested, washed twice with ice-cold PBS, and lysed using a protease inhibitor–supplemented cell extraction buffer. Cell lysates were clarified by centrifugation at 13,000 rpm for 10 min at 4 °C, and the supernatants were collected for analysis.

Briefly, 100 µL of standards or appropriately diluted cell lysates were added to antibody-coated microplate wells and incubated for 2 h at room temperature. After washing, wells were incubated sequentially with a caspase-3 detection antibody and HRP-conjugated secondary antibody, followed by addition of tetramethylbenzidine (TMB) substrate. The reaction was stopped using stop solution, and absorbance was measured at 450 nm using a microplate reader. Active caspase-3 concentrations were calculated from a standard calibration curve generated using four-parameter logistic regression. Results were expressed as pg/mL and normalized to control values to calculate fold change. All experiments were performed in triplicate, and data are presented as mean ± SD.

3.3. In silico analyses

3.3.2. Molecular docking study

Molecular docking simulations were performed to investigate the binding interactions of compound **14** with selected biological targets, including VEGFR-2, HIV-1 protease, hepatitis virus polymerase, and SARS-CoV-2 main protease (Mpro). The three-dimensional crystal structures of

the target proteins were retrieved from the Protein Data Bank (PDB), and the corresponding structural information, including PDB identifiers and active-site coordinates, is summarized in **Table S10**.

Protein structures were prepared using PyMOL by removing co-crystallized ligands, solvent molecules, and ions. Polar hydrogen atoms were subsequently added, and the prepared receptor structures were converted to PDBQT format using AutoDock Tools. The ligand structure of compound **14** was geometry-optimized and converted to mol2 format using Open Babel prior to docking calculations.

Molecular docking simulations were carried out using AutoDock Vina. The docking grid boxes were centered on the reported active-site coordinates of each target protein, which were defined based on the positions of the co-crystallized ligands present in the corresponding PDB structures. Grid box dimensions were selected to adequately encompass the catalytic binding pocket and allow sufficient conformational sampling of the ligand.

Docking calculations were performed using the default AutoDock Vina parameters with an exhaustiveness value of 8. For each ligand, multiple binding poses were generated, and the resulting protein–ligand complexes were ranked according to their predicted binding affinity scores. The top-ranked docking conformations were selected for subsequent structural analysis.

Sorafenib, a clinically approved VEGFR-2 inhibitor, and doxorubicin were docked under identical conditions and used as reference ligands for comparative analysis. Post-docking analysis and visualization of protein–ligand interactions were carried out using BIOVIA Discovery Studio Visualizer (version 4.5), allowing identification of key hydrogen-bonding interactions, hydrophobic contacts, and π-mediated interactions within the binding sites.

3.3.3. Molecular Dynamic Simulations

Molecular dynamics (MD) simulations were performed to further investigate the dynamic stability and binding behavior of compound **14** in complex with VEGFR-2 and to validate the molecular docking results. All MD simulations were carried out using the GROMACS 2018 simulation package. The protein structure of VEGFR-2 was prepared by removing crystallographic water molecules and heteroatoms, followed by the addition of missing hydrogen atoms. Protein

topology files were generated using the CHARMM36 force field, which is widely validated for protein systems.

The topology and force field parameters for compound **14** were generated using the GEFF/GEOM (Geoff) server, and the ligand was subjected to appropriate positional restraints during the equilibration stages. The protein–ligand complex was placed in a triclinic simulation box with a minimum distance of 1.0 nm between the protein surface and the box boundaries. The system was solvated using the TIP3P water model, and appropriate counterions (Na^+/Cl^-) were added to neutralize the system and mimic physiological ionic strength.

Energy minimization was performed using the steepest descent algorithm until convergence was achieved, ensuring the removal of steric clashes and unfavorable contacts. Subsequently, the system was equilibrated in two sequential phases: (i) NVT ensemble (constant number of particles, volume, and temperature) for 1,000 ps, followed by (ii) NPT ensemble (constant number of particles, pressure, and temperature) for 1,000 ps, both conducted at a temperature of 300 K and a pressure of 1.0 bar. Temperature coupling was maintained using the V-rescale thermostat, while pressure was controlled using the Parrinello–Rahman barostat. All covalent bonds involving hydrogen atoms were constrained using the LINCS algorithm, allowing a time step of 2 fs.

Following equilibration, a 100 ns production MD simulation was performed under periodic boundary conditions without positional restraints. Long-range electrostatic interactions were calculated using the Particle Mesh Ewald (PME) method, while van der Waals interactions were treated using a cut-off distance of 1.0 nm.

Trajectory analysis was conducted using built-in GROMACS tools. The root mean square deviation (RMSD) of the protein backbone atoms was calculated to assess overall structural stability over the simulation time. Root mean square fluctuation (RMSF) analysis was performed to evaluate residue-level flexibility. The radius of gyration (Rg) was monitored to examine protein compactness and folding behavior, while the solvent-accessible surface area (SASA) was calculated to assess changes in solvent exposure upon ligand binding. Additionally, intramolecular hydrogen bonds within the protein and intermolecular hydrogen bonds between VEGFR-2 and compound **14** were analyzed throughout the simulation to evaluate interaction persistence and

complex stability. All MD simulation analyses were performed following established protocols for protein–ligand systems and were consistent with previously reported molecular dynamics studies.

3.3.4. Quantum Chemical Calculations

Density Functional Theory (DFT) computations were performed using the B3LYP functional, a hybrid exchange-correlation functional that combines the gradient-corrected correlation functional of Lee, Yang, and Parr (LYP) with Becke's three-parameter exchange functional. By addressing integration concerns, this method has advantages over pure DFT techniques.

Gaussian 09 was used to fully optimize all molecular geometries at the B3LYP/6-311G++(d,p) level of theory. The energy gap (ΔE), global electrophilicity (ω), softness (σ), electronegativity (χ), hardness (η), and ionization potential (I) were then determined using frontier molecular orbital (FMO) analysis.

Using the optimized geometries, calculations were carried out at the B3LYP/6-311G++(d,p) level of theory in order to visualize the molecular electrostatic potential (MEP). The MEP maps reveal information about the molecules' electrophilic and nucleophilic areas.

Tables

Table S1. The percentage cytotoxicity of the active compounds **1-14** on HepG-2, HCT-116, and MCF-7, and normal WI38 cell lines at different concentrations.

Average of Relative viability of cells (%)

Conc.(µM)	WI38	HCT116	HePG2	MCF7
		**DOX**		
100	7.8	7.1	6.3	6.2
50	14.5	13.9	11.2	10.9
25	21.2	18.7	14.1	14.3
12.5	34.0	31.4	28.3	26.9
6.25	50.3	47.9	45.8	41.5
3.125	64.9	60.5	57.6	58.4
1.56	87.4	73.8	71.2	69.1

SOR				
100	13.1	7.4	9.2	8.1
50	17.8	12.1	16.6	15.2
25	29.0	18.8	25.7	23.0
12.5	40.3	27.9	37.2	34.8
6.25	63.7	52.7	61.3	51.6
3.125	78.6	63.5	72.9	68.9
1.56	96.5	72.4	95.4	87.5
1				
100	30.4	38.5	41.3	38.3
50	42.6	54.3	49.7	50.7
25	53.5	69.2	65.5	62.4
12.5	66.7	88.4	78.6	78.9
6.25	87.8	100	92.4	96.5
3.125	99.3	100	100	100
1.56	100	100	100	100
2				
100	36.1	7.9	12.8	6.7
50	48.3	19.5	21.6	13.5
25	64.5	21.3	29.5	21.2
12.5	77.2	38.9	40.7	32.8
6.25	93.6	57.4	67.2	48.6
3.125	100	85.6	81.4	63.3
1.56	100	98.8	98.3	86.1
3				
100	52.3	51.6	48.6	47.9
50	69.5	62.8	65.4	60.4
25	78.6	78.2	68.7	72.8
12.5	96.4	90.4	92.3	87.3
6.25	100	100	100	98.5
3.125	100	100	100	100
1.56	100	100	100	100
4				
100	38.7	18.5	20.4	9.7
50	51.8	26.1	31.8	17.8
25	65.3	33.7	42.6	26.4
12.5	79.5	40.6	54.3	32.9
6.25	97.2	68.4	71.9	62.3

3.125	100	85.2	92.2	71.6
1.56	100	100	100	91.2
5				
100	43.9	35.6	35.2	28.6
50	55.4	48.2	49.1	41.7
25	62.7	60.4	56.6	53.2
12.5	84.2	72.5	69.9	69.5
6.25	95.6	89.3	83.7	81.9
3.125	100	100	98.4	100
1.56	100	100	100	100
6				
100	36.5	33.5	31.4	24.8
50	49.3	45.2	41.8	39.2
25	60.4	56.0	52.6	51.6
12.5	74.6	69.3	73.3	68.4
6.25	87.2	91.4	88.5	79.1
3.125	100	100	99.2	97.5
1.56	100	100	100	100
7				
100	45.5	54.3	50.7	52.4
50	58.1	71.6	63.2	69.6
25	70.4	85.4	80.4	78.3
12.5	83.2	98.2	93.6	96.5
6.25	98.0	100	100	100
3.125	100	100	100	100
1.56	100	100	100	100
8				
100	30.4	48.2	47.3	47.6
50	41.6	60.4	58.6	52.5
25	53.2	76.3	72.4	82.7
12.5	65.7	88.1	84.7	97.3
6.25	79.3	98.7	98.2	100
3.125	98.1	100	100	100
1.56	100	100	100	100
9				
100	21.4	37.6	43.6	36.2
50	28.9	53.4	52.2	48.3
25	41.5	62.5	62.4	63.5

12.5	50.7	74.2	78.3	75.7
6.25	72.3	96.7	99.5	96.1
3.125	91.6	100	100	100
1.56	100	100	100	100
10				
100	51.6	38.6	34.2	33.5
50	70.3	49.1	49.3	45.2
25	86.1	62.5	60.7	58.4
12.5	95.4	78.3	74.4	69.6
6.25	100	96.4	96.1	90.3
3.125	100	100	100	100
1.56	100	100	100	100
11				
100	25.3	47.3	42.6	36.4
50	36.5	55.6	58.4	55.6
25	50.2	76.4	69.3	68.3
12.5	64.7	89.7	84.7	78.0
6.25	80.1	100	99.2	93.5
3.125	96.4	100	100	100
1.56	100	100	100	100
12				
100	48.6	45.7	38.2	39.5
50	62.0	56.4	54.7	54.7
25	75.4	71.5	71.5	72.4
12.5	92.3	83.2	92.3	93.6
6.25	100	96.1	100	100
3.125	100	100	100	100
1.56	100	100	100	100
13				
100	41.6	29.7	23.7	26.7
50	53.2	40.9	31.9	37.4
25	64.3	50.8	43.6	50.6
12.5	77.1	65.5	55.8	58.5
6.25	100	78.2	69.6	75.3
3.125	100	98.3	88.4	93.9
1.56	100	100	100	100
14				
100	42.8	24.2	8.6	20.4

**50**	**57.2**	**35.4**	**16.9**	**27.8**
**25**	**69.4**	**46.3**	**24.5**	**38.6**
**12.5**	**81.3**	**57.5**	**32.8**	**52.9**
**6.25**	**95.1**	**71.6**	**55.4**	**73.7**
**3.125**	**100**	**90.8**	**71.3**	**91.5**
**1.56**	**100**	**100**	**89.7**	**100**

Table S2. Detailed results of the VEGFR-2 enzyme inhibitory assay of -based derivative **14**.

VEGFR2

code	IC50	conc	log	%inh	ΔT	ΔRFU	slope	K.Activity	EC	RFU1	RFU2	RFU3
						[redacted]	[redacted]	[redacted]				
s14		100	2	95.2	30	2965.9	2073	5.7229	120	2951	2983	2964
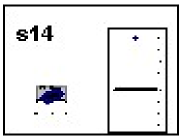		10	1	84.7	30	9490.6	2073	18.313	120	9443	9544	9485
		1	0	58.4	30	25876	2073	49.929	120	25746	26022	25859
		0.1	-1	37.4	30	38958	2073	75.171	120	38762	39178	38933
		0.01	-2	24.1	30	47208	2073	91.091	120	46971	47475	47178
EC				0	30	62199	2073	120	120	61887	62551	62159
	[redacted]		[redacted]		[redacted]	[redacted]		[redacted]				

code	IC50	conc	log	%inh	ΔT	ΔRFU	slope	K.Activity	EC	RFU1	RFU2	RFU3
Sorafenib		100	2	97	30	1883.5	2073	3.6343	120	1874	1894	1882
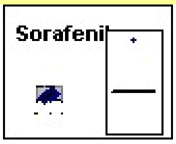		10	1	87.5	30	7764	2073	14.981	120	7725	7808	7759
		1	0	75	30	15540	2073	29.986	120	15462	15628	15530
		0.1	-1	53.5	30	28936	2073	55.835	120	28791	29100	28918
		0.01	-2	30.1	30	43473	2073	83.885	120	43255	43719	43445
EC				0	30	62199	2073	120	120	61887	62551	62159
	[redacted]		[redacted]		[redacted]	[redacted]		[redacted]				

Table S3. Detailed results of wound healing assay of **14** within HePG-2

	% Closure	Total area	Migrated cells area	T	Length	L.of migration	L1	L2	L3
				72h	mm	ΔL			
**s14/HepG2**	**63.704**	0.81	0.516		0.9	0.29	0.28	0.29	0.29
**cont.HepG2**	**97.037**	0.81	0.786		0.9	0.44	0.43	0.44	0.44

Table S4. Cell cycle analysis after 48 h incubation with compound **14** compared with untreated HepG-2 cells.

**ser**	**Sample**		**DNA content**				
	**code**	**IC50 uM**	**%G0-G1**	**%S**	**%G2/M**	**Comment**	
1	**s14/HepG2**	**---**	**72.86**	**22.32**	**4.82**	Cell cycle arrest@G1	
2	**cont.HepG2**	**---**	**49.61**	**35.44**	**14.95**	---	

Table S5. Apoptosis induction analysis within HepG-2 cells treated with compound **14** compared with untreated HepG-2 cells.

**s**	**code**	**conc**	**Apoptosis**			**Necrosis**
			Total	Early	Late	
1	**s14/HepG2**	**---**	**36.04**	**22.18**	**9.84**	**4.02**
2	**cont.HepG2**		**2.86**	**0.88**	**0.23**	**1.75**

Table S6. Raw absorbance values obtained directly from the ELISA plate reader for caspase-3 assay

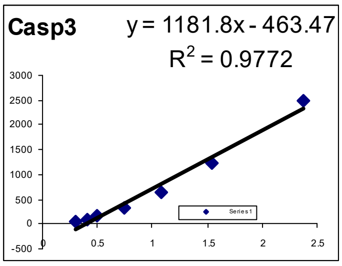

STANDARDS	pg/ml
St.1	2500
St.2	1250
St.3	625
St.4	313
St.5	156
St.6	78
St.7	39

Plate map

	1	2	3	4	5	6	7	8	9	10	11	12
A	st 1	**s14**	--	--	--	--	--	--	--	--	--	--
B	st 2	**s14**	--	--	--	--	--	--	--	--	--	--
C	st 3	**cont**	--	--	--	--	--	--	--	--	--	--
D	st 4	**cont**	--	--	--	--	--	--	--	--	--	--
E	st 5	**--**	--	--	--	--	--	--	--	--	--	--
F	st 6	**--**	--	--	--	--	--	--	--	--	--	--
G	st 7	--	--	--	--	--	--	--	--	--	--	--
H	B	--	--	--	--	--	--	--	--	--	--	--

Samples OD results

	1	2	3	4	5	6	7	8	9	10	11	12
A	2.376	**0.829**	0	0	0	0	0	0	0	0	0	0
B	1.551	**0.841**	0	0	0	0	0	0	0	0	0	0
C	1.079	**0.465**	0	0	0	0	0	0	0	0	0	0
D	0.737	**0.493**	0	0	0	0	0	0	0	0	0	0
E	0.488	**0**	0	0	0	0	0	0	0	0	0	0

F	0.415	**0**	0	0	0	0	0	0	0	0	0	0
G	0.297	0	0	0	0	0	0	0	0	0	0	0
H	0.066	0	0	0	0	0	0	0	0	0	0	0

Table S7. Calculated caspase-3 concentrations derived from the standard calibration curve for HepG-2 cell samples.

**STANDARDS**	OD	ng/ml	**Slope**	**Intercept**	**R2**
St.1	2.376	2500	0.000826904	0.405818227	0.9772
St.2	1.551	1250			
St.3	1.079	625			
St.4	0.737	313			
St.5	0.488	156			
St.6	0.415	78			
St.7	0.297	39			
	0.066				

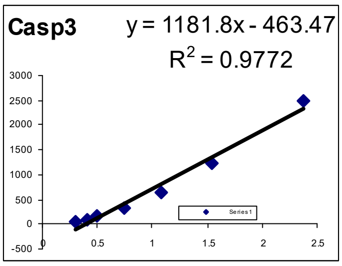

Plate map **HepG2**

	**1**	**2**
A	st 1	**s14**
B	st 2	**s14**
C	st 3	**cont**
D	st 4	**cont**
E	st 5	--
F	st 6	--

G	st 7	--
H	B	--

Samples ODs

	**1**	**2**
A	2.376	**0.829**
B	1.551	**0.841**
C	1.079	**0.465**
D	0.737	**0.493**
E	0.488	0
F	0.415	0
G	0.297	0
H	0.066	0

results

	**1**	**2**
A	2393.789	522.956
B	1396.092	537.468
C	825.288	82.760
D	411.697	116.621
E	110.574	-479.579
F	22.293	-479.579
G	-120.408	-479.579
H	-399.763	-479.579

Table S8. Predicted pharmacokinetic, physicochemical, metabolism, excretion, and absorption distribution related properties of compound **14** in comparison with doxorubicin. The table summarizes comprehensive ADMET descriptors, including molecular weight, lipophilicity, aqueous solubility, topological polar surface area, hydrogen-bond donors and acceptors, metabolic enzyme interactions, clearance, solubility and permeability parameters, and medicinal chemistry indices.

Id	ID	14	sorafenib	Id	ID	14	sorafenib
Physicochemical Properties	MW	539.19	464.09	Metabolism	CYP1A2-inh	0.360	1.00
	Vol	531.50	420.87		CYP1A2-sub	0.002	0.52
	Dense	1.01	1.10		CYP2C19-inh	0.009	0.96
	nHA	7.00	7		CYP2C19-sub	0.000	0.00
	nHD	0.00	3		CYP2C9-inh	0.094	0.88
	TPSA	71.97	92.35		CYP2C9-sub	0.000	0.01
	nRot	9.00	9		CYP2D6-inh	0.041	0.00
	nRing	5.00	3		CYP2D6-sub	0.992	0.00
	MaxRing	10.00	6		CYP3A4-inh	0.931	0.06
	nHet	9.00	11		CYP3A4-sub	0.335	1.00
	fChar	0.00	0	Excretion	CL (Clearance)	0.618	0.02
	nRig	29.00	20		T12	0.618	0.37
	Flex	0.31	0.45	Toxicity	hERG Blockers	0.803	0.39
	nStereo	2.00	0		H-HT	4.386	0.11
Solubility	LogS	-4.840	-6.41		DILI	0.126	1.00
	LogD	3.773	3.83		AMESToxicity	0.036	0.00
	LogP	4.722	4.68		Oral Toxicity	1.000	0.28
	ESOL Log S	-5.99	-3.91		FDAMDD	0.973	0.78
	Ali Log S	-7.28	-5.20		Skin Sens.	0.764	1.00
	Silicon-IT class	Moderately	Soluble		Carcinogenicity	0.729	0.78
drug-likeness	Lipinski Rule	Accepted	Accepted		Eye Corrosion	0.000	0.92
	Pfizer Rule	Rejected	Rejected		Eye Irritation	0.001	0.76
	Golden Triangle	Rejected	Accepted		Respiratory	0.998	0.34
Absorption	Pgp-inh	0.419	0.001	Toxicophore Rules	H-HT	0.405	0.63
	Pgp-sub	0.006	0.998		Neurotoxicity	0.951	0.97
	HIA	0.618	0.829		Ototoxicity	0.634	0.68
	F (20%)	0.016	0.055		Hematotoxicity	0.072	0.55
	F (30%)	0.010	0.209		Nephrotoxicity	0.112	0.61
	Caco-2	-5.012	-5.17		Genotoxicity	0.783	0.32
	MDCK	-4.537	-4.87		RPMI-8226	0.017	0.08
Distribution	BBB	0.319	0.015	Medicinal Chemistry	A549	0.187	0.20
	PPB	95.913	99.33		QED	0.357	0.461
	VDss	0.289	1.177		Synth	3.00	2
	Fu	3.037	0.25		Fsp3	0.464	0.095

Table S9. *In silico* toxicity risk and drug-likeness prediction of compound **14** in comparison with Sorafenib. The table reports predicted mutagenicity, tumorigenicity, irritation, reproductive toxicity, and composite drug-likeness and drug score parameters.

No	Ligand	Toxicity risks				Physicochemical properties					
		Mutagenic	Tumorigenic	Irritant	Reproductive	CLogP	Solubility	Molecular Weight	TPSA	Drug likeness	Drug score
1	**14**	(+)	(-)	(-)	(-)	-5.64	-5.64	539.0	125.5	1.09	0.12
2	**Sorafenib**	(-)	(-)	(-)	(-)	4.14	-6.69	464.2	92.35	-4.20	0.20

Table S10. Target proteins used for molecular docking, including PDB identifiers, crystal structure resolution, active-site coordinates, co-crystallized ligands, and literature references.

No	Protein Targets	PDB ID	Resolution (Å)	Active site coordinates:			Reference Ligands
				X	Y	Z	
**1**	VEGFR	**3WZE**	1.90 Å	19.51	24.05	29.45	doxorubicin
**2**	VEGFR	**4ASD**	2.40 Å	-8.7	3.4	21.6	Sorafenib
**3**	HIV-1 Protease	**3NU3**	1.02 Å	14.71	21.00	10.70	-
**4**	Hepatitis virus Polymerase	**7LUF**	3.50 Å	20.10	145.1	28.00	-
**5**	SARS-CoV-2 Mpro	**7ZB7**	1.63 Å	-7.90	1.10	15.02	-

Table S11. Key molecular interactions of compound **14**, sorafenib, and doxorubicin within the VEGFR-2 active site.

NO	Protein	Ligand	3D Structure	Hydrophilic Interactions		Hydrophobic Contacts		No. of H-Bonds	No. of Total Bonds	affinity kcal mol-1
				Residue (H- Bond)	Length	Residue (Bond type)	Length			
1	VEGFR-2	**14**	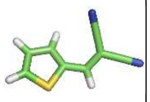	Glu885, (H- Bond)	2.23	Pro1068, (Pi-alkyl)<br>Arg1027, (Pi-alkyl)<br>Leu1049, (Pi-alkyl)<br>Ala881, (Pi-alkyl)<br>Leu889, (Pi-alkyl)<br>Ile888, (Pi-alkyl)<br>His816, (Pi-alkyl)<br>Glu885, (Sulfur)<br>Asp1046, (Pi-cation)<br>Arg1066, (Carbon H-Bond)	5.41<br>4.77<br>4.20<br>4.17<br>4.85<br>4.63<br>4.18<br>4.25<br>3.55<br>3.73	**1**	**11**	**-8.50**
2		**sorafenib**	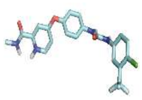	Cys919, (H- Bond)<br>Cys919, (H- Bond)	2.38<br>2.05	Phe918, (Pi-alkyl)<br>Leu840, (Pi-alkyl)<br>Ala866, (Pi-alkyl)<br>Val848, (Pi-alkyl)<br>Val899, (Pi-alkyl)<br>Lys868, (Pi-alkyl)<br>Cys1045, (sulfur)<br>Leu889, (Pi-sigma)<br>Val916, (Pi-sigma)<br>His1026, (Carbon H-Bond)<br>Lys920, (Carbon H-Bond)<br>Glu917, (Carbon H-Bond)	4.74<br>5.02<br>5.04<br>4.69<br>5.35<br>5.18<br>5.18<br>3.66<br>3.73<br>3.23<br>3.55<br>3.77	**2**	**14**	**-10.20**

3		**Doxo.**	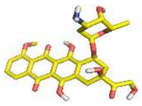	Ala881,<br>(H- Bond)<br>Asp1046,<br>(H- Bond)	2.59<br>2.68	Pro1068, (Pi-alkyl)<br>Pro1068, (Pi-alkyl)<br>Leu1067, (Pi-alkyl)<br>Tyr1082, (Pi-alkyl)<br>Ile888, (Pi-alkyl)<br>Asp1046, (Pi-alkyl)<br>Asp1028, (Pi-cation)<br>Asp1028, (Pi-cation)<br>Asp1046, (Pi-cation)<br>His816, (Carbon H-Bond)	5.10<br>4.23<br>4.75<br>4.97<br>3.72<br>3.96<br>4.86<br>4.51<br>4.51<br>3.58	**2**	**12**	**-8.40**

Table S12. Experimentally validated VEGFR-2 inhibitors used as positive controls with reported binding affinities and docking scores.

Compound	PDB ID	Experimental Affinity	Docking Score (kcal/mol)	Reference
Sorafenib	4ASD	IC50 = 0.17 µM	-10.20	This study
Axitinib	4AG8	IC50 = 0.0002 µM	-10.12	PDB 4AG8
AAL993	3WZE	IC50 = 0.023 µM	-9.45	PDB 3WZE
Cabozantinib	5L2Q	IC50 = 0.035 µM	-9.01	PDB 5L2Q
Pazopanib	2XIR	IC50 = 0.03 µM	-8.92	PDB 2XIR
Sunitinib	4AGD	IC50 = 0.04 µM	-8.76	PDB 4AGD
Compound **14**	-	IC50 = 0.55 µM	-8.50	This study
Compound **XIV** [115]	-	IC50 = 0.087 µM	-8.32	Reference [115]

Table S13. Category A negative controls: property-matched DUD-E decoy compounds with docking scores.

Compound Name/ID	MW Range	LogP Range	Source/Reference	Docking Score (kcal/mol)
DUD-E DECOY VGFR2_0001	520-560	4.5-5.0	DUD-E VEGFR-2 set	-7.12
DUD-E DECOY VGFR2_0002	520-560	4.5-5.0	DUD-E VEGFR-2 set	-7.20
DUD-E DECOY VGFR2_0003	520-560	4.5-5.0	DUD-E VEGFR-2 set	-7.30
DUD-E DECOY VGFR2_0004	520-560	4.5-5.0	DUD-E VEGFR-2 set	-7.21
DUD-E DECOY VGFR2_0005	520-560	4.5-5.0	DUD-E VEGFR-2 set	-6.91
DUD-E DECOY VGFR2_0006	520-560	4.5-5.0	DUD-E VEGFR-2 set	-6.50
DUD-E DECOY VGFR2_0007	520-560	4.5-5.0	DUD-E VEGFR-2 set	-6.99
DUD-E DECOY VGFR2_0008	520-560	4.5-5.0	DUD-E VEGFR-2 set	-6.90
DUD-E DECOY VGFR2_0009	520-560	4.5-5.0	DUD-E VEGFR-2 set	-7.10
DUD-E DECOY VGFR2_0010	520-560	4.5-5.0	DUD-E VEGFR-2 set	-7.20
DUD-E DECOY VGFR2_0011	520-560	4.5-5.0	DUD-E VEGFR-2 set	-7.50
DUD-E DECOY VGFR2_0012	520-560	4.5-5.0	DUD-E VEGFR-2 set	-6.31
DUD-E DECOY VGFR2_0013	520-560	4.5-5.0	DUD-E VEGFR-2 set	-7.45
DUD-E DECOY VGFR2_0014	520-560	4.5-5.0	DUD-E VEGFR-2 set	-6.45
DUD-E DECOY VGFR2_0015	520-560	4.5-5.0	DUD-E VEGFR-2 set	-7.35
DUD-E DECOY VGFR2_0016	520-560	4.5-5.0	DUD-E VEGFR-2 set	-6.80
DUD-E DECOY VGFR2_0017	520-560	4.5-5.0	DUD-E VEGFR-2 set	-7.45
DUD-E DECOY VGFR2_0018	520-560	4.5-5.0	DUD-E VEGFR-2 set	-6.50
DUD-E DECOY VGFR2_0019	520-560	4.5-5.0	DUD-E VEGFR-2 set	-6.75
DUD-E DECOY VGFR2_0020	520-560	4.5-5.0	DUD-E VEGFR-2 set	-6.12
DUD-E DECOY VGFR2_0021	520-560	4.5-5.0	DUD-E VEGFR-2 set	-7.00
DUD-E DECOY VGFR2_0022	520-560	4.5-5.0	DUD-E VEGFR-2 set	-6.88
DUD-E DECOY VGFR2_0023	520-560	4.5-5.0	DUD-E VEGFR-2 set	-6.75
DUD-E DECOY VGFR2_0024	520-560	4.5-5.0	DUD-E VEGFR-2 set	-6.84
DUD-E DECOY VGFR2_0025	520-560	4.5-5.0	DUD-E VEGFR-2 set	-6.55

Table S14. Category B negative controls: structurally unrelated molecules used to evaluate docking specificity.

Compound Name	CAS Number	MW	Class	Docking Score (kcal/mol)
Ibuprofen	15687-27-1	206.28	NSAID	-6.55
Glucose	50-99-7	180.16	Monosaccharide	-7.44
ATP	56-65-5	507.18	Nucleotide	-6.55
Cholesterol	57-88-5	386.65	Sterol	-8.51
Metformin	657-24-9	129.16	Antidiabetic	-7.10
Aspirin	50-78-2	180.16	NSAID	-6.20
Paracetamol	103-90-2	151.16	Analgesic	-5.80
Amoxicillin	26787-78-0	365.40	Antibiotic	-6.75
Omeprazole	73590-58-6	345.42	Proton pump inhibitor	-6.30
Caffeine	58-08-2	194.19	CNS stimulant	-5.90
Vitamin C	50-81-7	176.12	Vitamin	-5.50
Penicillin G	61-33-6	334.39	Antibiotic	-6.80
Dopamine	51-61-6	153.18	Neurotransmitter	-5.41
Estradiol	50-28-2	272.38	Hormone	-6.30
Folic acid	59-30-3	441.40	Vitamin B9	-5.50

Table S15. Integrated statistical analysis comparing docking scores of positive and negative control compounds.

Parameter	Positive Controls (n=8)	Negative Controls (n=40)
Docking score range (kcal/mol)	-10.20 to -8.32	-8.50 to -5.41
Mean docking score (kcal/mol)	-9.16 ± 0.66	-6.63 ± 0.56
Median docking score (kcal/mol)	-9.01	-6.75
Compounds ≤ -8.3 kcal/mol	8 (100%)	1 (2.5%)
Compounds > -8.3 kcal/mol	0 (0%)	39 (97.50%)

Table S16. Molecular docking interactions of compound **14** with selected viral enzymes.

NO	Compound	Proteins	Hydrophilic Interactions		Hydrophobic Contacts		No. of H-Bonds	No. of Total Bonds	affinity kcal mol-1
			Residue (H-Bond)	Length	Residue (Bond type)	Length			
1	14	HIV-1 Protease	-	-	Ile50, (Pi-alkyl)<br>Ile84, (Pi-alkyl)<br>Ile54, (Pi-alkyl)<br>Ile54, (Pi-alkyl)<br>Pro81, (Pi-alkyl)<br>Pro81, (Pi-alkyl)<br>Ile47, (Pi-alkyl)<br>Ile47, (Pi-alkyl)<br>Ala28, (Pi-alkyl)	5.13<br>5.04<br>4.60<br>4.61<br>4.74<br>4.67<br>5.24<br>4.79<br>4.73	0	9	-8.50
2		Hepatitis virus Polymerase	Glu885, (H-Bond)	2.23	Lys534, (Pi-alkyl)<br>Ile468, (Pi-alkyl)<br>Tyr526, (Pi-alkyl)<br>Lys1069, (Pi-alkyl)<br>Lys1069, (Pi-alkyl)<br>Thr530, (Carbon H-Bond)<br>Thr530, (Carbon H-Bond)<br>Ile533, (Carbon H-Bond)	4.69<br>4.76<br>4.85<br>4.40<br>4.61<br>3.54<br>3.62<br>3.43	1	9	-8.60
3		SARS-CoV-2 Mpro	Glu166, (H-Bond)	2.23	Lys137, (Pi-alkyl)<br>Leu141, (Pi-alkyl)<br>Leu141, (Pi-sigma)<br>Glu166, (Pi-cation)<br>Val171, (Carbon H-Bond)	3.91<br>4.68<br>3.73<br>3.69<br>3.63	1	6	-8.40

Table S17. Graphical representations of the frontier molecular orbitals (HOMO and LUMO) of the investigated compounds obtained from DFT calculations, illustrating the spatial distribution of electron density in the highest occupied and lowest unoccupied molecular orbitals.

	HOMO	LUMO
**1**	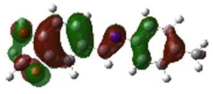	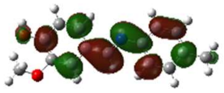
**2**	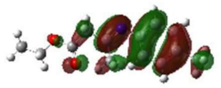	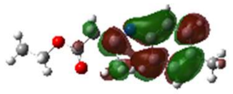
**3**	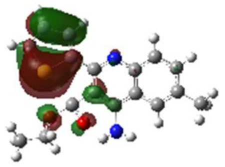	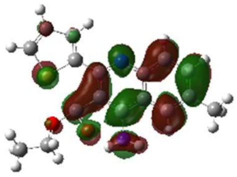
**4**	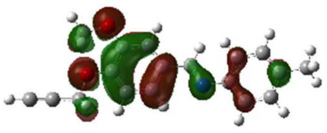	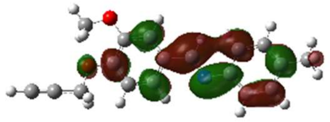
**5**	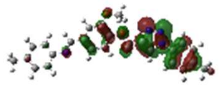	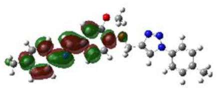

**6**	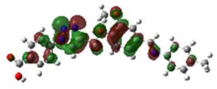	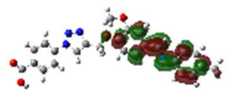
**7**	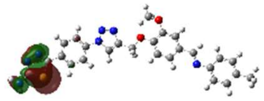	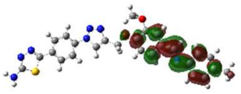
**8**	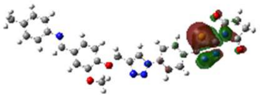	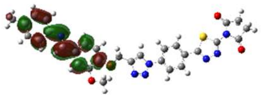
**9**	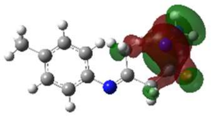	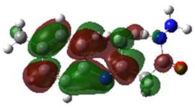
**10**	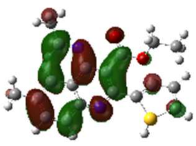	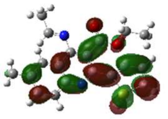

**11**	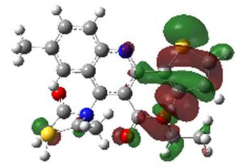	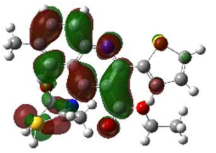
**12**	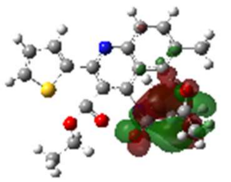	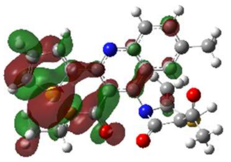
**13**	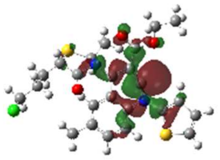	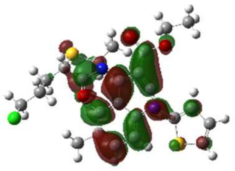
**14**	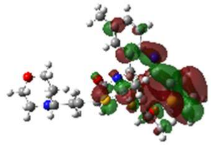	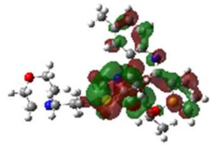

Table S18. Electron density surfaces of compounds **1–14** generated from total SCF density, showing the spatial distribution of electron density and supporting the HOMO–LUMO and ESP analyses.

1

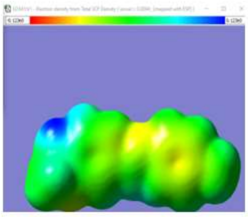

2

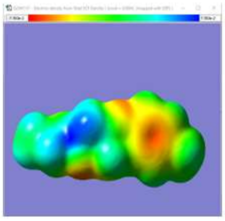

3

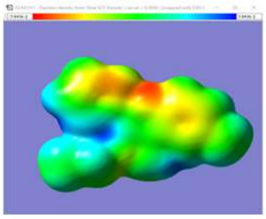

4

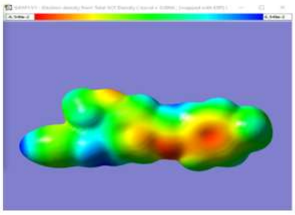

5

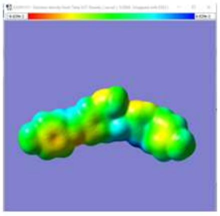

6

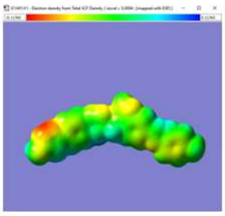

7

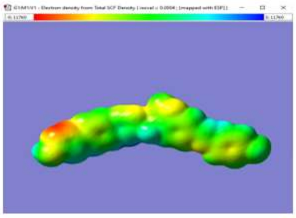

8

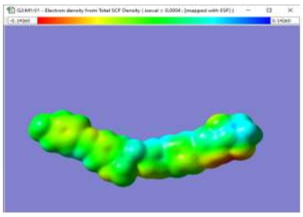

9

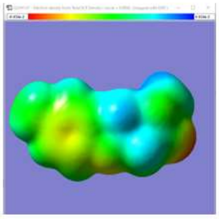

10

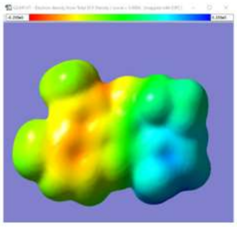

11

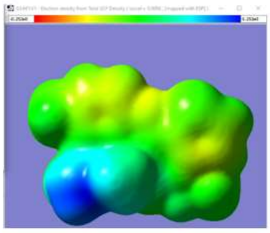

12

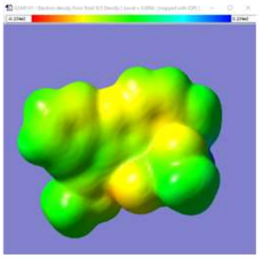

13

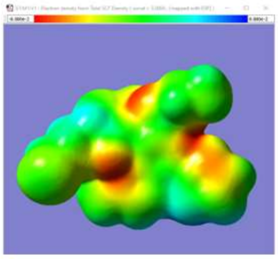

14

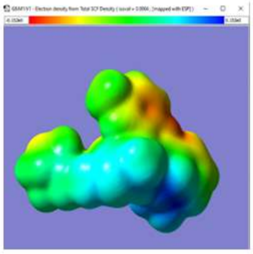

Copies of IR, mass, ^{1}H NMR and ^{13}C NMR of Compounds

Characterization of Compound 1:

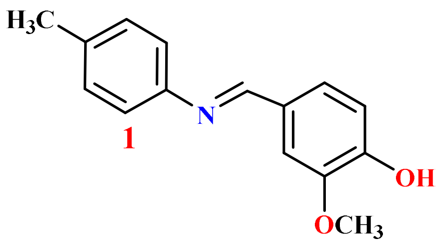

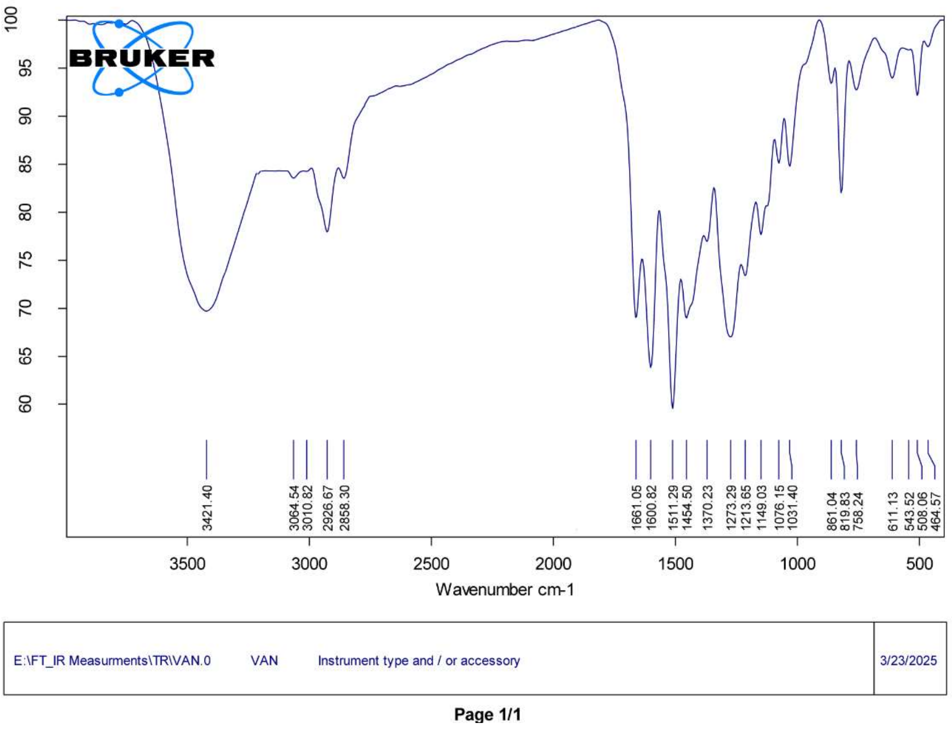

Figure S1. IR of Compound **1**

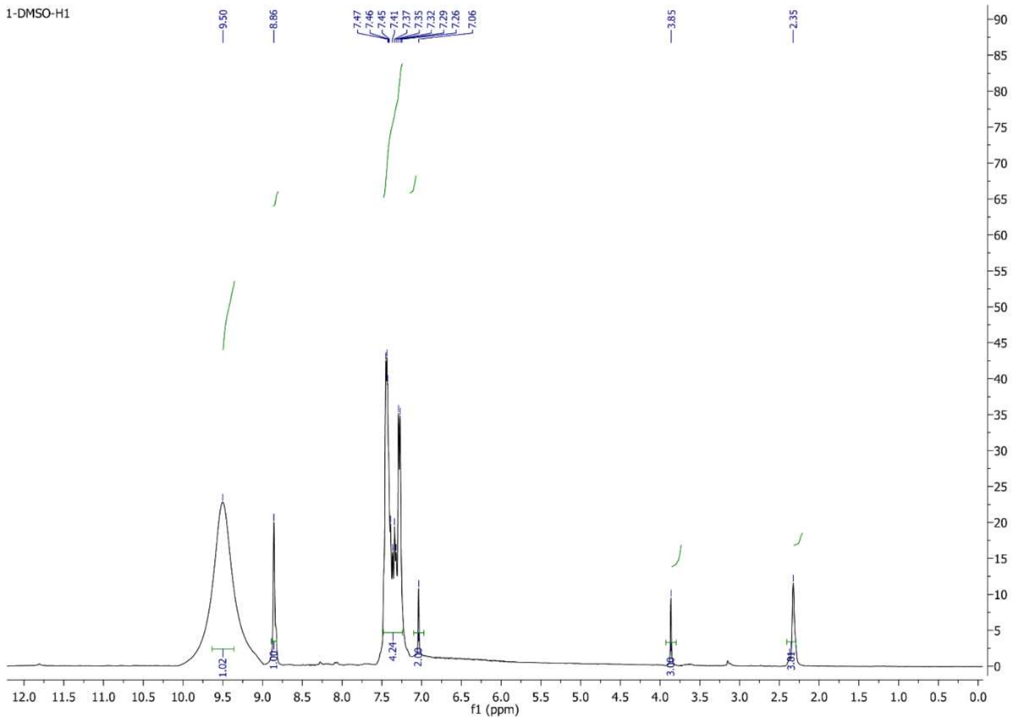

Figure S2. ^{1}H NMR spectrum (400 MHz, DMSO) of compound **1**

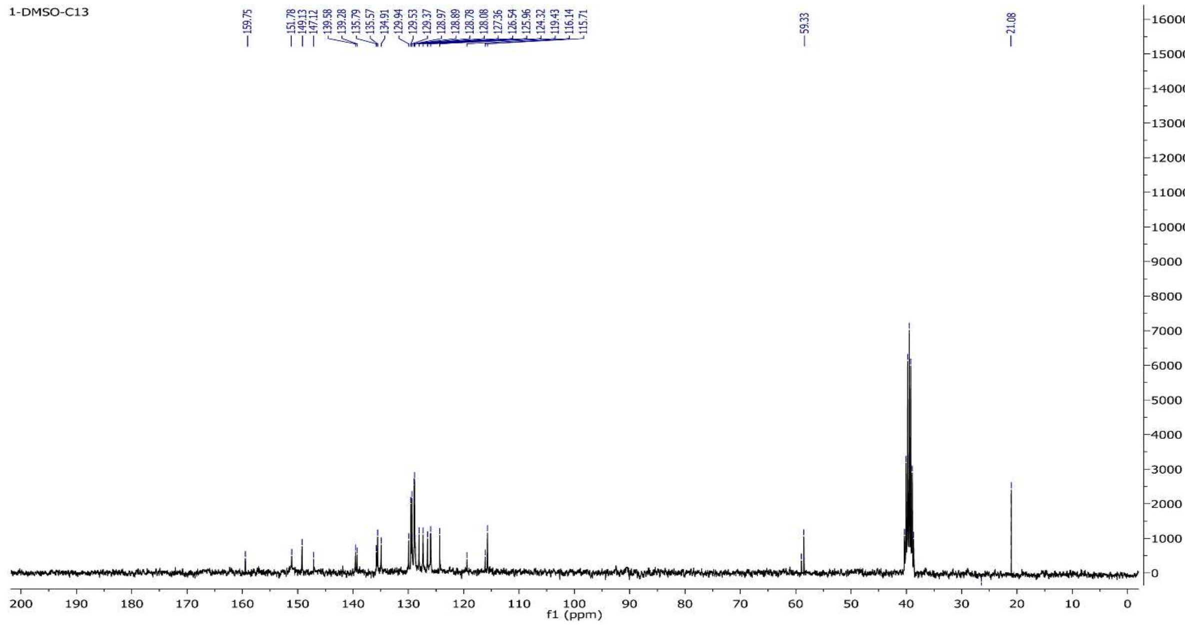

Figure S3. ^{13}C NMR spectrum (100 MHz, DMSO) of compound **1**

Characterization of Compound 2:

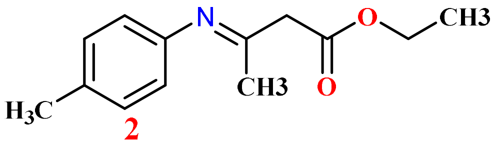

2

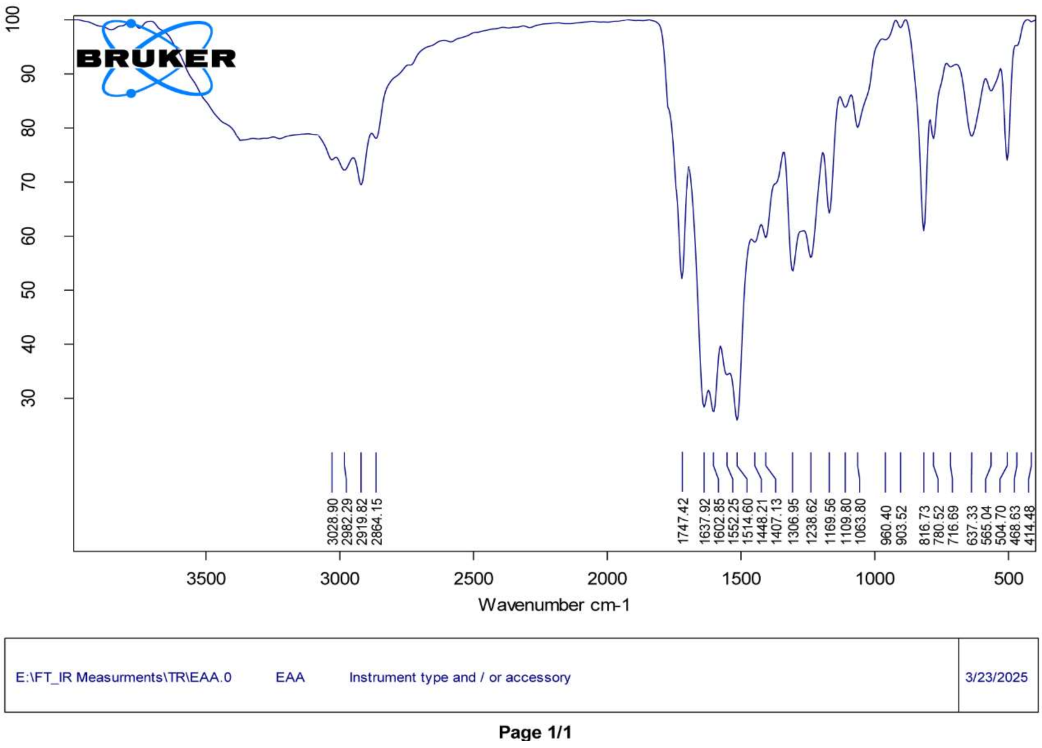

Figure S4. IR of Compound **2**

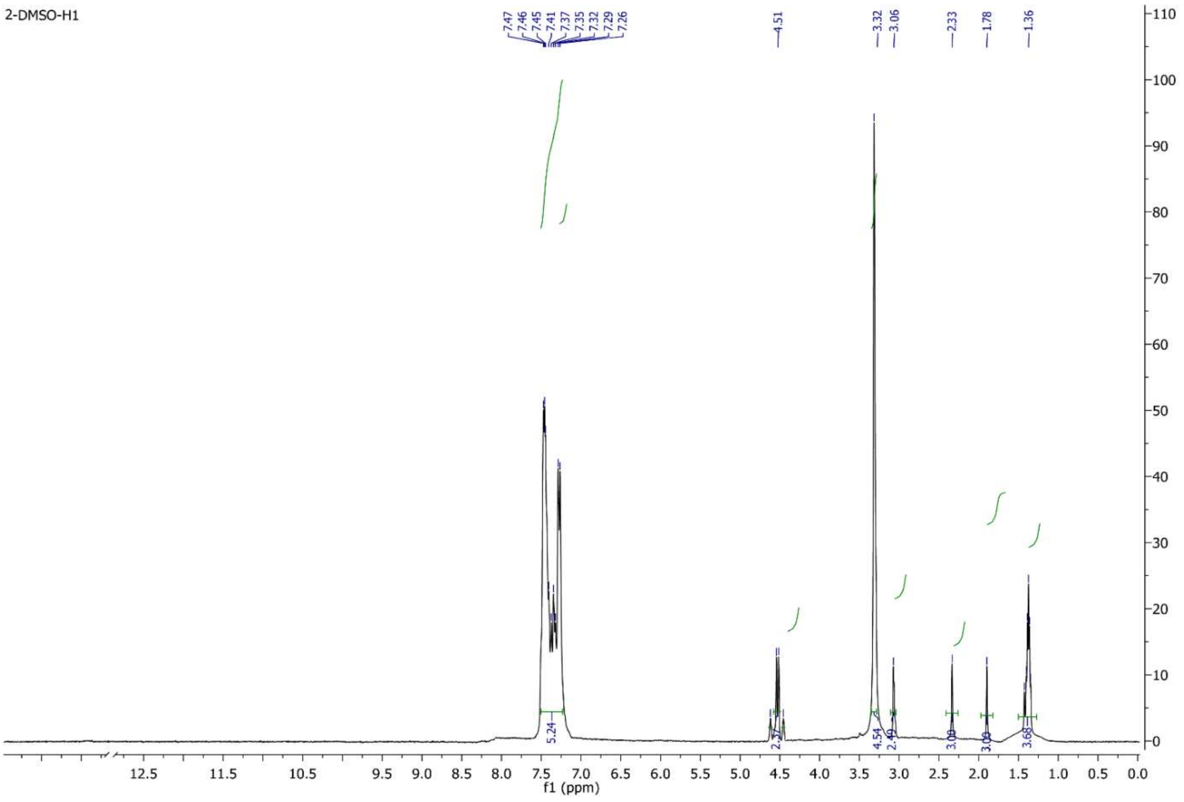

Figure S5. ^{1}H NMR spectrum (400 MHz, DMSO) of compound **2**

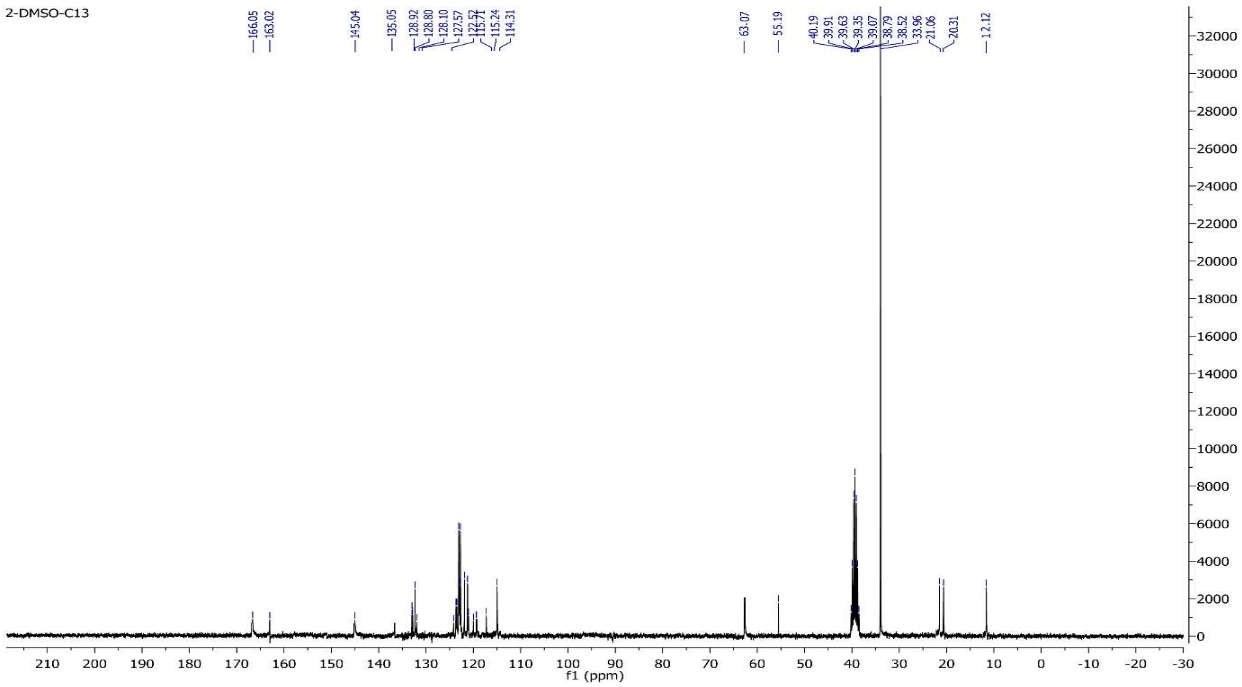

Figure S6. ^{13}C NMR spectrum (100 MHz, DMSO) of compound **2**

Characterization of Compound 3:

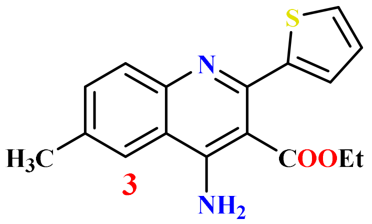

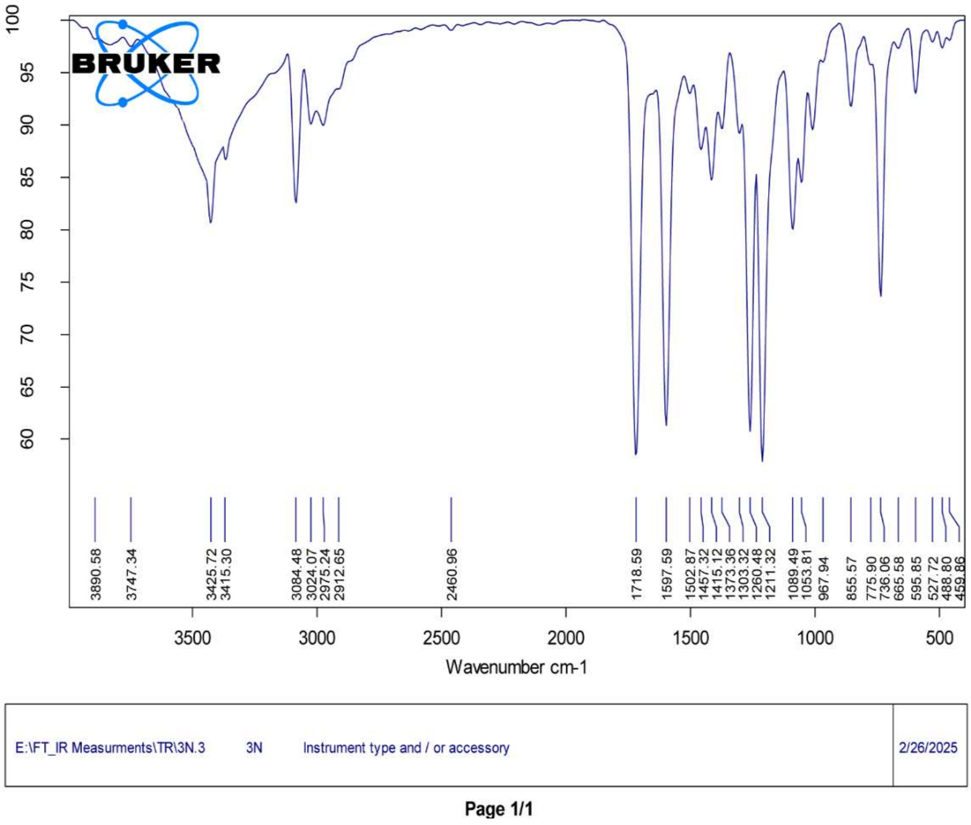

Figure S7. IR of Compound **3**

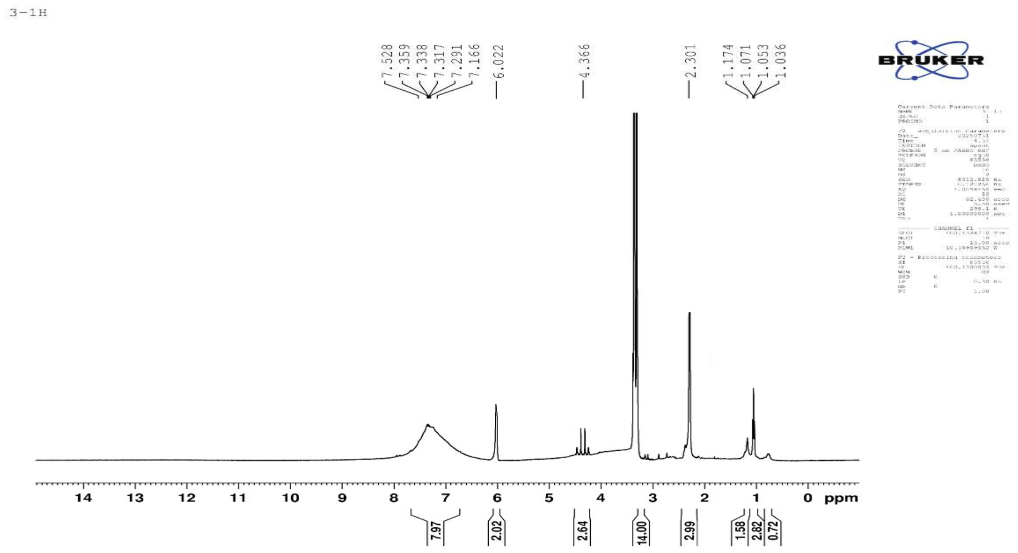

Figure S8. ^{1}H NMR spectrum (400 MHz, DMSO) of compound **3**

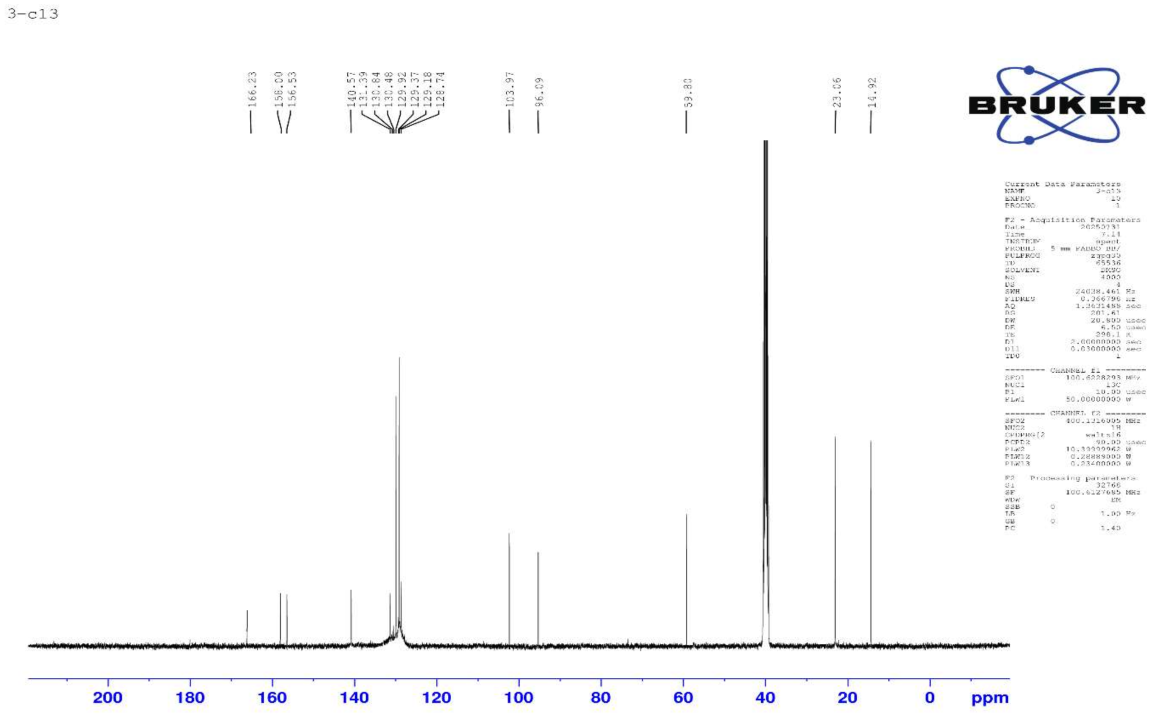

Figure S9. ^{13}C NMR spectrum (100 MHz, DMSO) of compound **3**

Characterization of Compound 4:

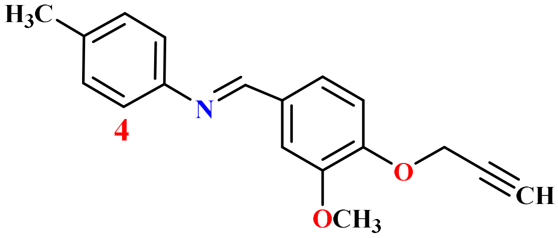

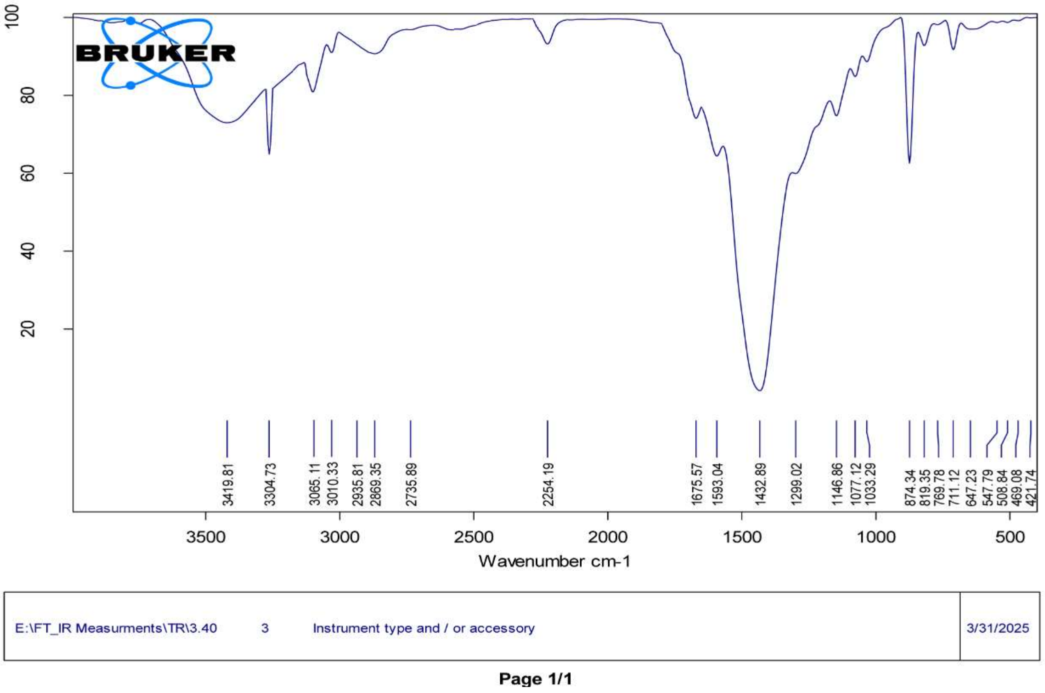

Figure S10. IR of Compound **4**

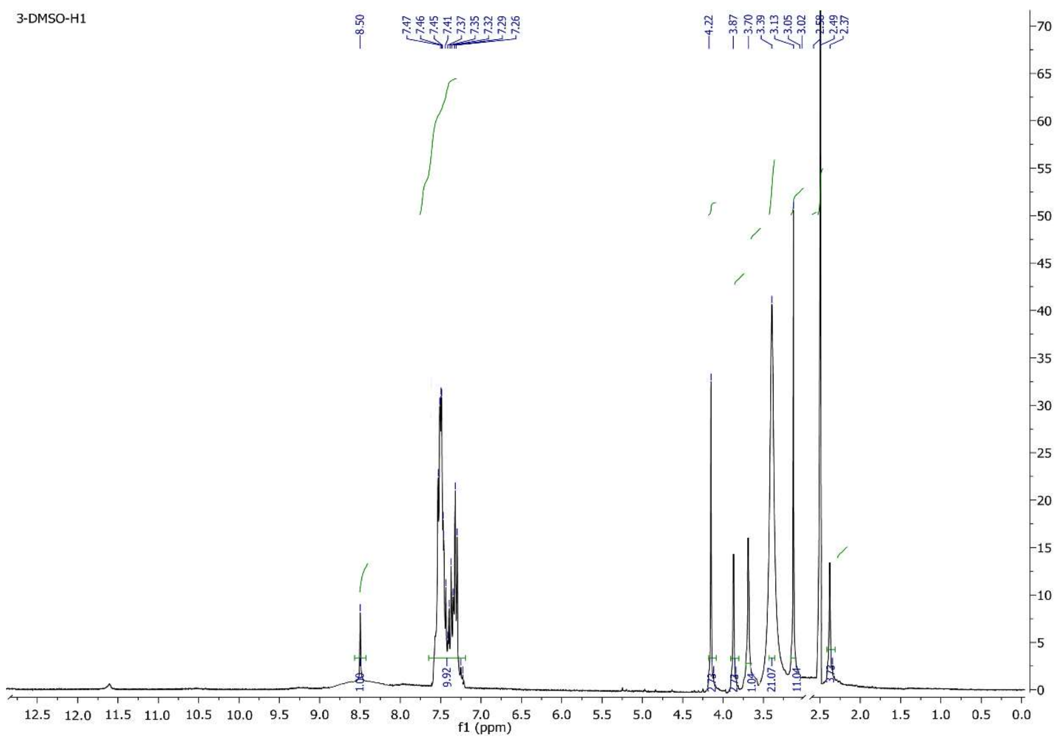

Figure S11. ^{1}H NMR spectrum (400 MHz, DMSO) of compound **4**

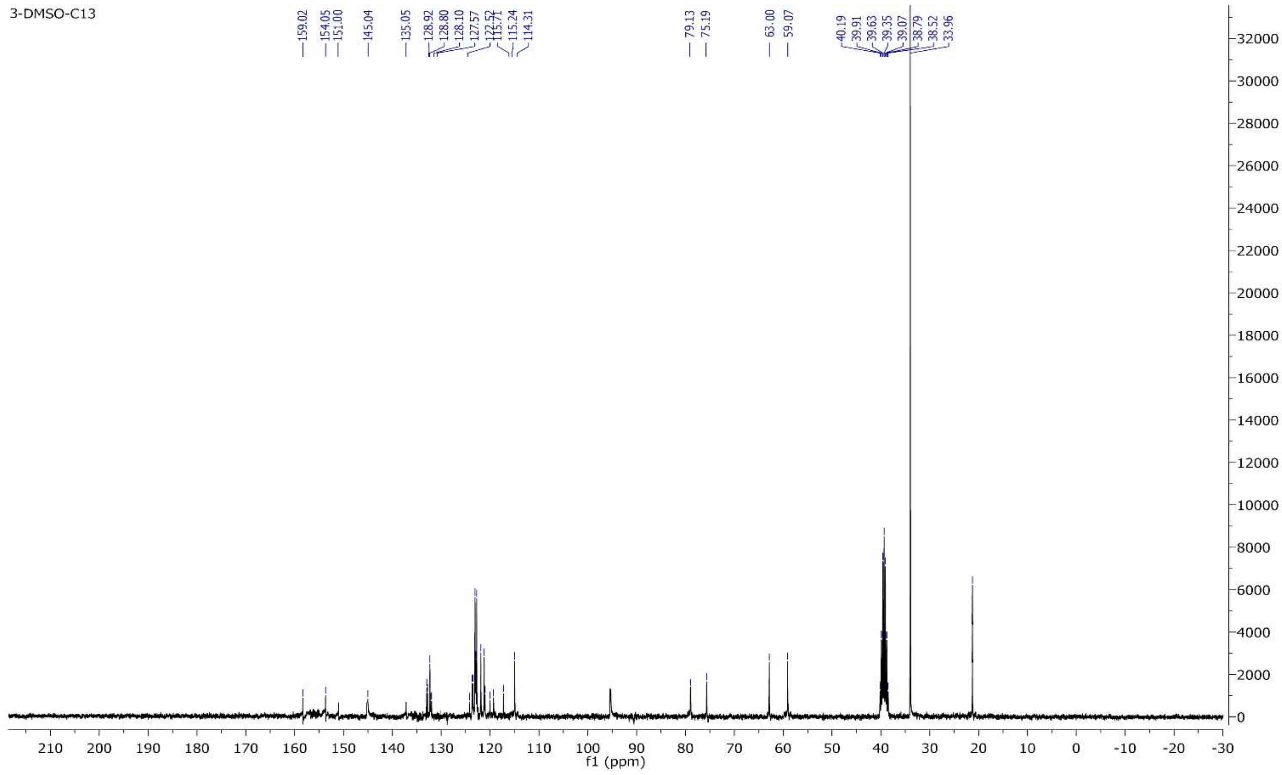

Figure S12. ^{13}C NMR spectrum (100 MHz, DMSO) of compound **4**

Characterization of Compound 5:

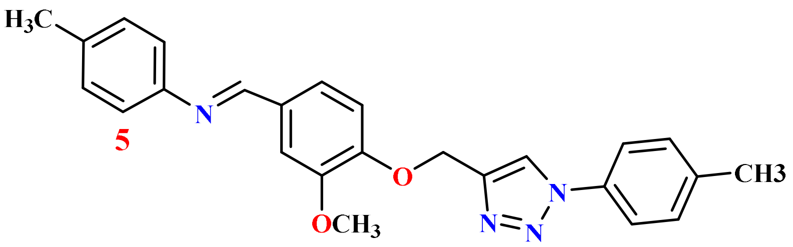

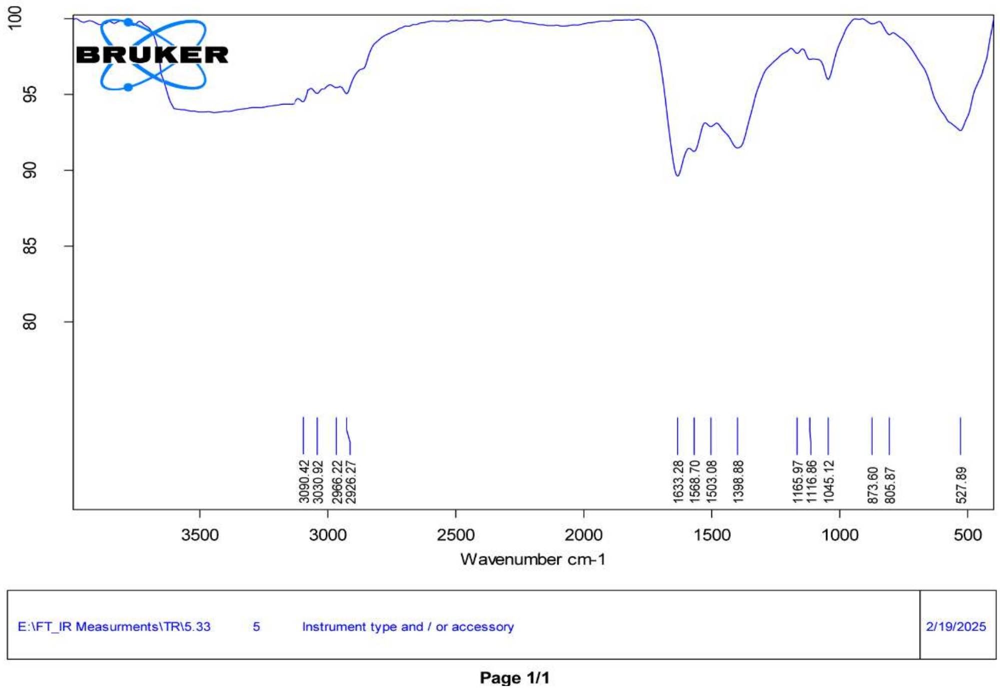

Figure S13. IR of Compound **5**

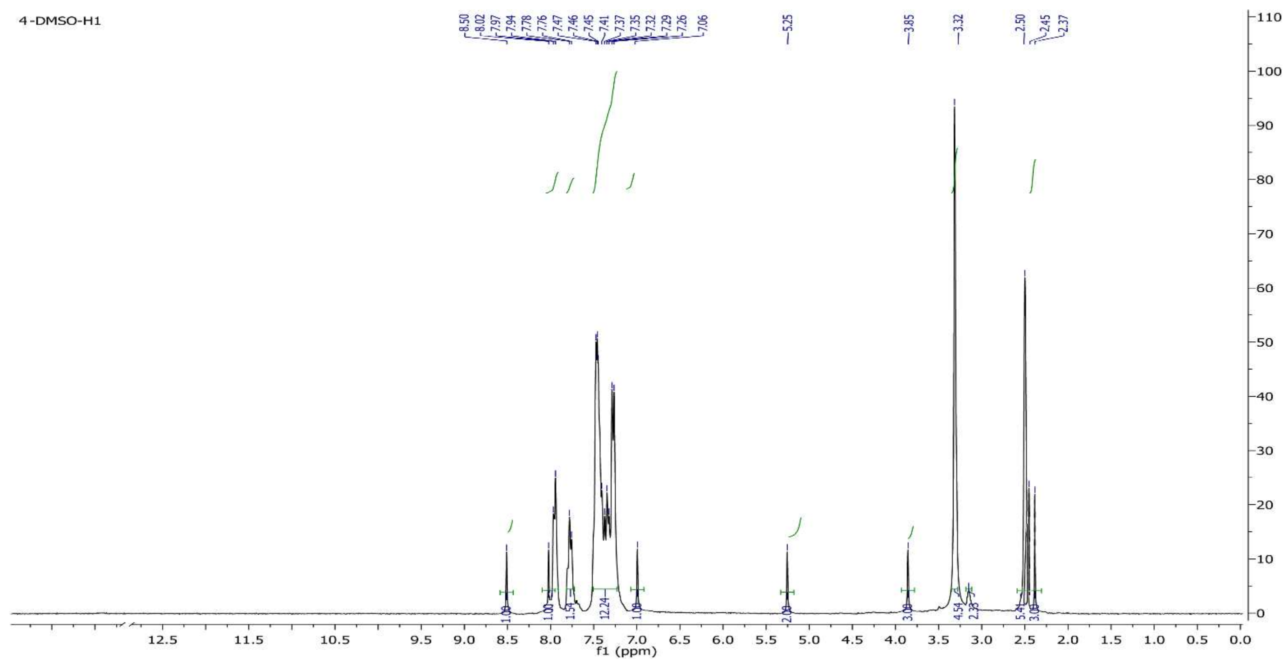

Figure S14. ^{1}H NMR spectrum (400 MHz, DMSO) of compound **5**

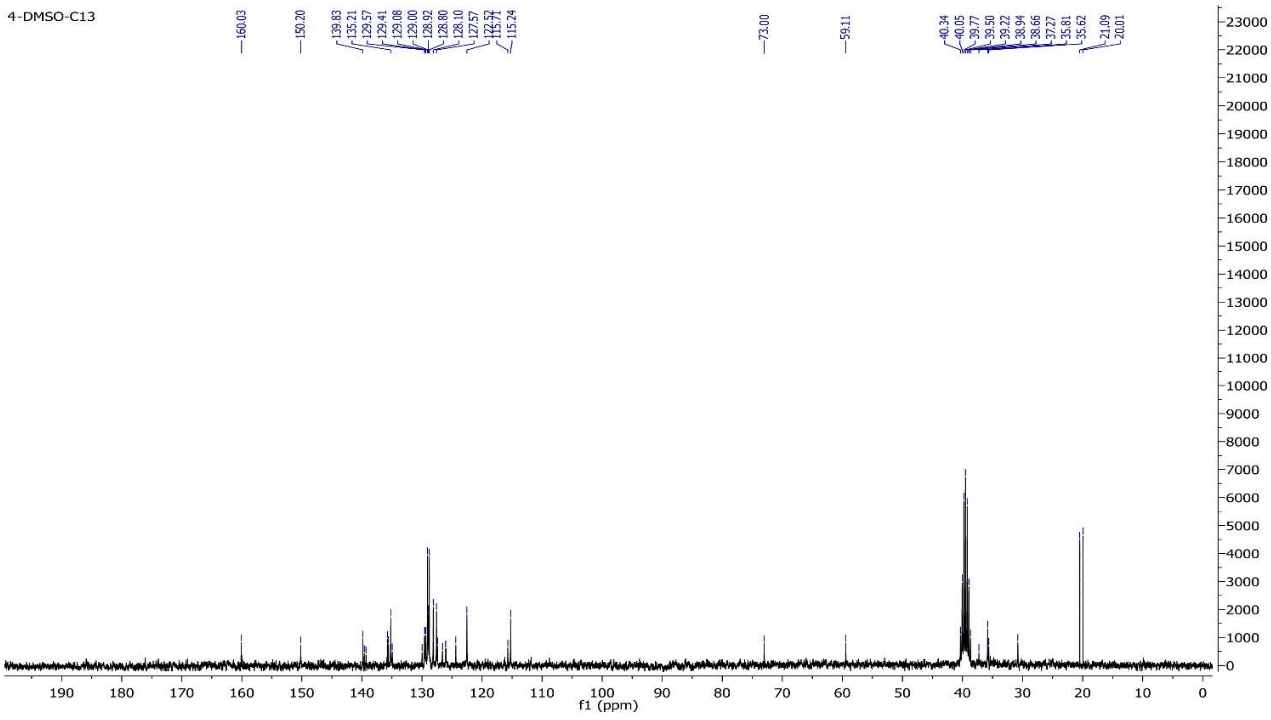

Figure S15. ^{13}C NMR spectrum (100 MHz, DMSO) of compound **5**

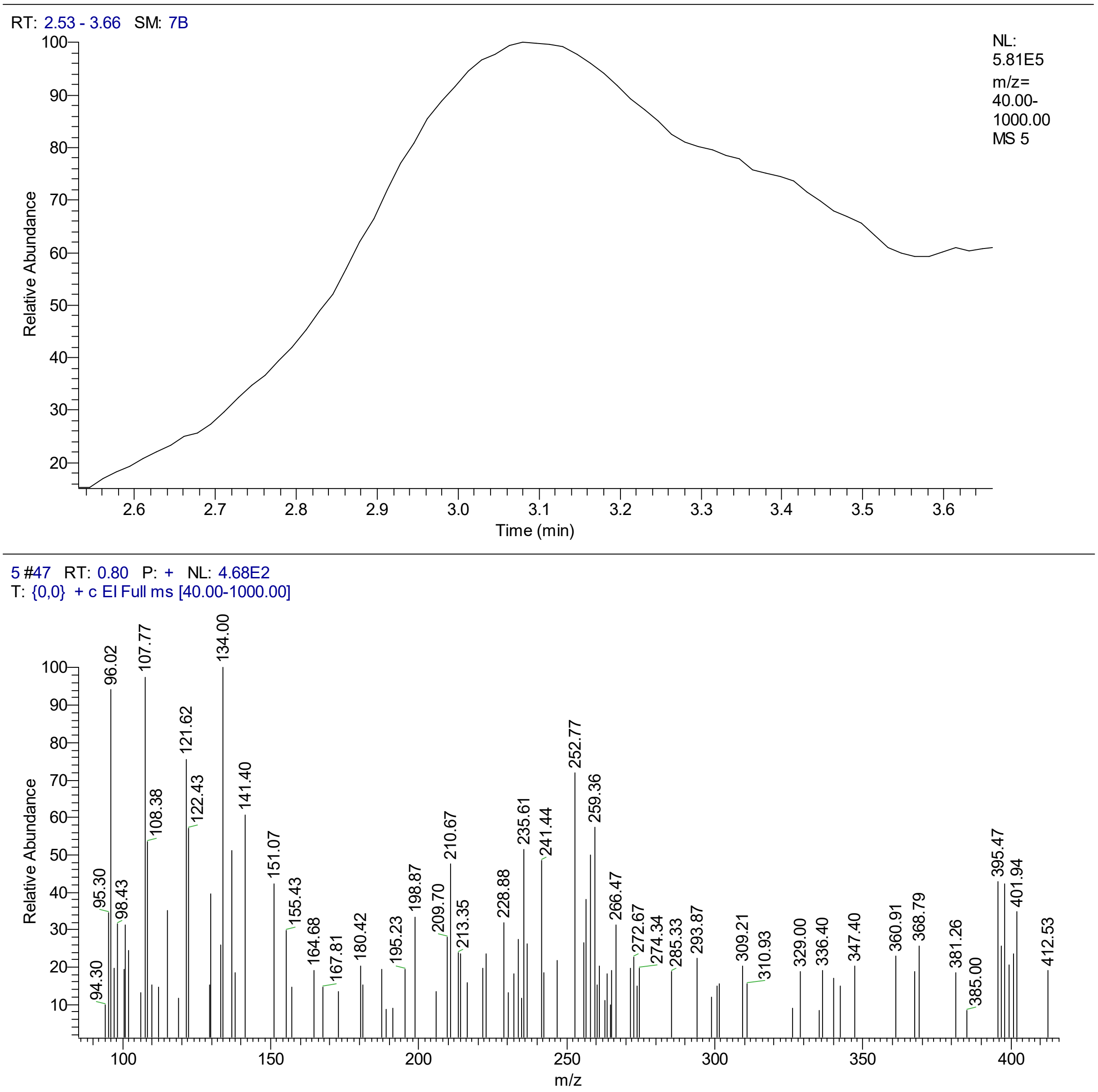

Figure S16. Mass spectrum of compound **5**

Characterization of Compound 6:

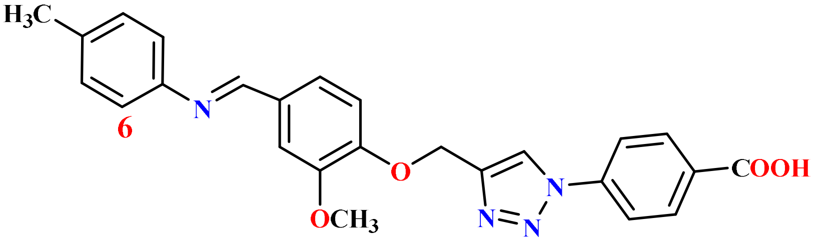

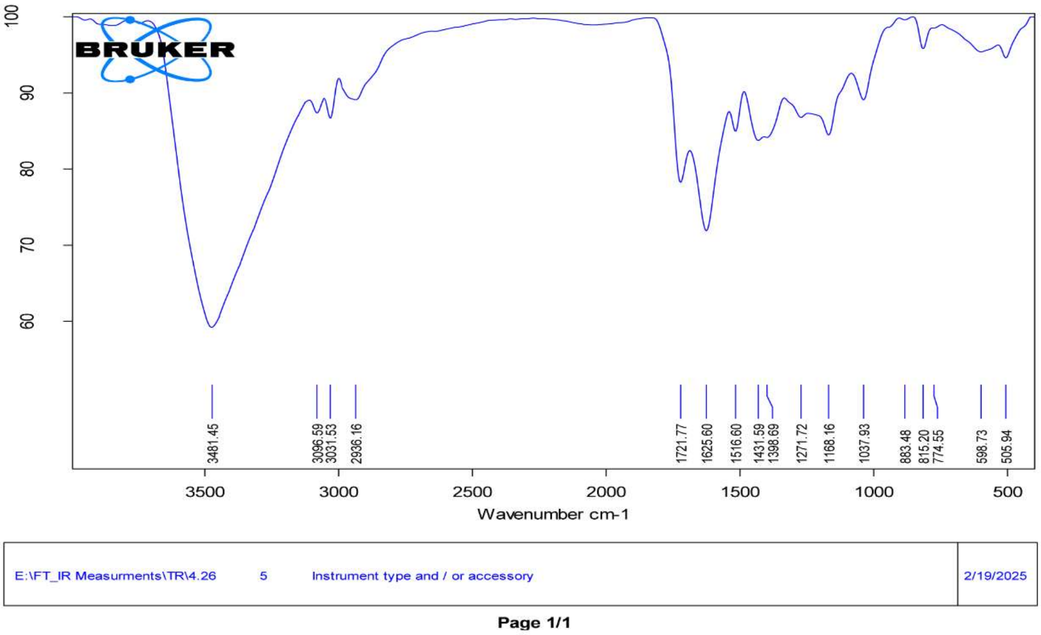

Figure S17. IR of Compound **6**

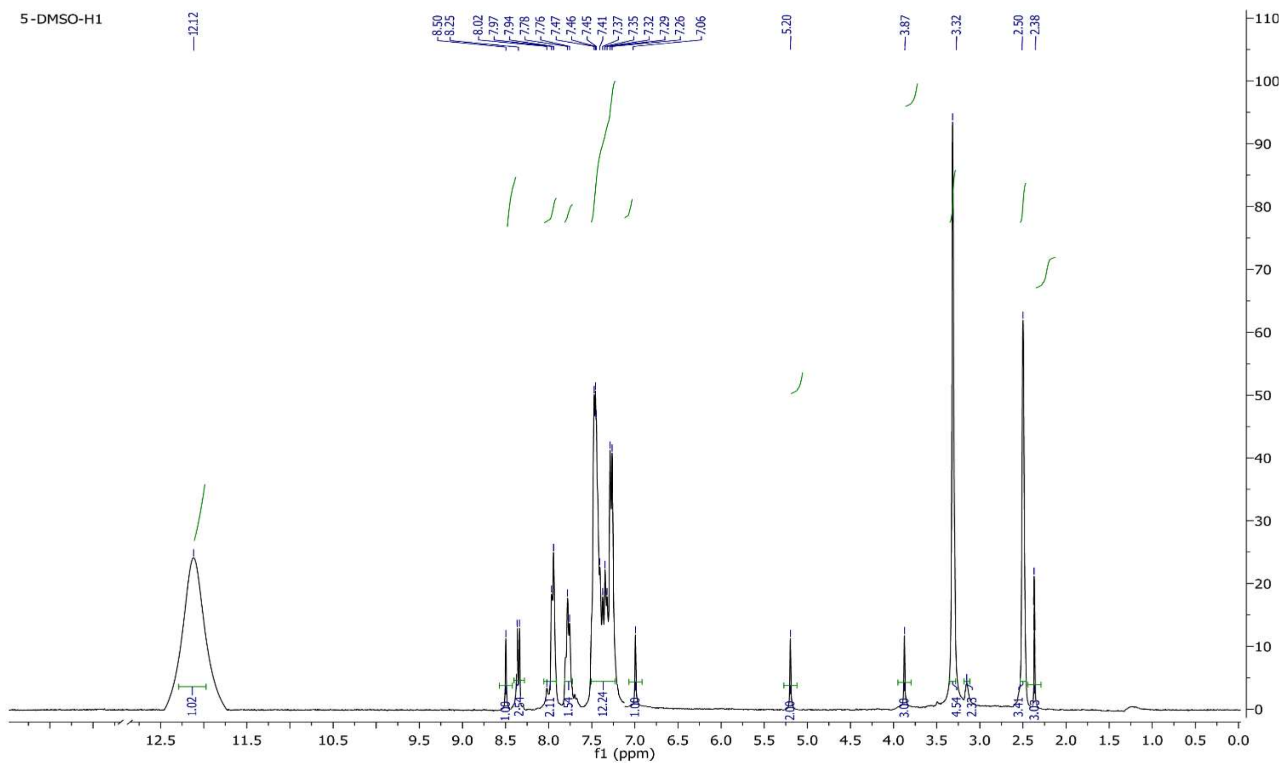

Figure S18. ^{1}H NMR spectrum (400 MHz, DMSO) of compound **6**

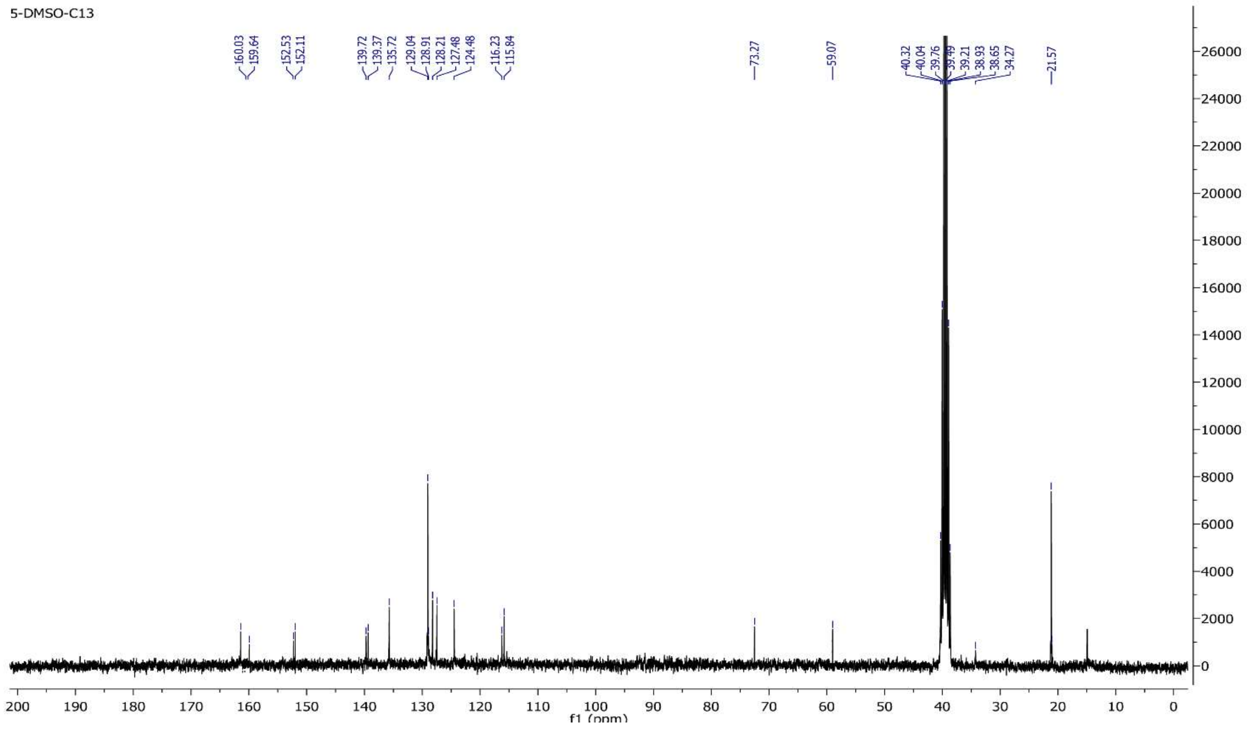

Figure S19. ^{13}C NMR spectrum (100 MHz, DMSO) of compound **6**

Characterization of Compound 7:

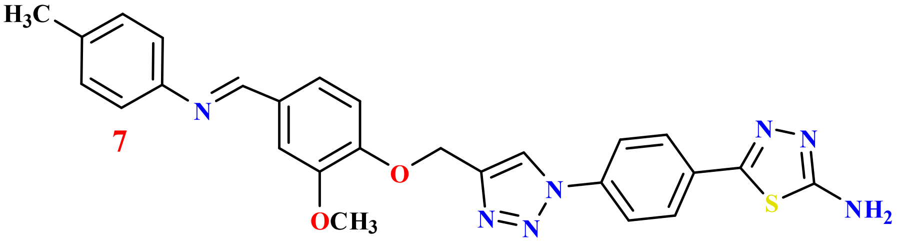

7

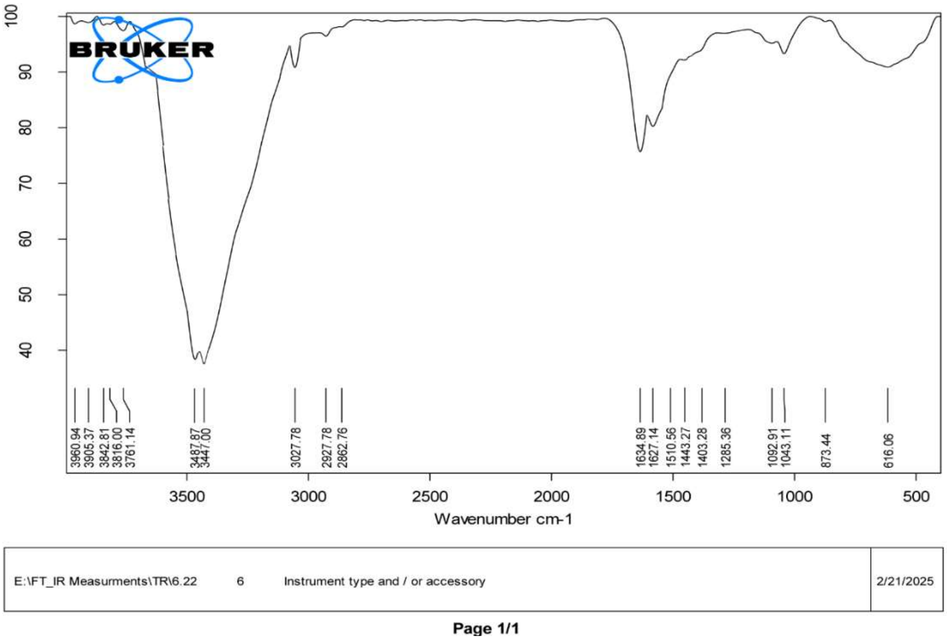

Figure S20. IR of Compound **7**

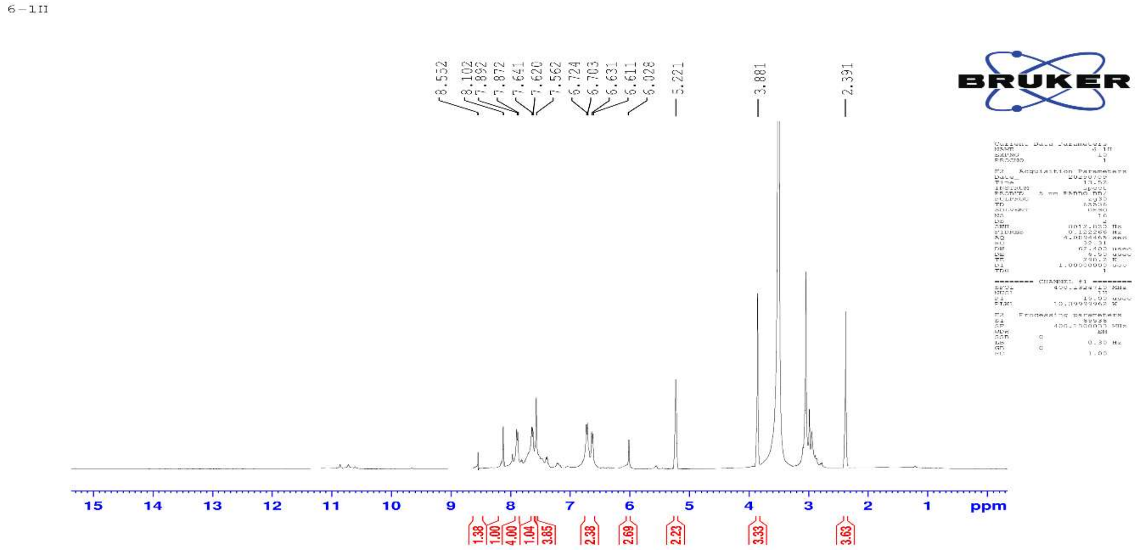

Figure S21. ^{1}H NMR spectrum (400 MHz, DMSO) of compound **7**

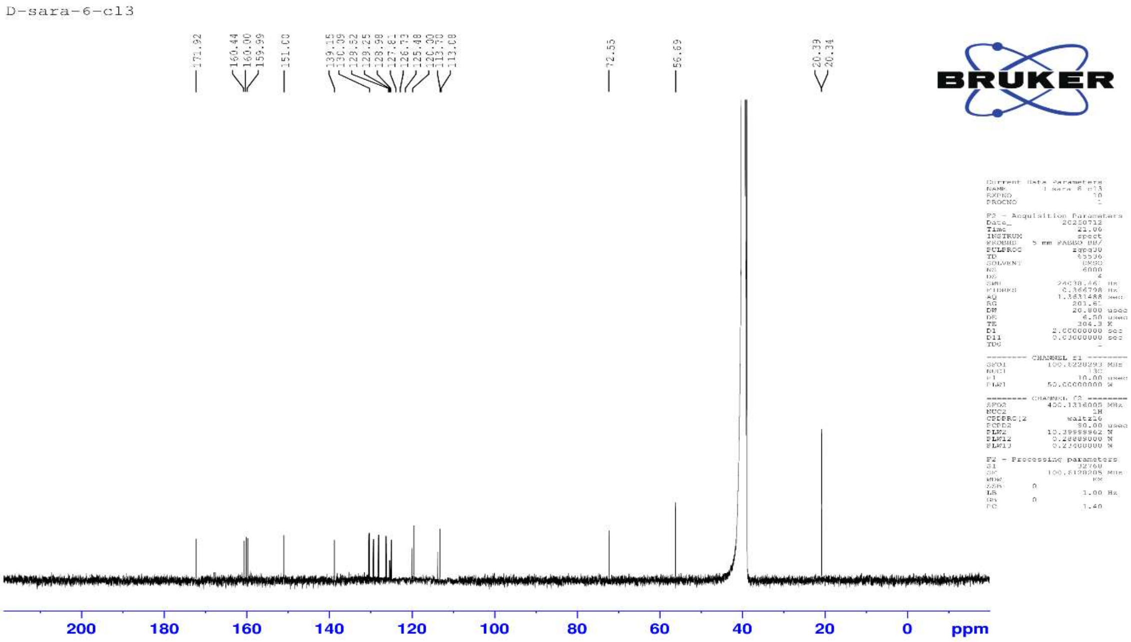

Figure S22. ^{13}C NMR spectrum (100 MHz, DMSO) of compound **7**

Characterization of Compound 8:

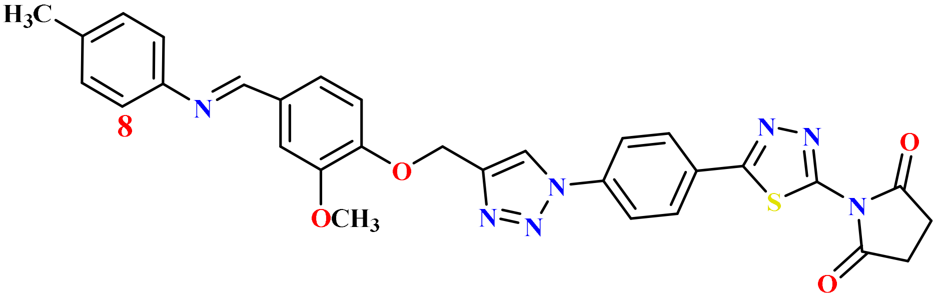

8

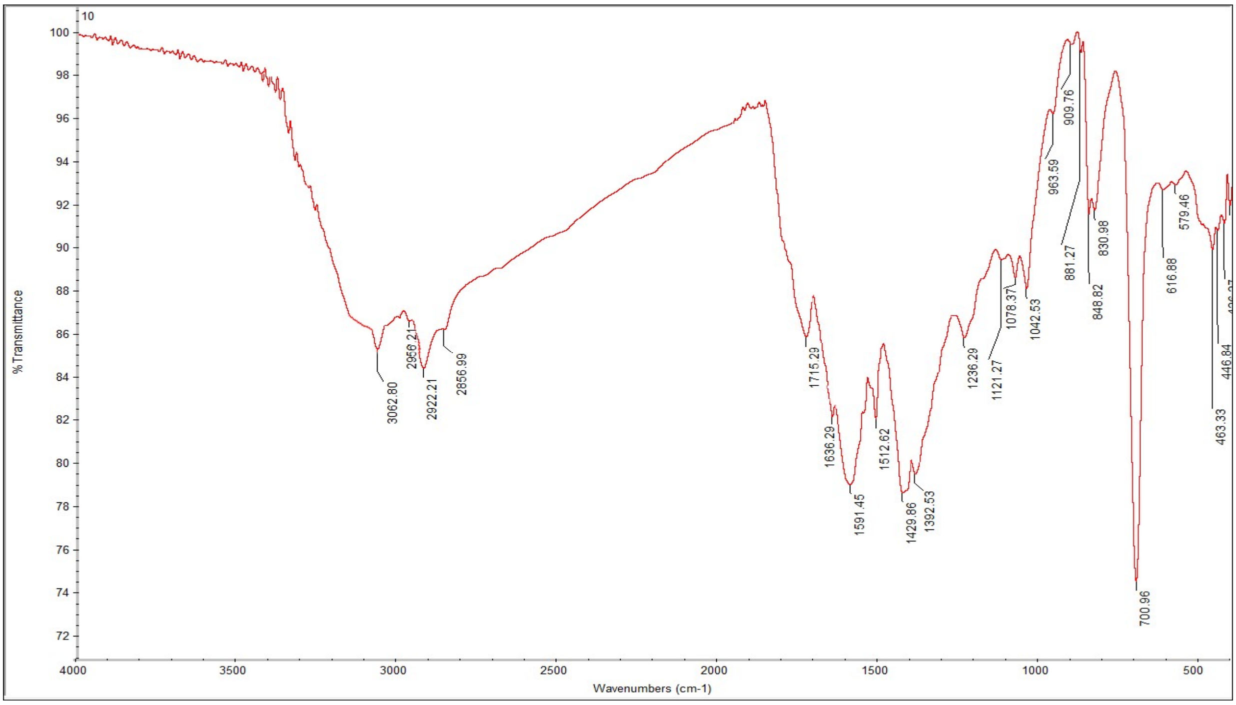

Figure S23. IR of Compound **8**

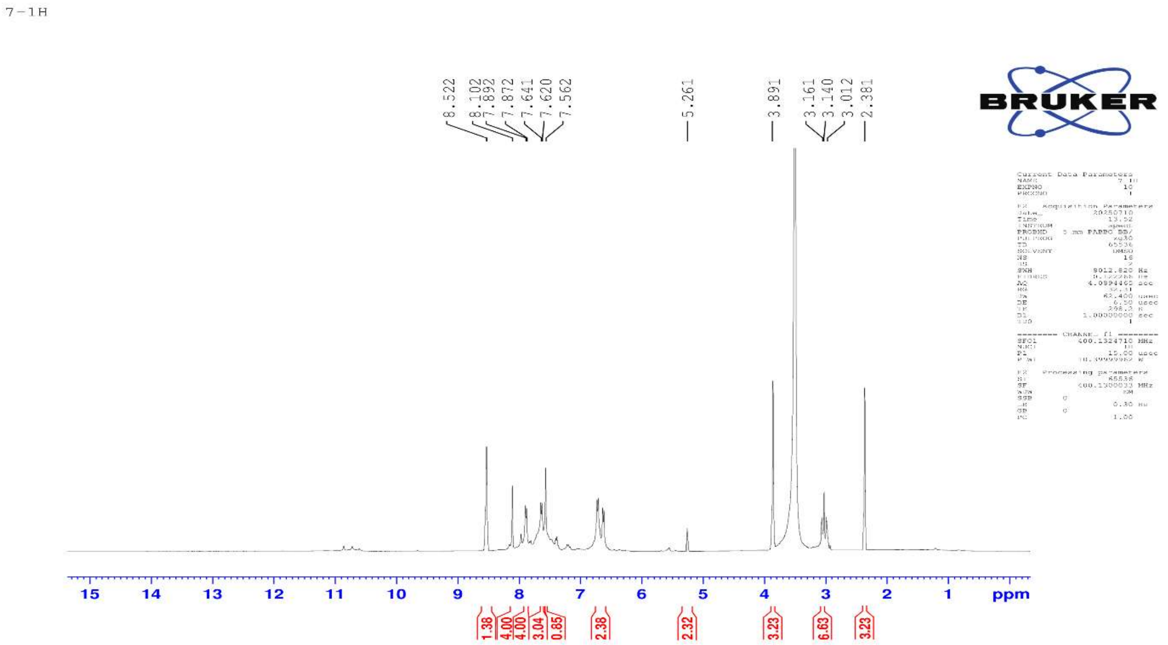

Figure S24. ^{1}H NMR spectrum (400 MHz, DMSO) of compound **8**

7-C13

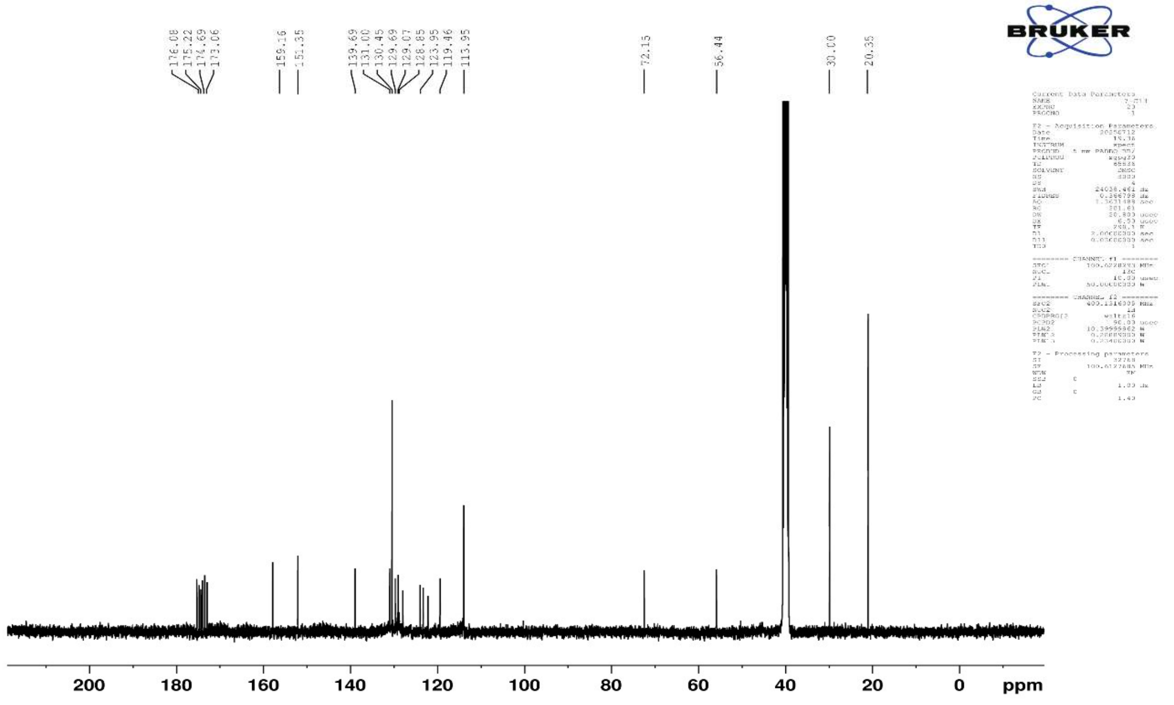

Figure S25. ^{13}C NMR spectrum (100 MHz, DMSO) of compound **8**

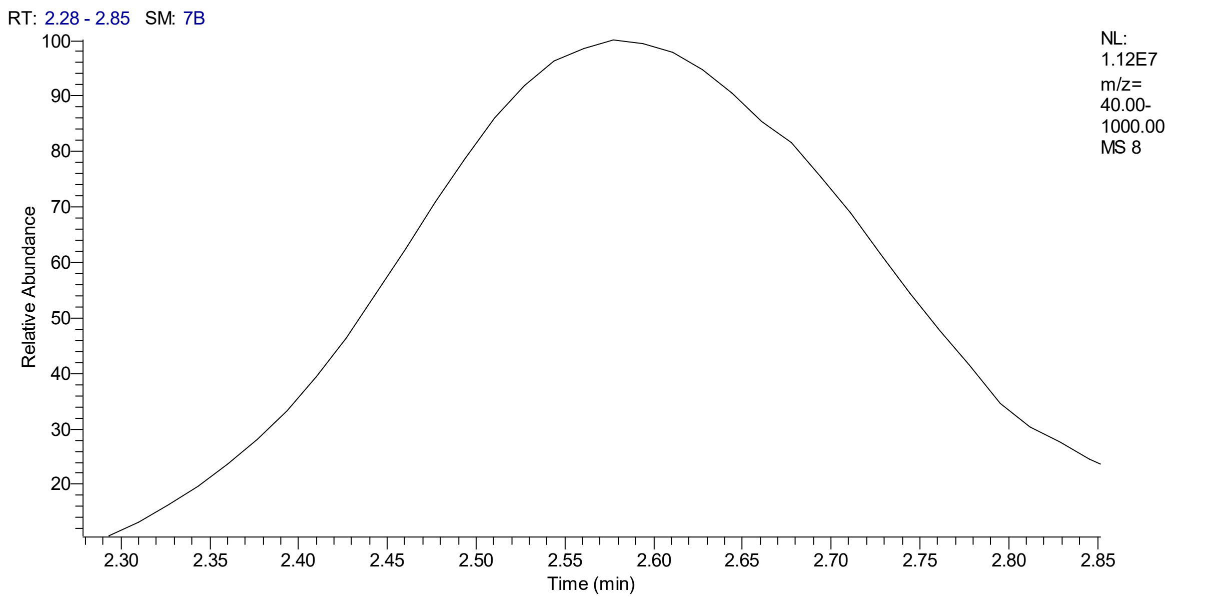

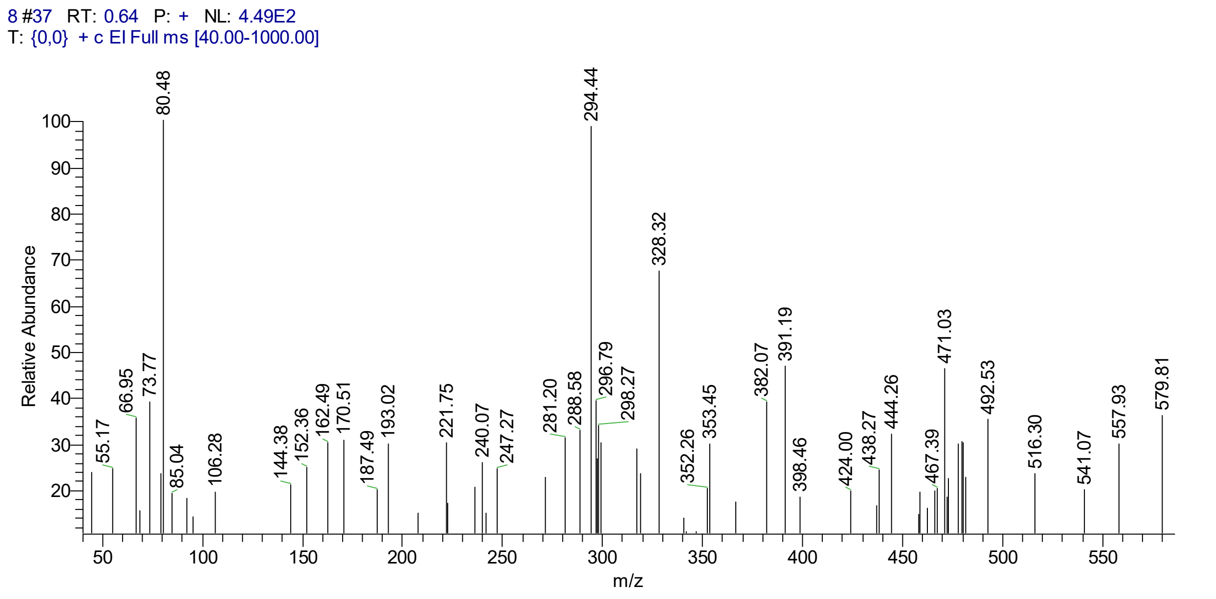

Figure S26. Mass spectrum of compound **8**

Characterization of Compound 9:

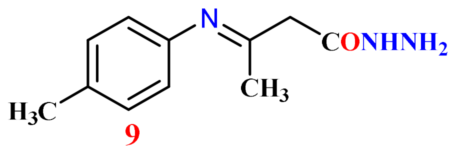

9

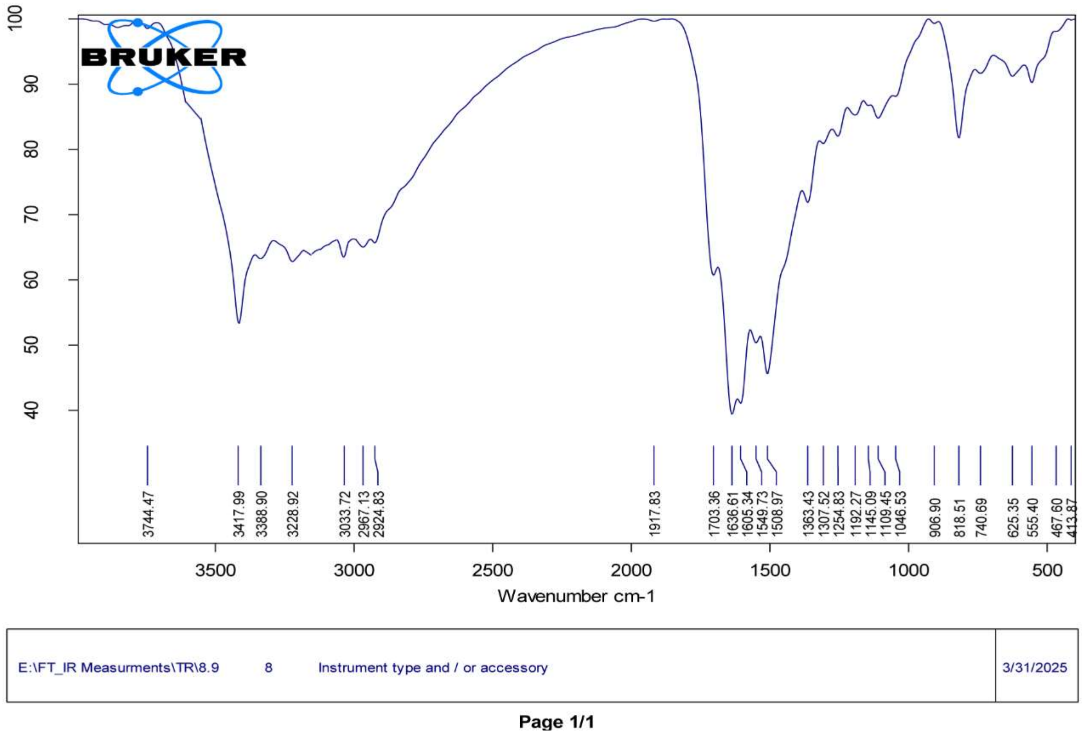

Figure S27. IR of Compound **9**

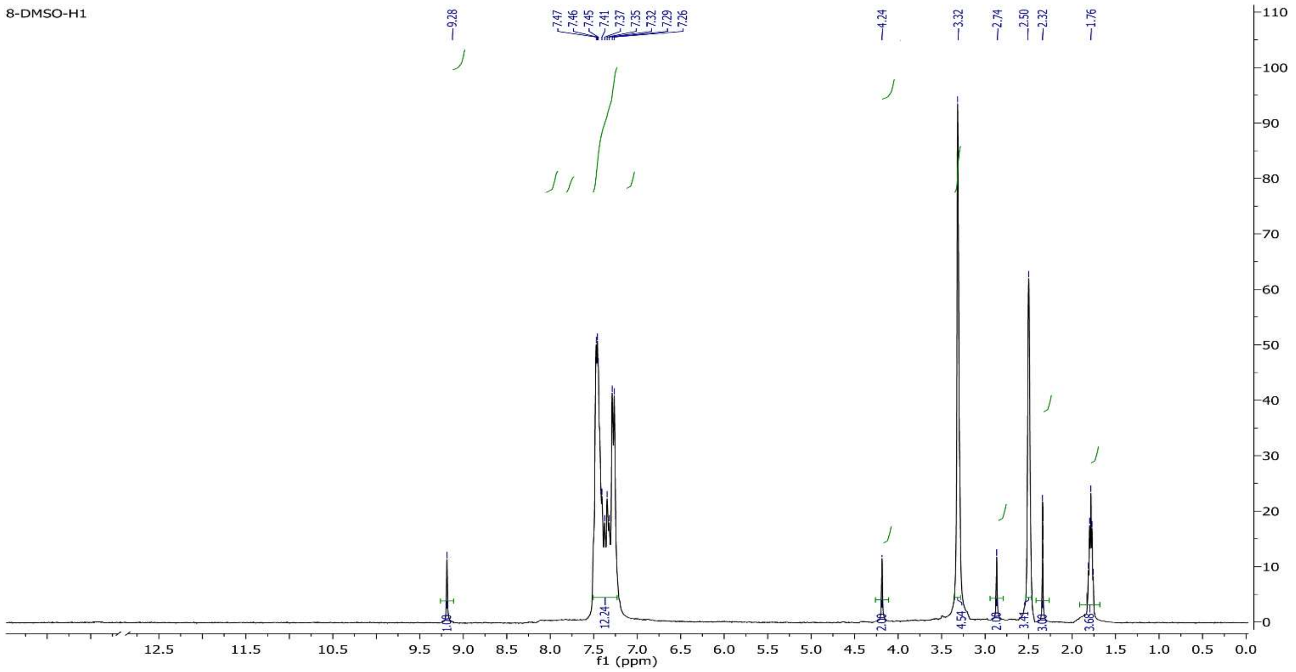

Figure S28. ^{1}H NMR spectrum (400 MHz, DMSO) of compound **9**

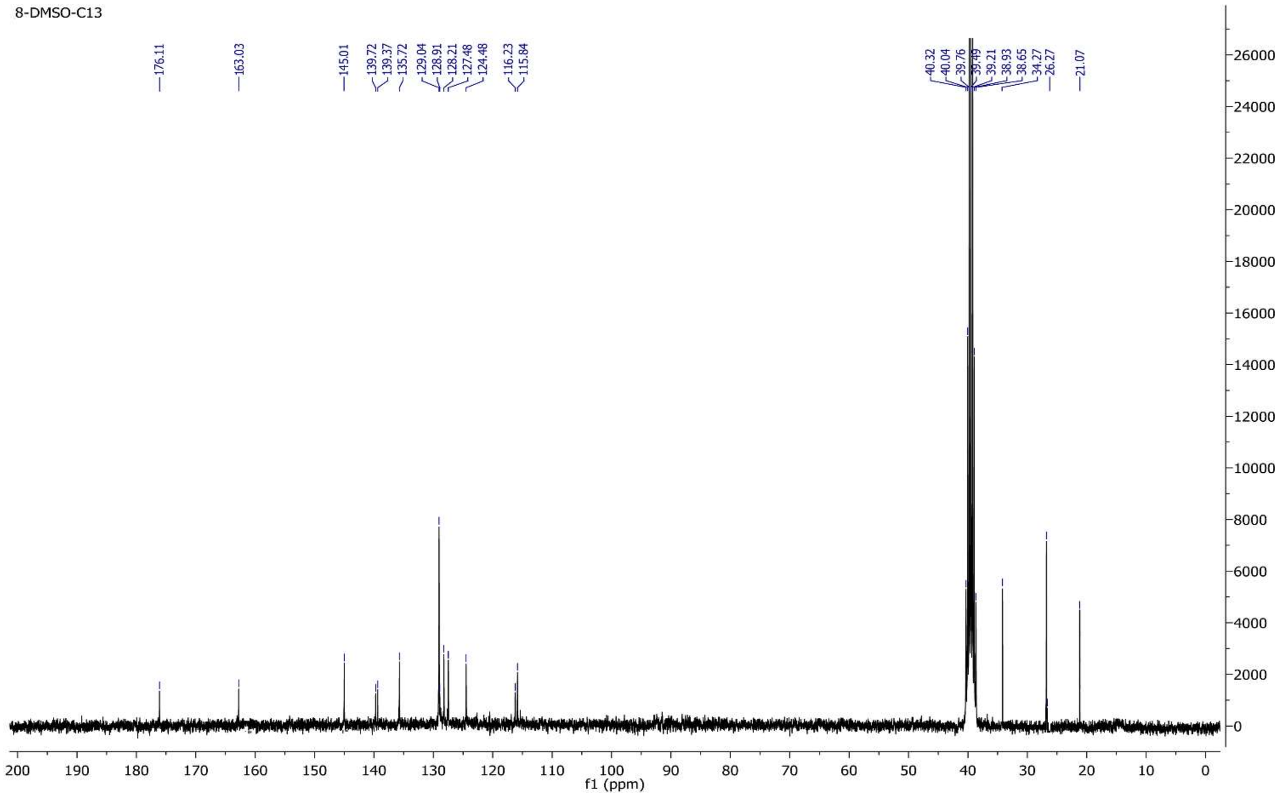

Figure S29. ^{13}C NMR spectrum (100 MHz, DMSO) of compound **9**

Characterization of Compound 10:

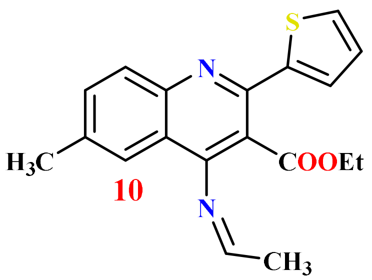

10

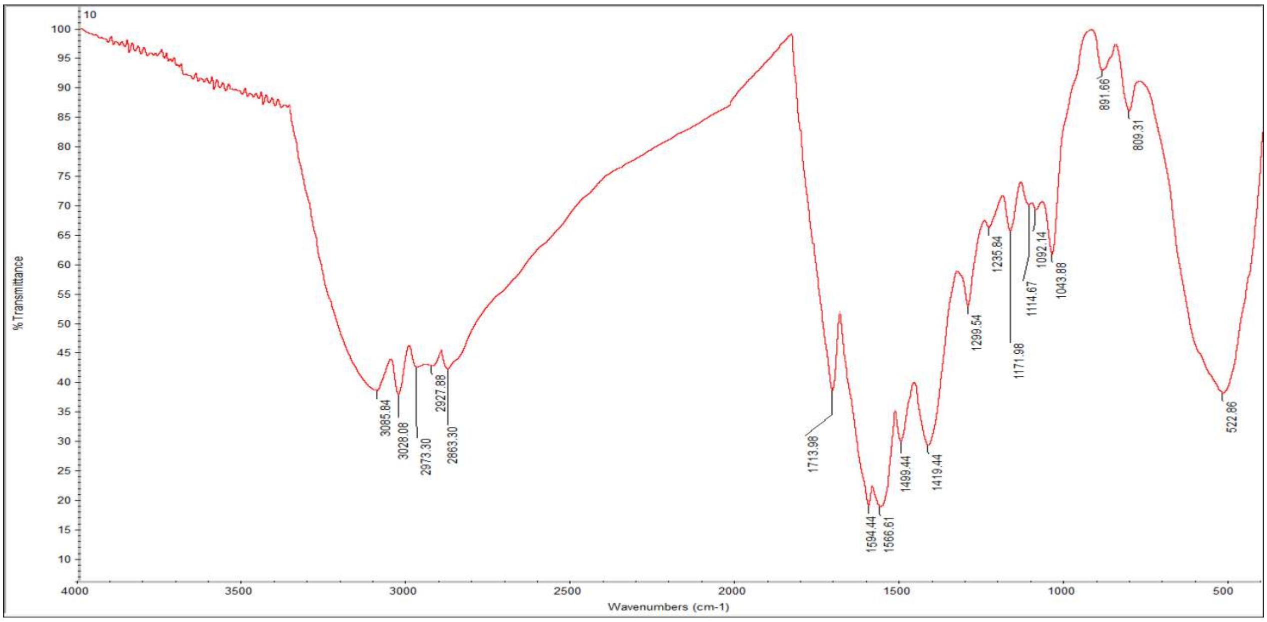

Figure S30. IR of Compound **10**

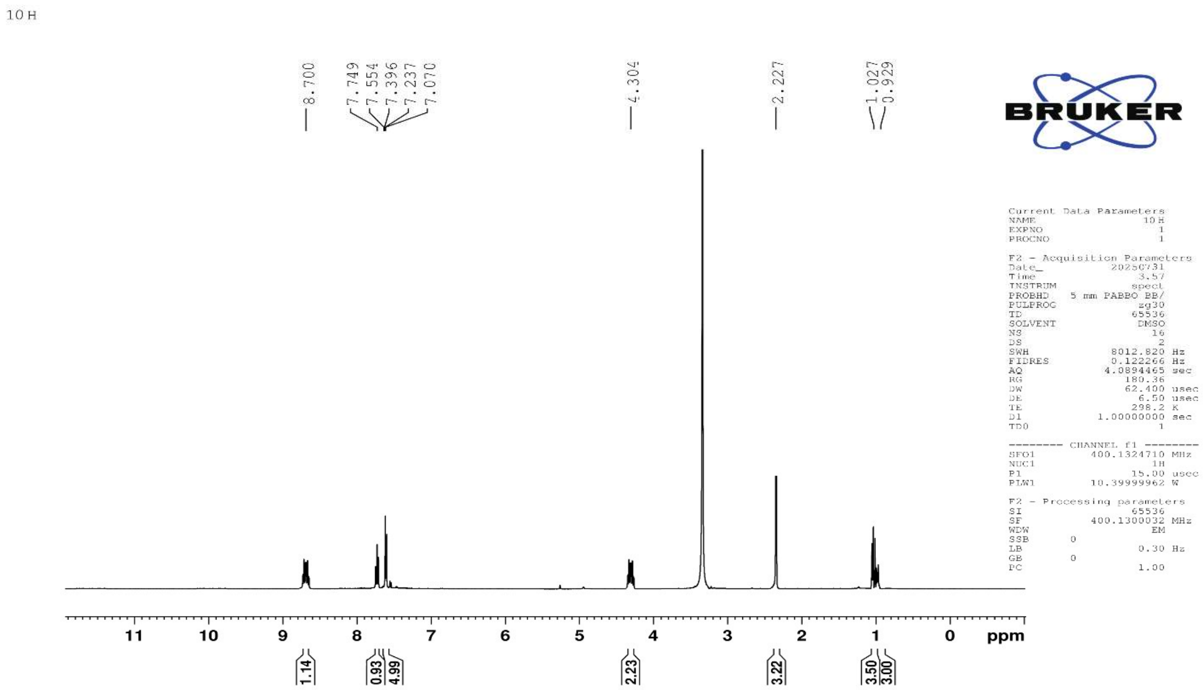

Figure S31. ^{1}H NMR spectrum (400 MHz, DMSO) of compound **10**

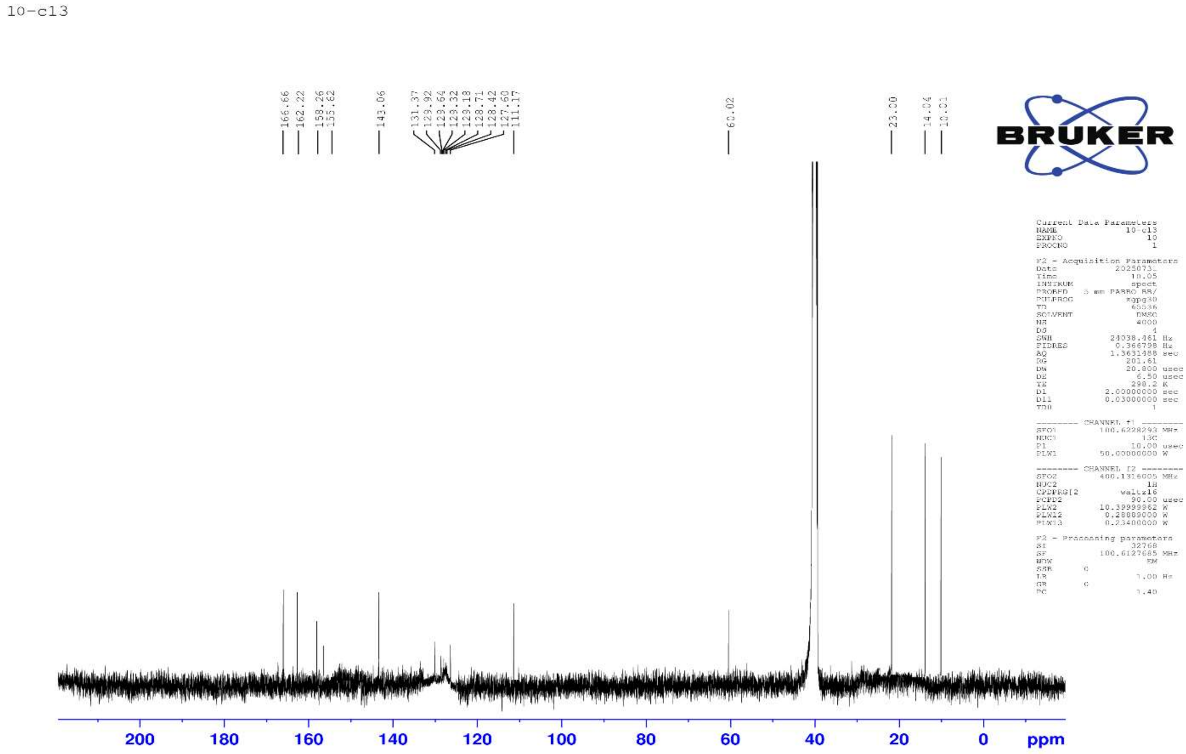

Figure S32. ^{13}C NMR spectrum (100 MHz, DMSO) of compound **10**

Characterization of Compound 11:

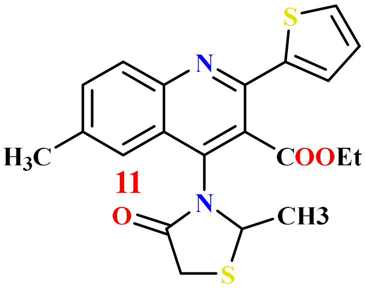

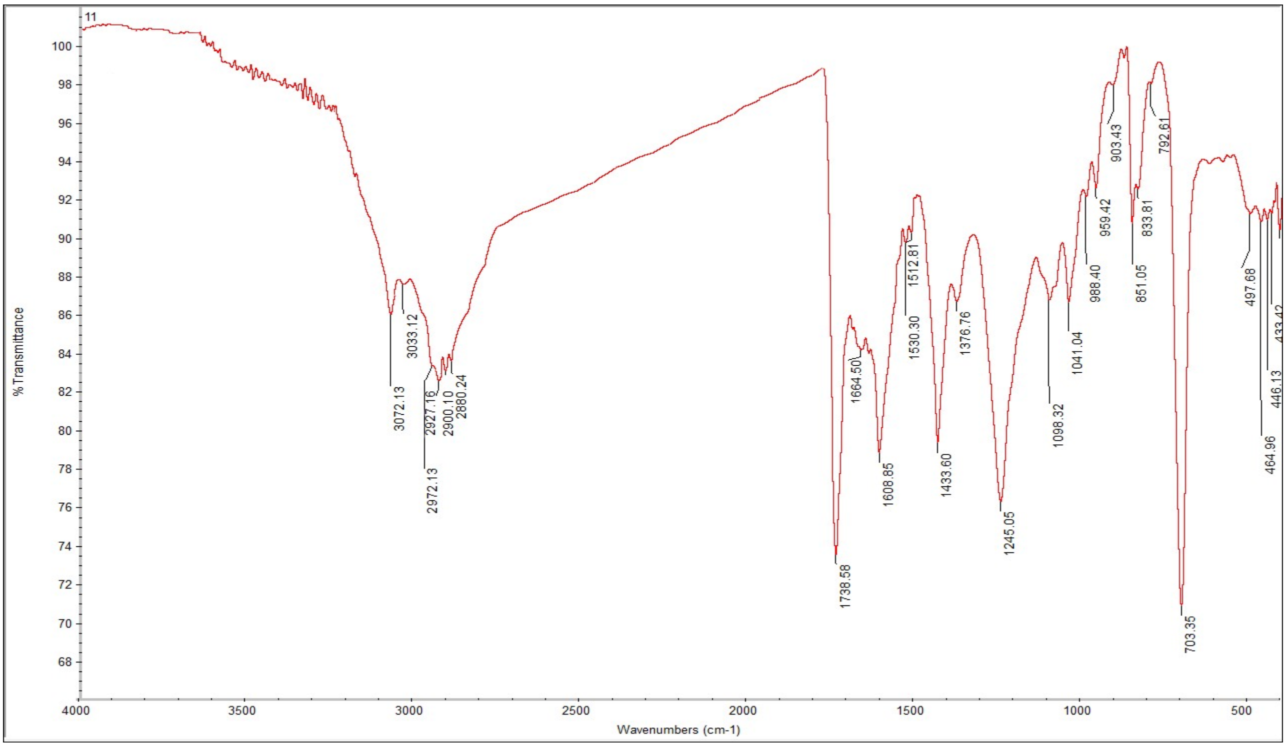

Figure S33. IR of Compound **11**

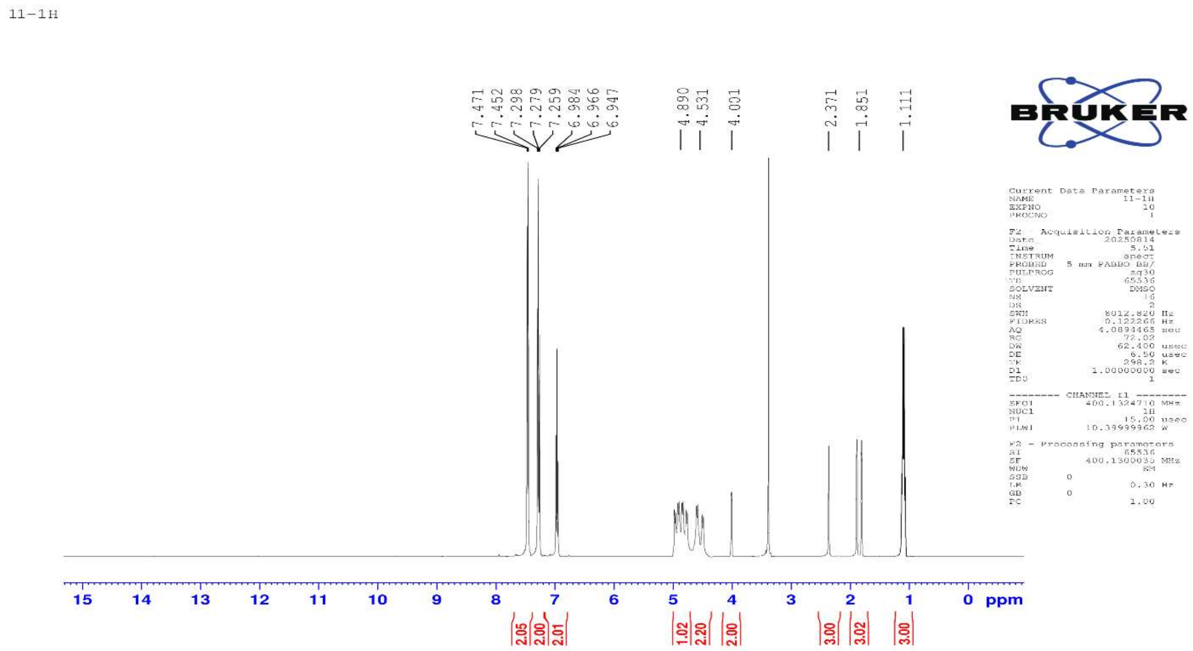

Figure S34. ^{1}H NMR spectrum (400 MHz, DMSO) of compound **11**

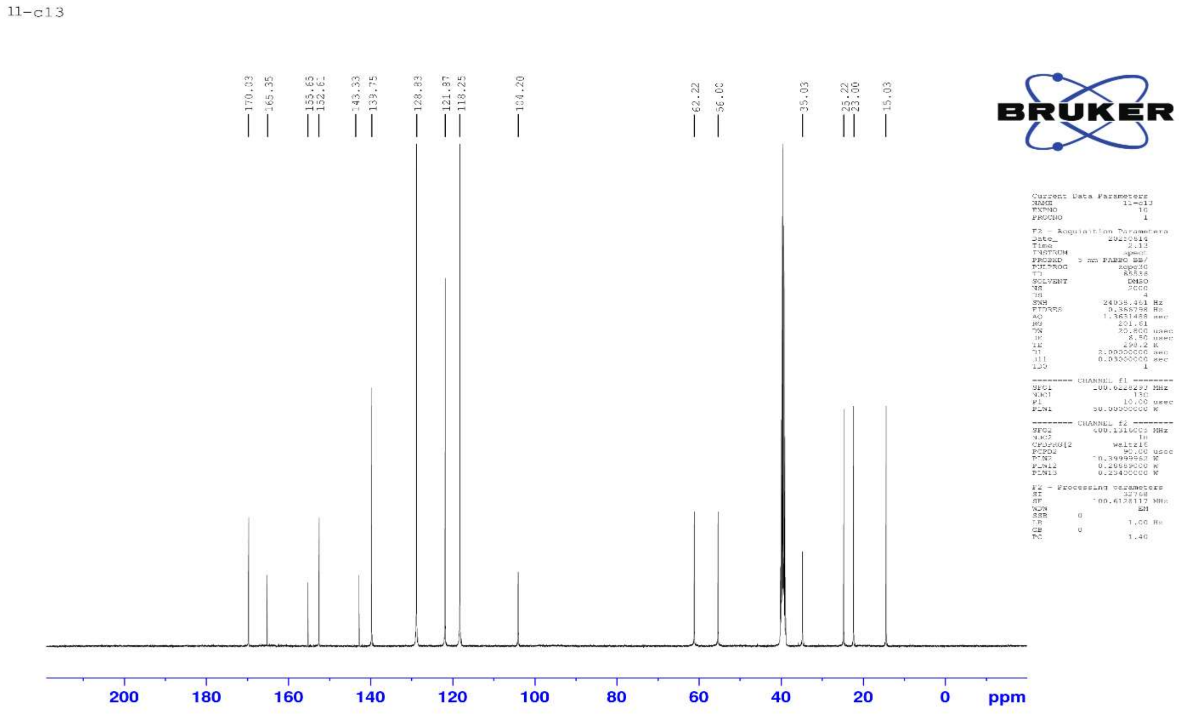

Figure S35. ^{13}C NMR spectrum (100 MHz, DMSO) of compound **11**

Characterization of Compound 12:

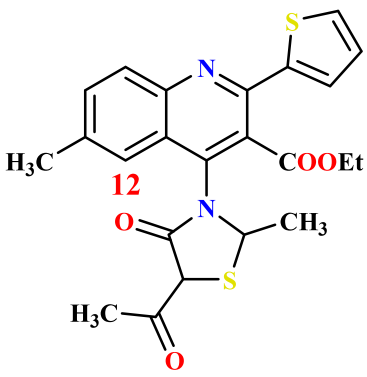

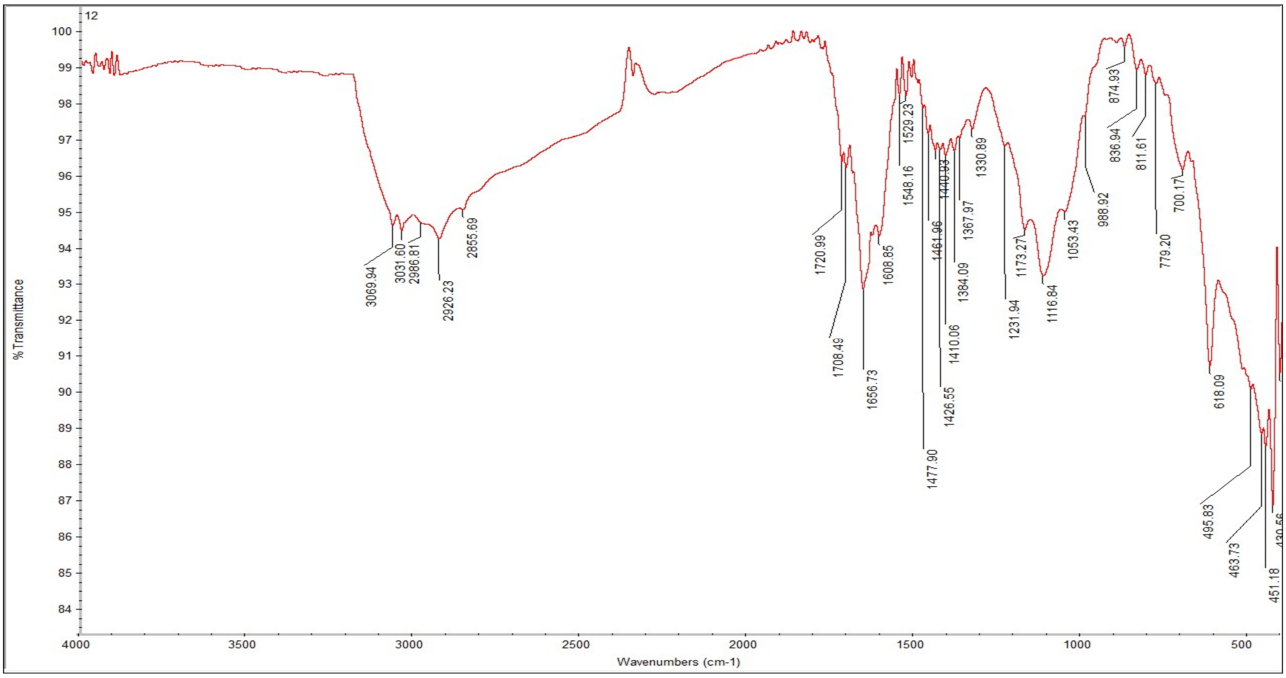

Figure S36. IR of Compound **12**

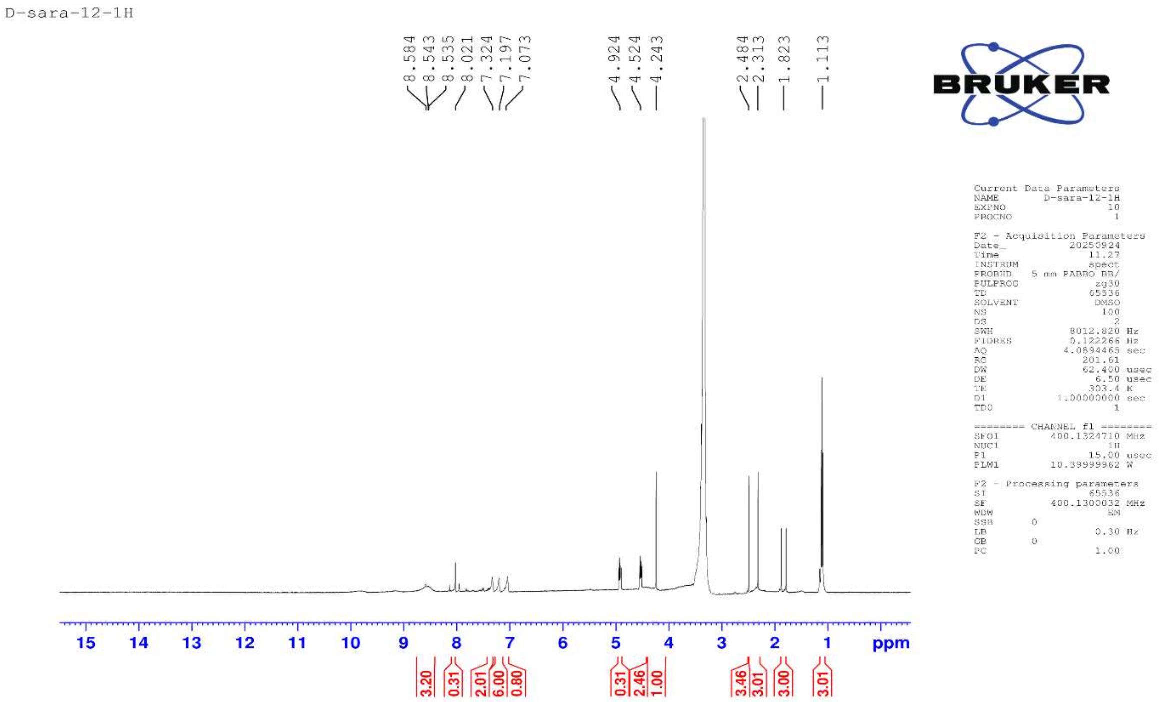

Figure S37. ^{1}H NMR spectrum (400 MHz, DMSO) of compound **12**

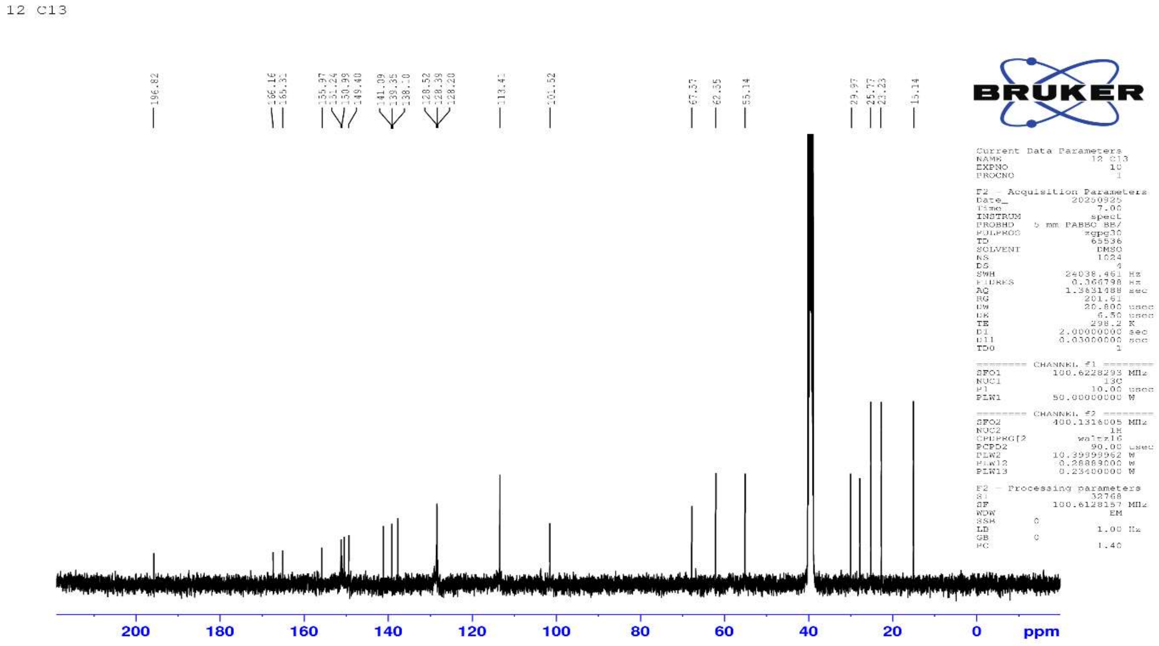

Figure S38. ^{13}C NMR spectrum (100 MHz, DMSO) of compound **12**

Characterization of Compound 13:

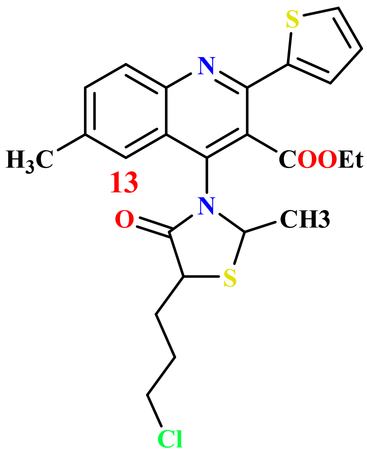

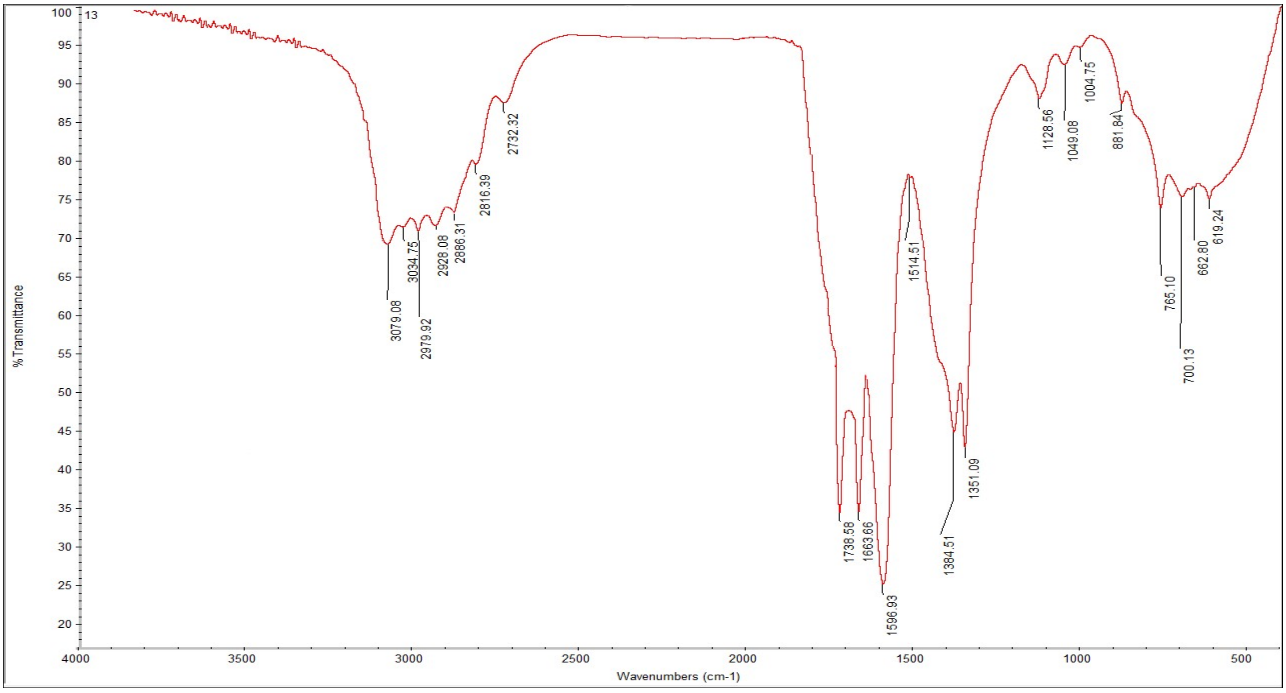

Figure S39. IR of Compound **13**

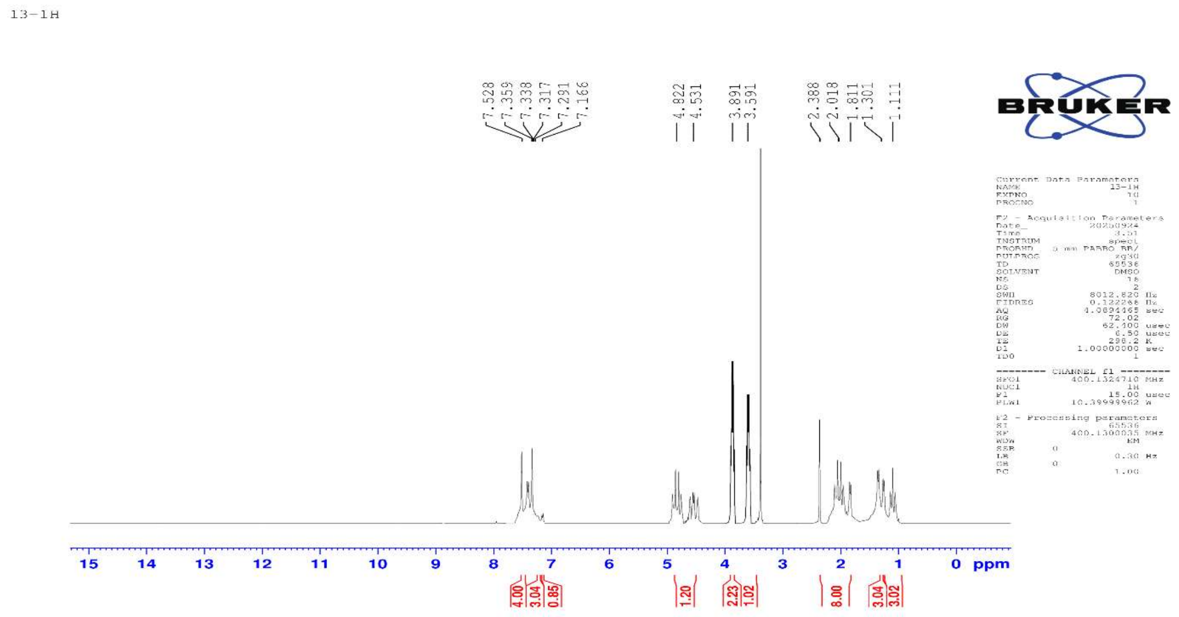

Figure S40. ^{1}H NMR spectrum (400 MHz, DMSO) of compound **13**

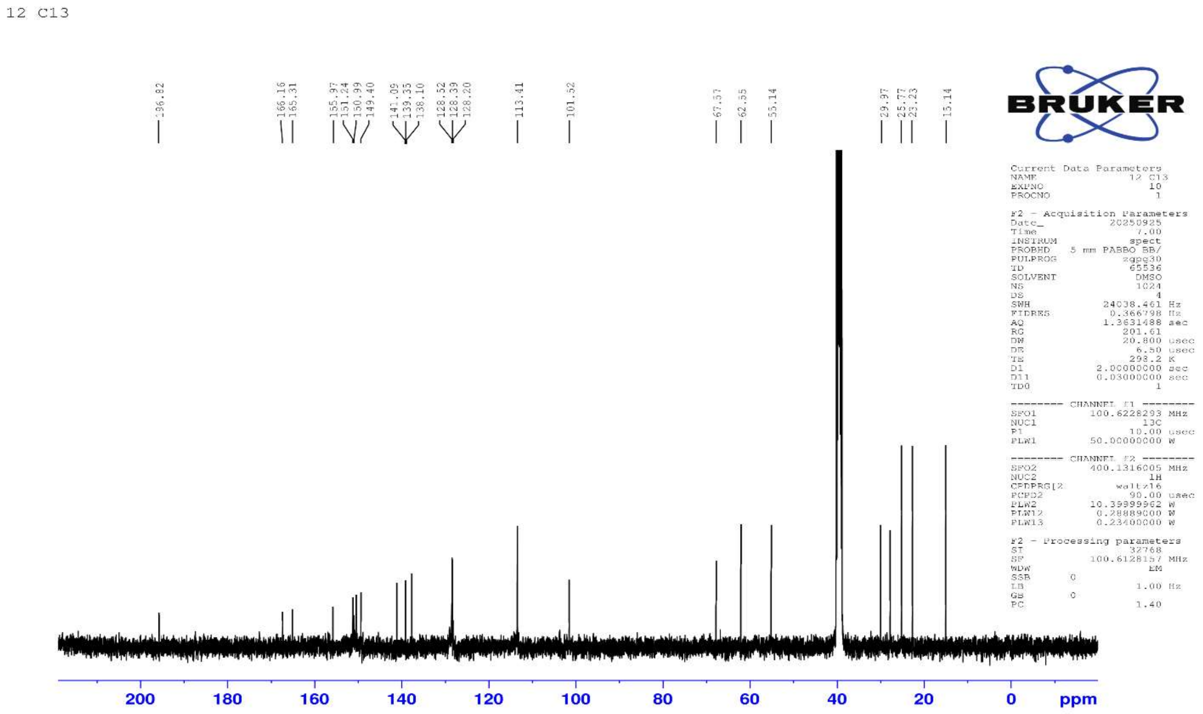

Figure S41. ^{13}C NMR spectrum (100 MHz, DMSO) of compound **13**

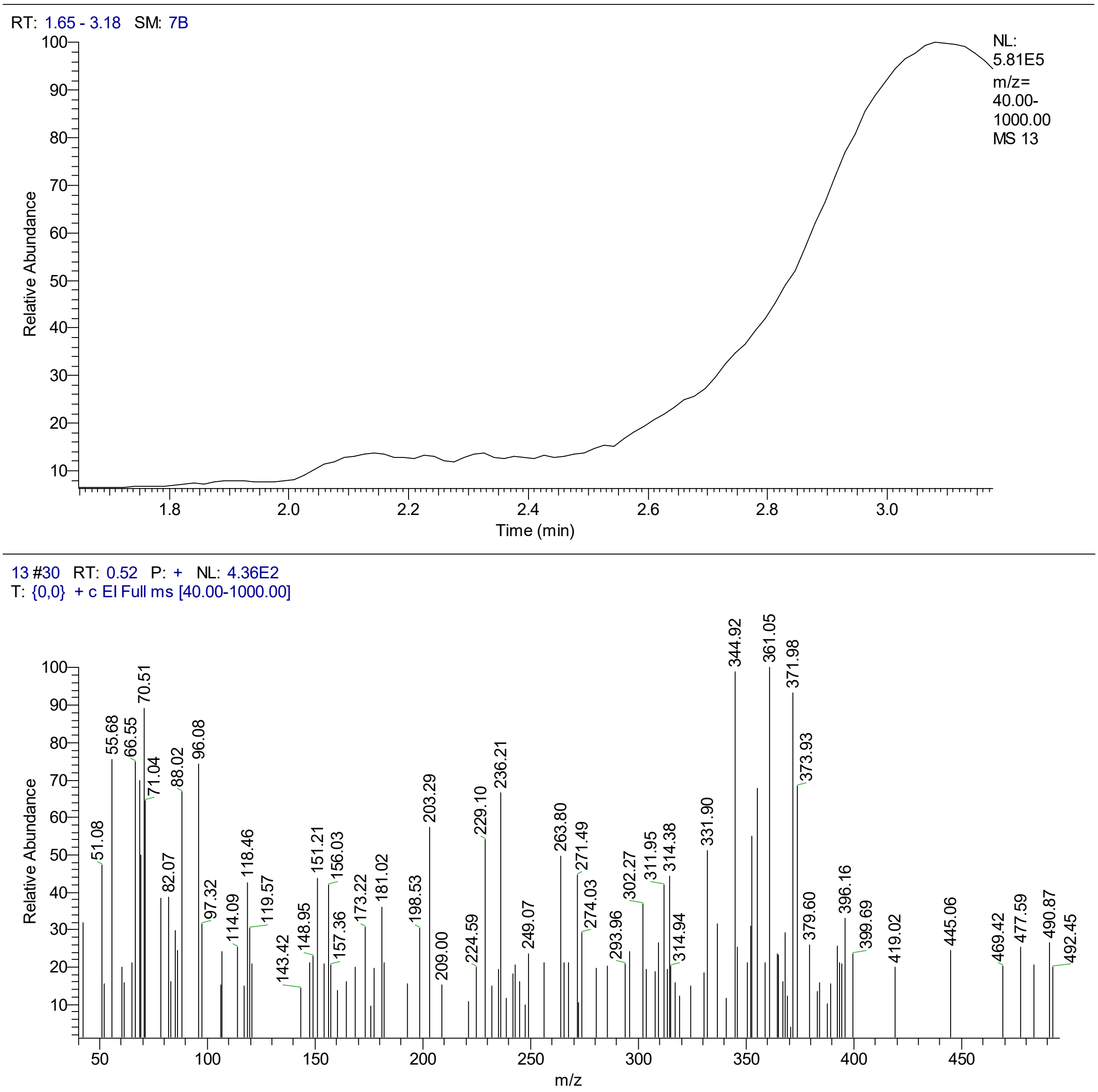

Figure S42. Mass spectrum of compound **13**

Characterization of Compound 14:

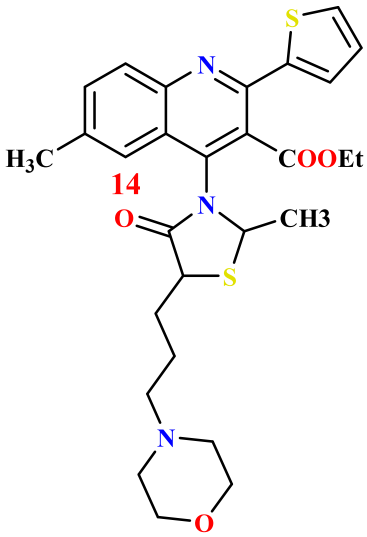

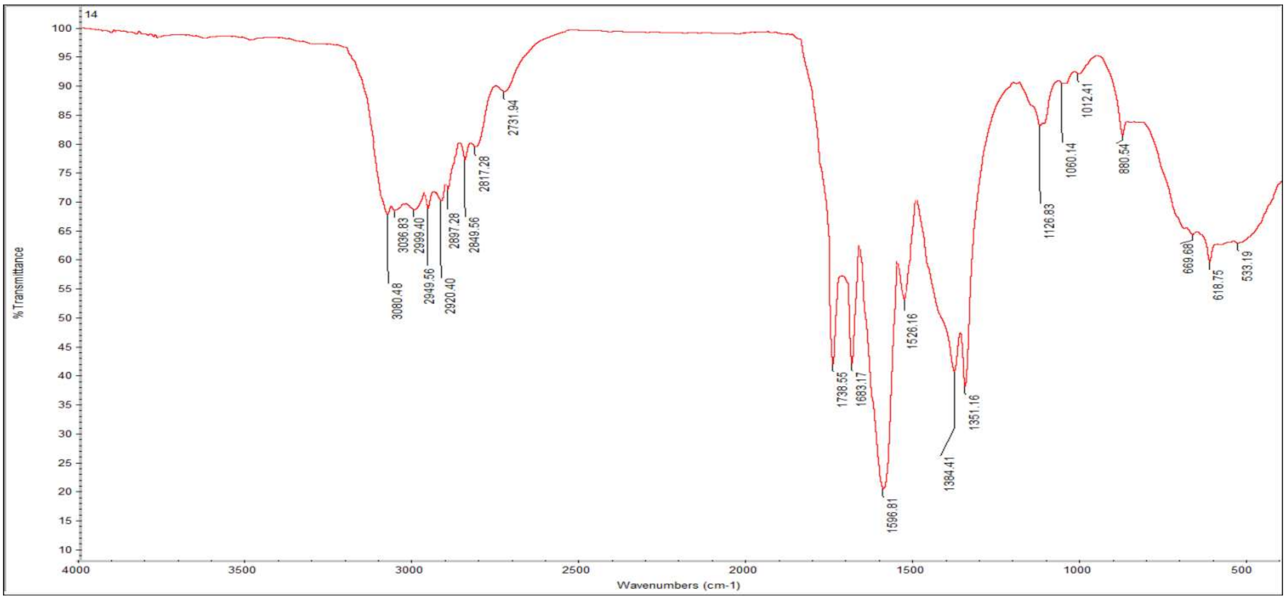

Figure S43. IR of Compound **14**

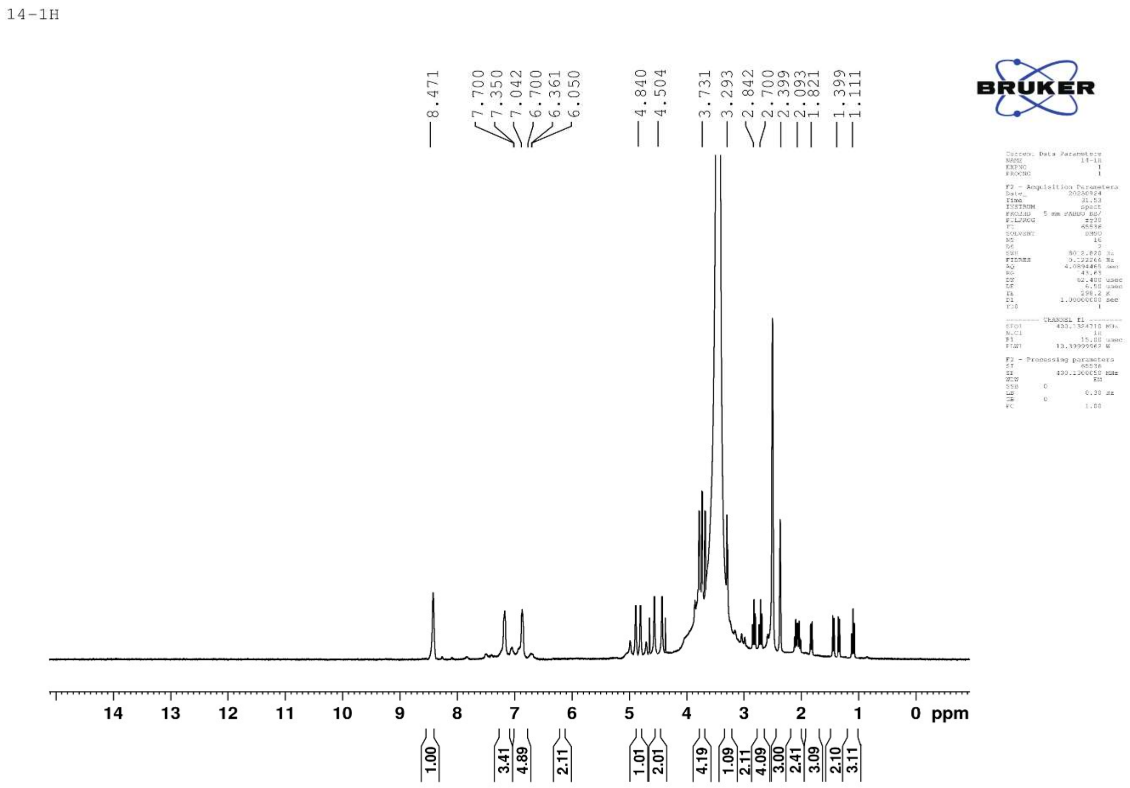

Figure S44. ^{1}H NMR spectrum (400 MHz, DMSO) of compound **14**

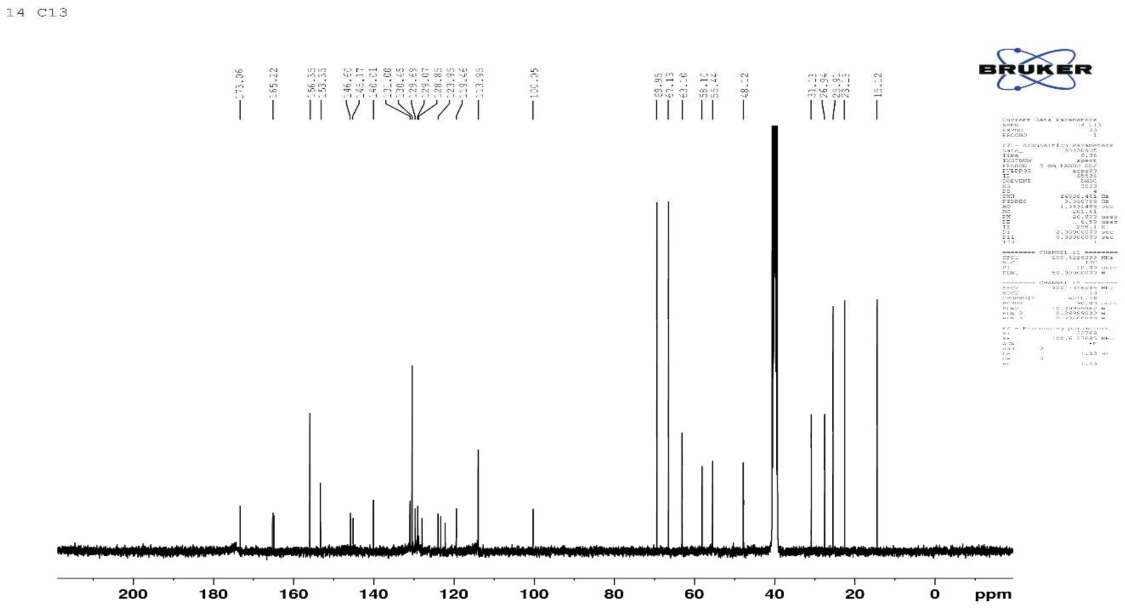

Figure S45. ^{13}C NMR spectrum (100 MHz, DMSO) of compound **14**

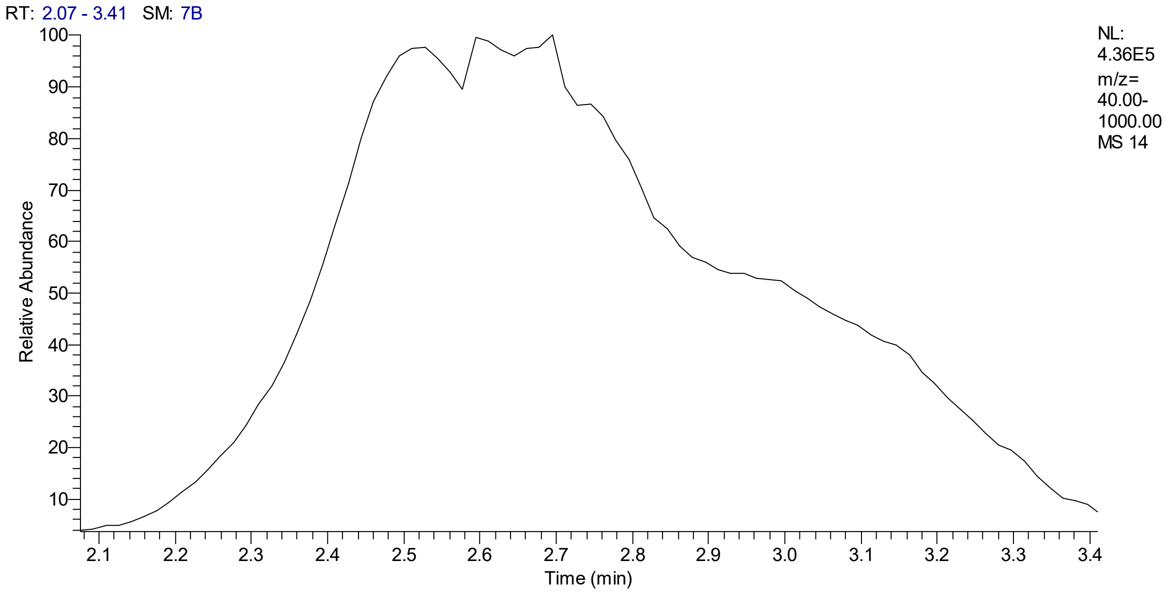

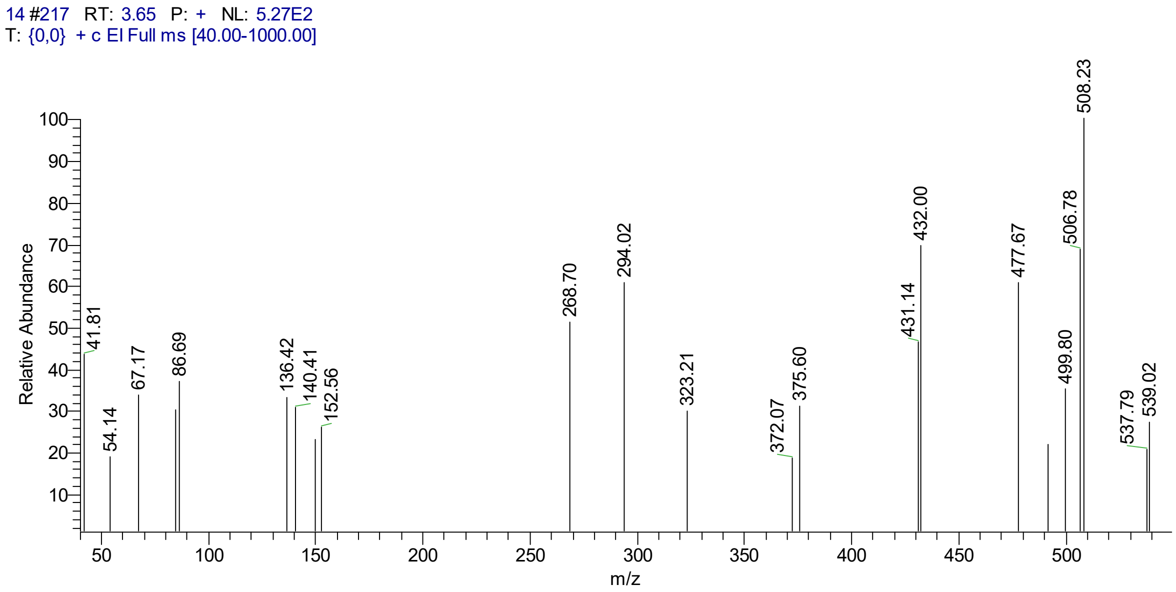

Figure S46. Mass spectrum of compound **14**

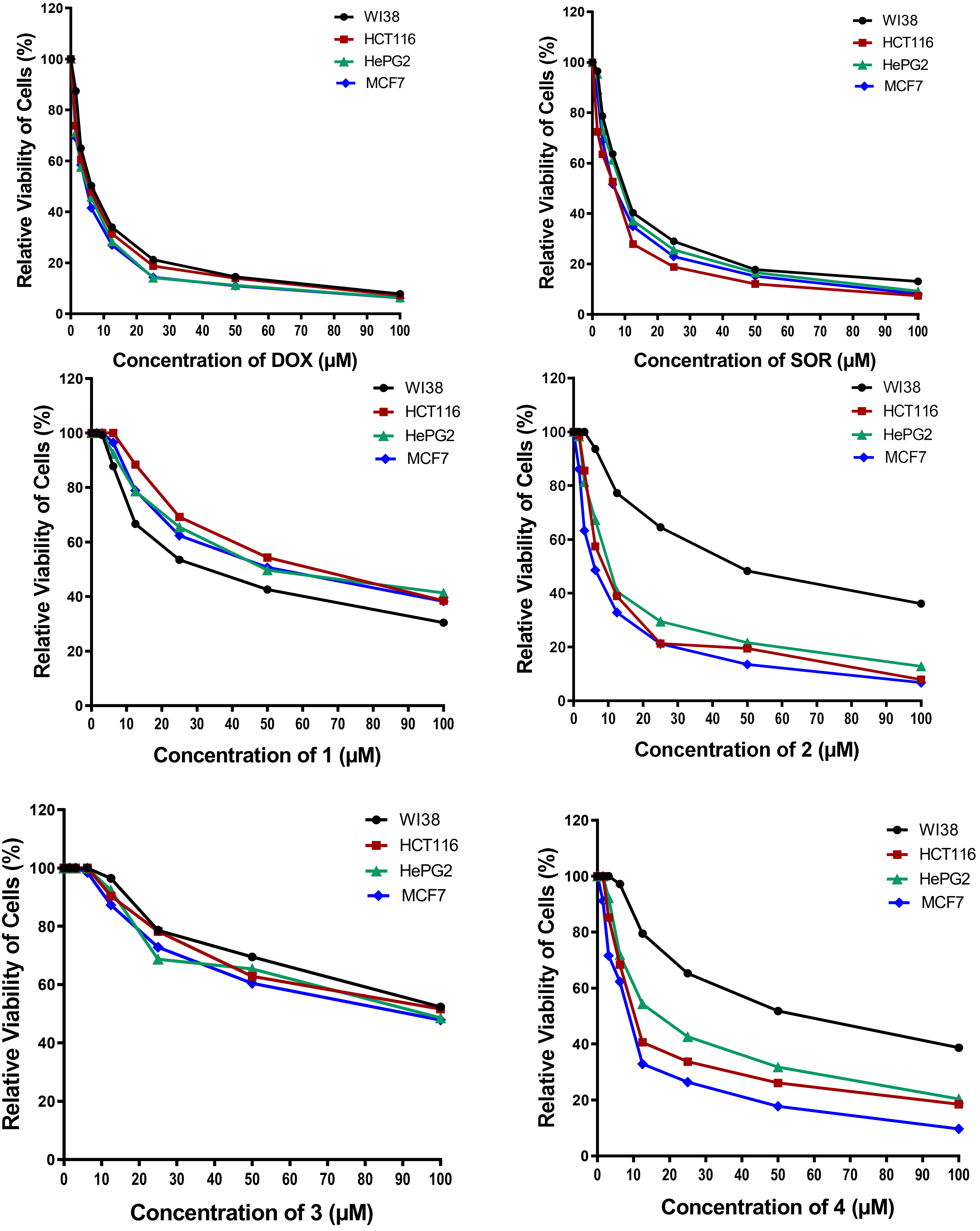
Relative Viability of Cells (%)
Concentration of DOX (µM)
Concentration of SOR (µM)
Concentration of 1 (µM)
Concentration of 2 (µM)
Concentration of 3 (µM)
Concentration of 4 (µM)
WI38
HCT116
HePG2
MCF7

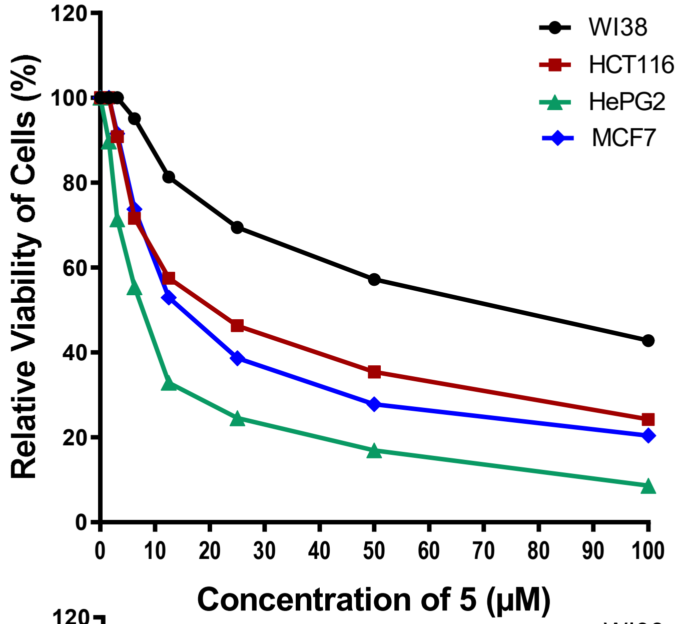
WI38
HCT116
HePG2
MCF7
Relative Viability of Cells (%)
Concentration of 5 (µM)

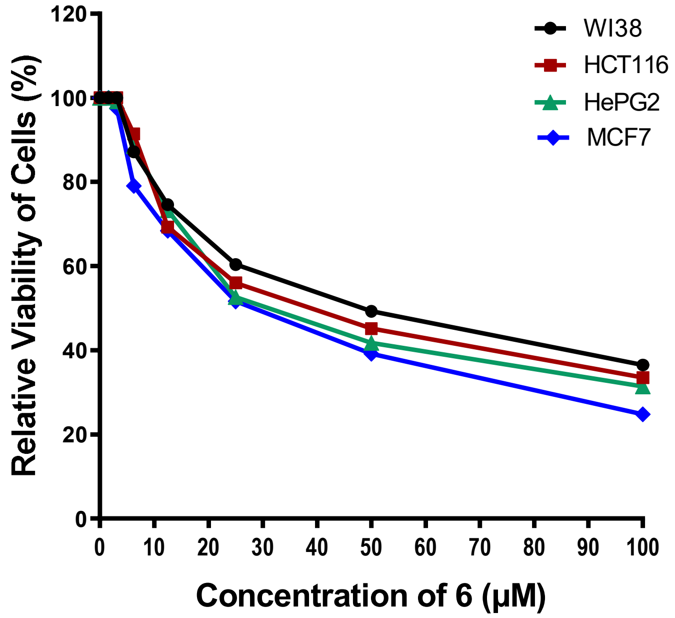
WI38
HCT116
HePG2
MCF7
Relative Viability of Cells (%)
Concentration of 6 (µM)

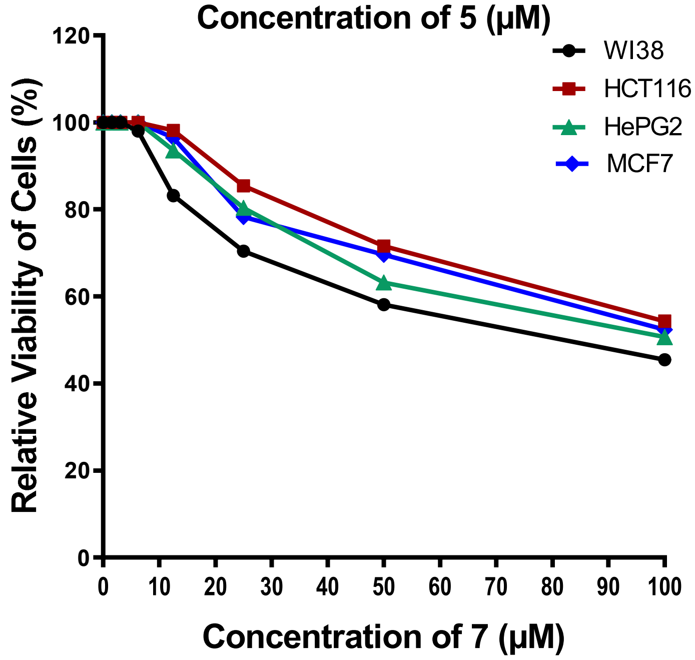
WI38
HCT116
HePG2
MCF7
Relative Viability of Cells (%)
Concentration of 7 (µM)

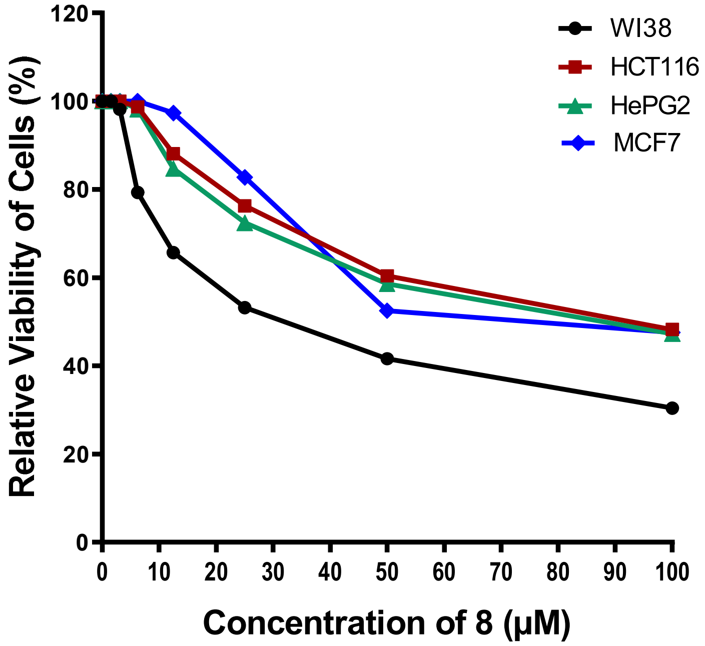
WI38
HCT116
HePG2
MCF7
Relative Viability of Cells (%)
Concentration of 8 (µM)

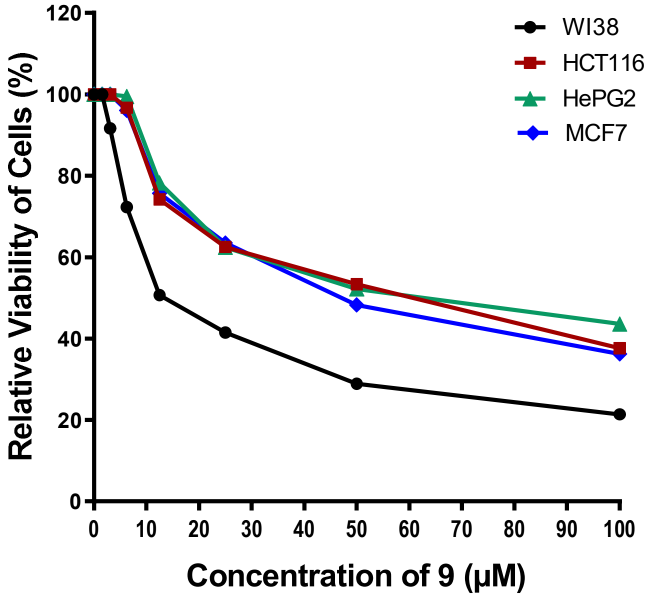
WI38
HCT116
HePG2
MCF7
Relative Viability of Cells (%)
Concentration of 9 (µM)

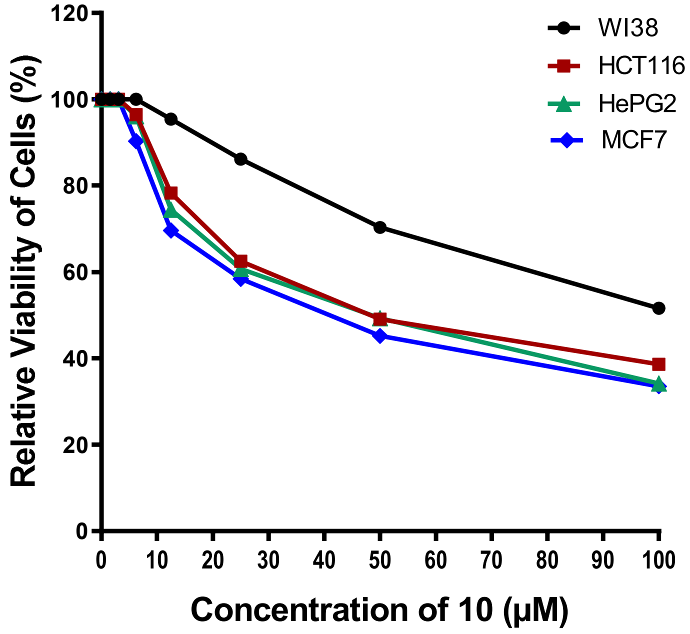
WI38
HCT116
HePG2
MCF7
Relative Viability of Cells (%)
Concentration of 10 (µM)

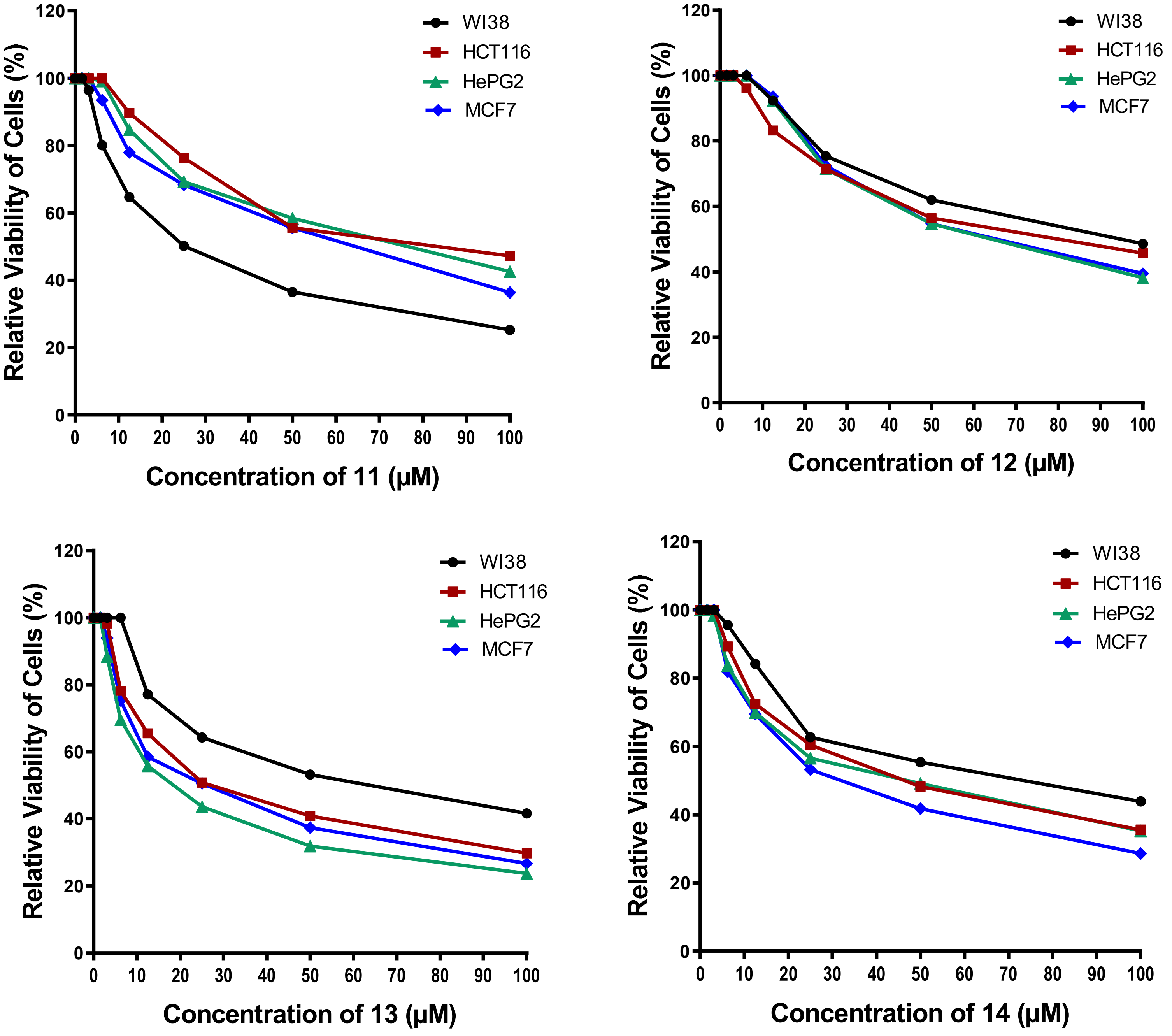

Figure S47. Dose–response IC_{50} curves of compounds **1**–**14**, Doxorubicin (Dox), and Sorafenib (Sor) against HepG-2, HCT-116, and MCF-7cell lines against normal WI-38 cell lines. % cell viability was plotted versus log concentration (μM), and IC_{50} values were derived using GraphPad Prism (non-linear regression, n = 3, mean ± SD).

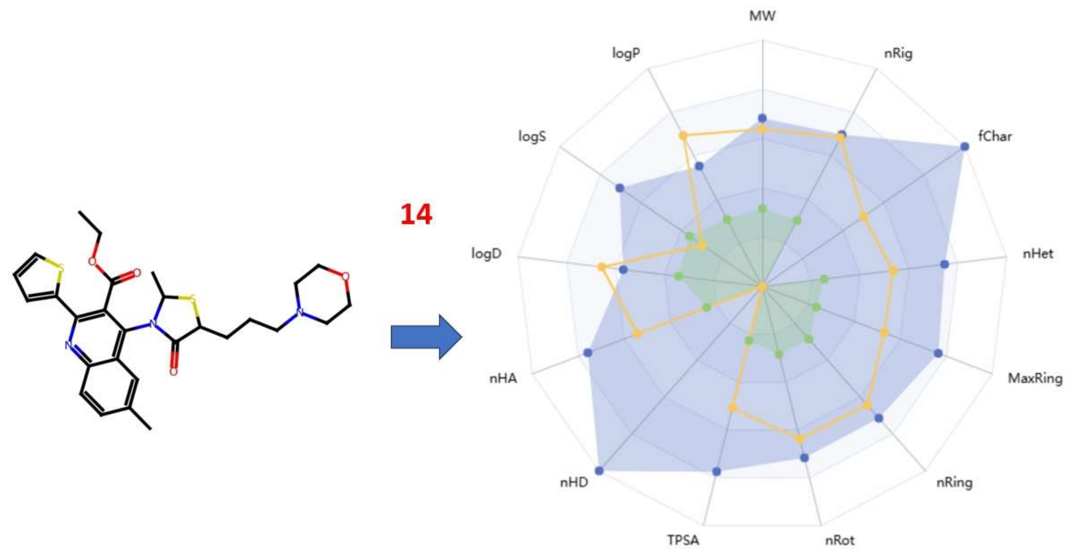

Figure S48. ADMET radar plot of compound **14** illustrating its physicochemical profile in relation to the optimal drug-like space. The blue shaded region represents the recommended range for orally active compounds, while the plotted profile of compound **14** reflects the balance between lipophilicity, polarity, hydrogen-bonding capacity, molecular flexibility, and structural complexity.

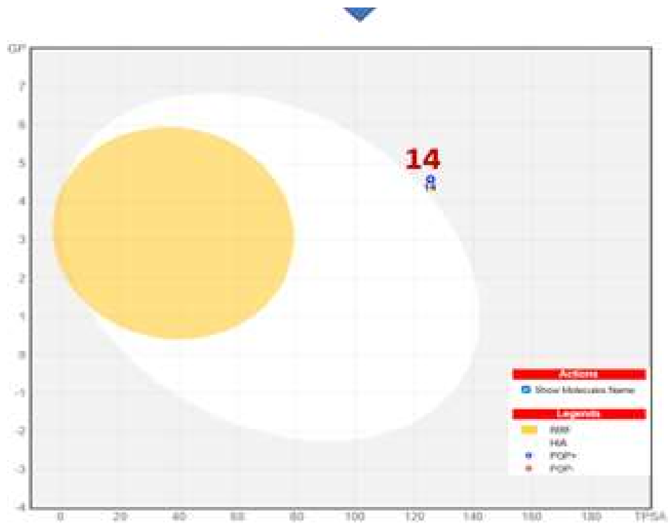

Figure S49. BOILED-EGG model illustrating the predicted gastrointestinal absorption and brain penetration behavior of compound **14**. The white region represents compounds with a high probability of passive gastrointestinal absorption, while the yellow region indicates the physicochemical space associated with blood–brain barrier penetration. The position of compound **14** (red marker) within the white region and outside the yellow region suggests favorable intestinal absorption with limited central nervous system exposure.

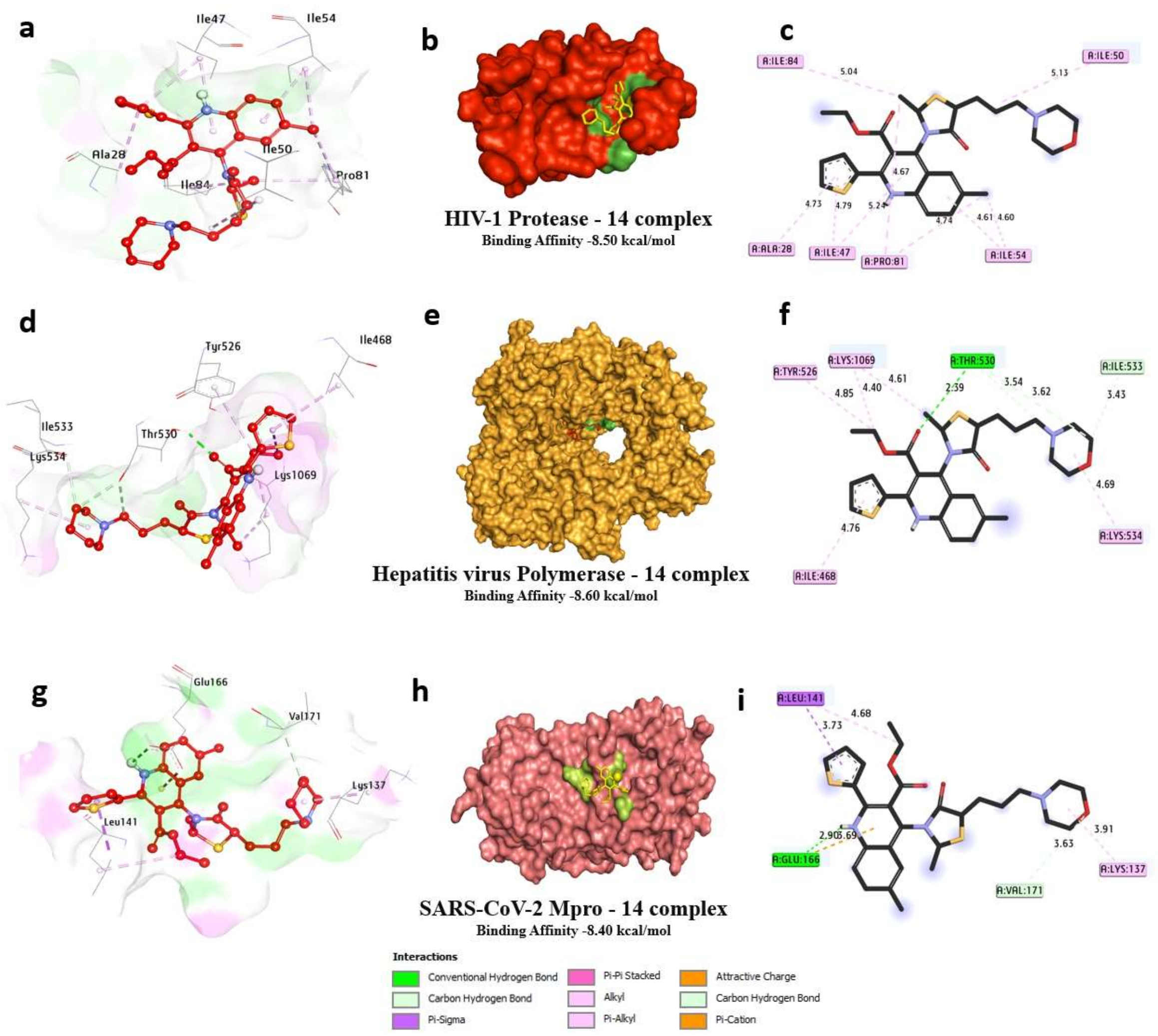

Figure S50. Molecular docking analysis of compound **14** with selected viral enzymes.

(**a-c**) 3D binding pose, surface representation, and 2D interaction map of compound **14** within the active site of HIV-1 protease (PDB ID: 3NU3). (**d-f**) 3D binding pose, surface representation, and 2D interaction map of compound **14** within the active site of hepatitis virus polymerase (PDB ID: 7LUF). (**g-i**) 3D binding pose, surface representation, and 2D interaction map of compound **14** within the active site of SARS-CoV-2 main protease (Mpro) (PDB ID: 7ZB7). The figures illustrate the binding orientations and key hydrogen bonding and hydrophobic interactions stabilizing the ligand enzyme complexes.
